# NMR-Guided Isolation of Anti-inflammatory Carabranolides from the Fruits of *Carpesium abrotanoides* L.

**DOI:** 10.1021/acs.jnatprod.4c00338

**Published:** 2024-07-10

**Authors:** Lu Fu, Can-Can Wang, Wenyue Tian, Zhiyan Liu, Meng-Yu Bao, Jiazheng Liu, Wei Zhang, Li-Ping Bai, Zhi-Hong Jiang, Guo-Yuan Zhu

**Affiliations:** State Key Laboratory of Quality Research in Chinese Medicine, Macau Institute for Applied Research in Medicine and Health, Macau University of Science and Technology, Taipa, Macau SAR 999078, People’s Republic of China

## Abstract

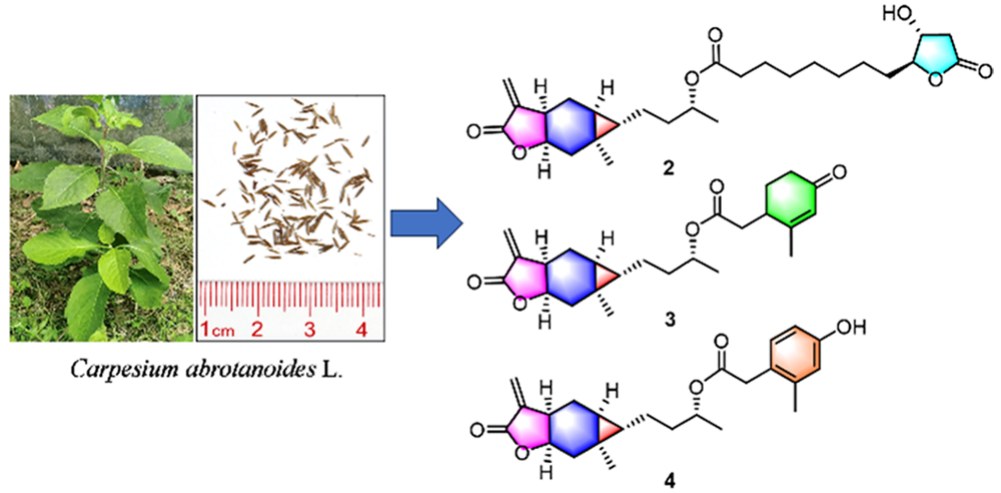

Carabranolides present characteristic NMR resonances for the cyclopropane moiety, which distinctly differ from those of other compounds and were used for an NMR-guided isolation in this study. As a result, 11 undescribed carabranolides (**1**–**11**), along with five known ones (**12**–**16**), were isolated from the fruits of *Carpesium abrotanoides* L. Compounds **1**–**11** are new esters of carabrol at C-4 with different carboxylic acids. Their structures were elucidated by HRESIMS and NMR spectroscopic data analysis. The biological evaluation showed that compounds **2**–**4**, **15**, and **16** exhibited significant inhibitory activity against LPS-induced NO release with an IC_50_ value of 5.6–9.1 μM and dose-dependently decreased iNOS protein expression in RAW264.7 cells.

The genus *Carpesium* (Compositae family) comprises about 21 species, which are widely distributed in Asia and Europe, particularly in the mountainous areas of Southwest China.^1^ In China, 17 species and three varieties are known, and six species (*Carpesium abrotanoides* L., *C. cernuum* L., *C. divaricatum* Sieb. et Zucc., *C. lipskyi* Winkl., *C. macrocephalum* Franch. et Sav., and *C. nepalense* Less) have been used as traditional Chinese medicines to treat cold, fever, sore throat, tonsillitis, wound bleeding, swollen poison, herpes zoster, and snake bites.^2,3^ Sesquiterpene lactones and sesquiterpene dimers were reported as characteristic constituents of this genus with diverse biological activities including cytotoxic,^4^ anti-inflammatory,^5^ antibacterial,^6^ and insecticidal^7^ activities.

*C*. *abrotanoides*, a perennial plant, has been extensively used as a medicinal plant for treating sore throat, tonsillitis, bronchitis, trauma, bleeding, and snake and insect bites in China, Korea, and Japan.^7−10^ Fruits of this species serve as a significant anthelmintic in traditional Chinese medicine for ascariasis, pinworm disease, and tapeworm infections.^11^ Although over 130 compounds have been identified from *C. abrotanoides*,^12−14^ the bioactive compounds from its fruits have rarely been investigated.^7,13^ In a recent study, seven new cytotoxic sesquiterpene lactone dimers with a carabrol unit were isolated from the fruits of *C. abrotanoides*.^13^ Our preliminary NMR data analysis revealed that a number of unknown carabrol derivatives were present in the extract of the fruits of *C. abrotanoides*. To identify these unknown carabranolides and their bioactivities, an NMR-guided isolation was performed on the fruits of *C. abrotanoides*, which led to the isolation of 11 new carabranolides (**1**–**11**), along with five known derivatives (**12**–**16**). The anti-inflammatory activity of these sesquiterpenes was also evaluated, revealing that compounds **2**–**4**, **15**, and **16** significantly inhibited LPS-induced NO release with an IC_50_ value of 5.6–9.1 μM and dose-dependently decreased iNOS protein expression in RAW264.7 cells.
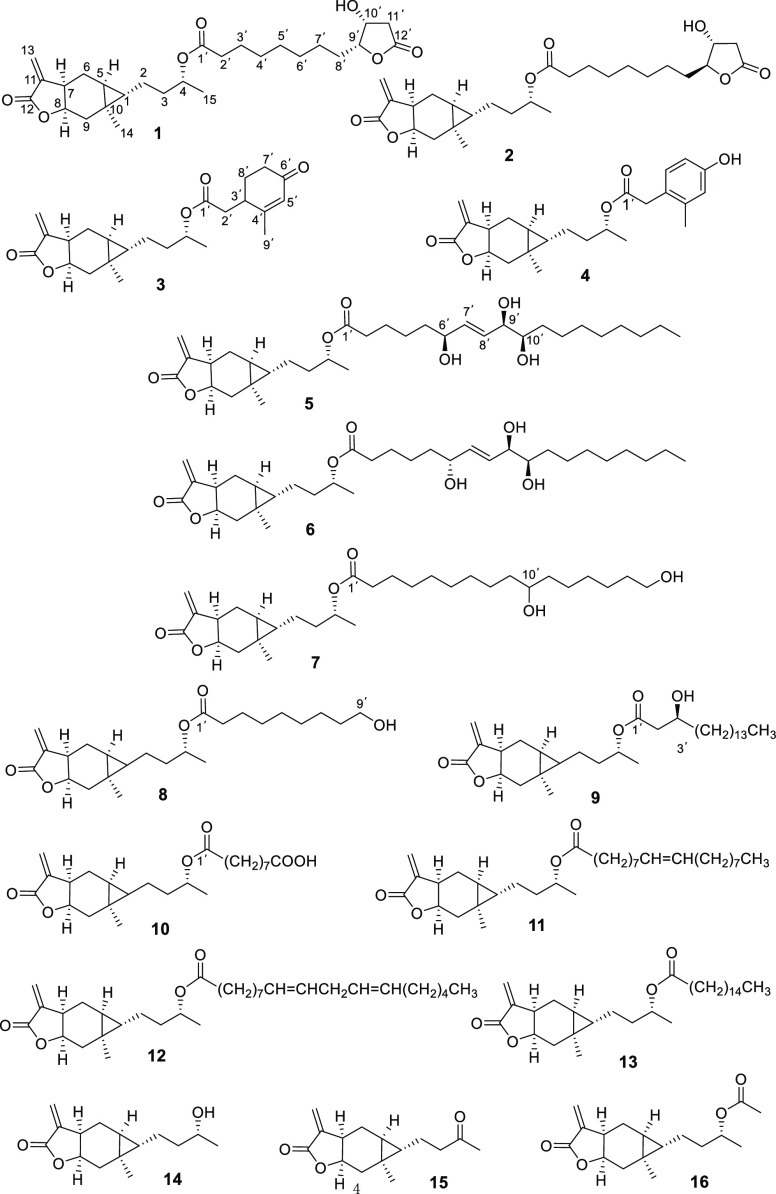


## Results and Discussion

**Figure 1:**
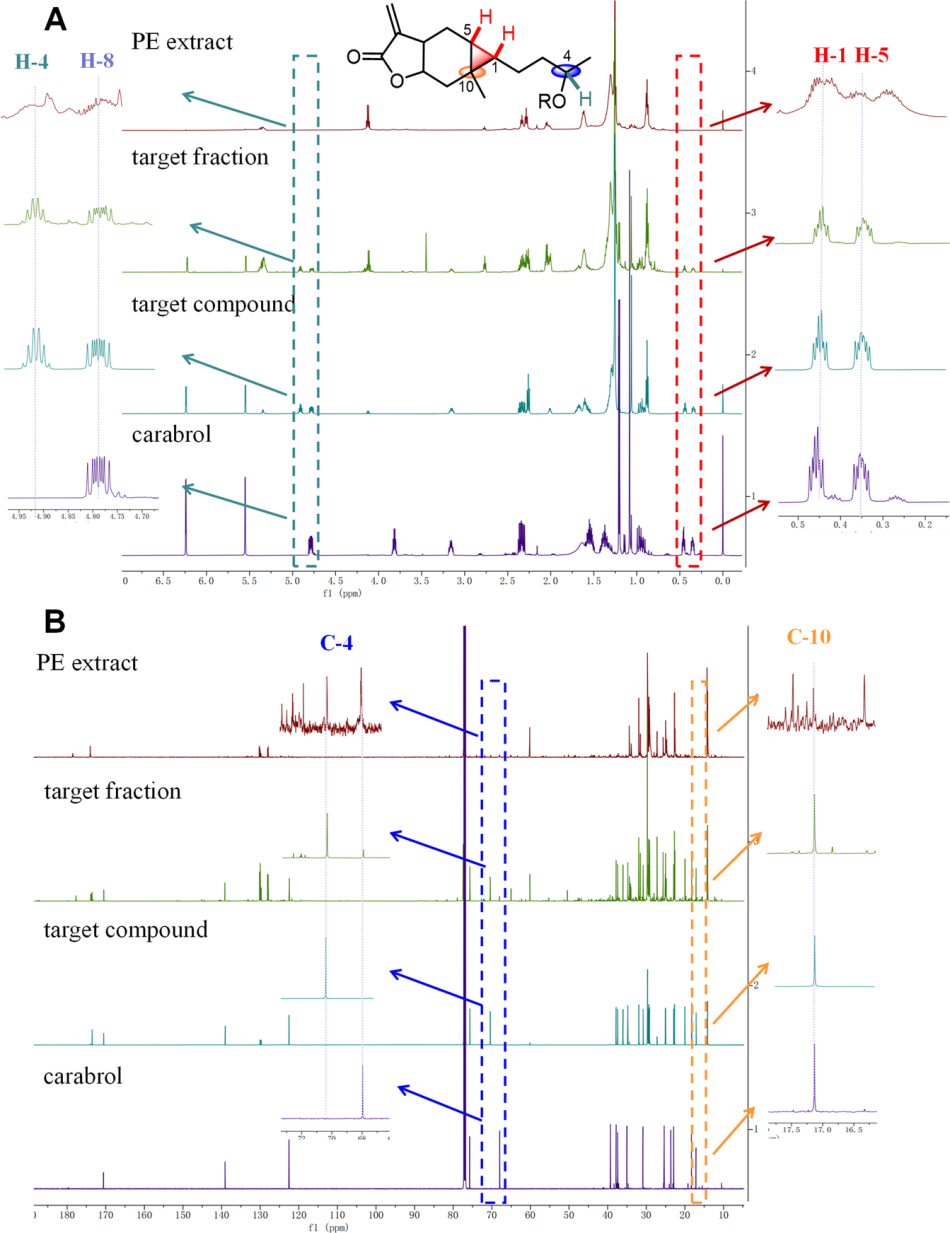
^1^H (A) and ^13^C (B) NMR spectra of the PE fraction of the EtOH extract, the target fraction, a target compound, and carabrol (**14**) from the fruits of *C. abrotanoides*.

Carabranolides present two NMR signals of characteristic methines at δ_H_ ∼0.44 and ∼0.34 (H-1 and H-5) as well as a quaternary carbon at δ_C_ ∼17.1 (C-10) in their ^1^H and ^13^C NMR spectra for the cyclopropane moiety.^4,6,15^ Chemical shifts of H-4 and C-4 in these compounds shifted downfield from δ_H_ ∼3.8 to ∼4.9 and from δ_C_ ∼68 to ∼71, respectively, due to the esterification of the hydroxy group at C-4 of carabrol.^16^ Therefore, an NMR-guided isolation method for carabranolide derivatives was established in this study. Signals for H-1, H-4, H-5, C-4, and C-10 were observed in ^1^H and ^13^C NMR spectra (Figure 1) of the ethanolic extract of *C. abrotanoides* fruits. A series of fractionation steps were then performed and monitored by NMR analyses. Eleven undescribed carabranolides (**1**–**11**) were isolated, along with the known compounds carabrol-4-*O*-linoleate (**12**) and carabrol-4-*O*-palmitate (**13**),^17^ carabrol (**14**),^18^ carabrone (**15**),^15^ and (4*S*)-acetyloxyl-11(13)-carabren-8β,12-olide (**16**),^19^ identified by comparison of experimental and reported spectroscopic data.

Compound **1** was obtained as a yellow oil. Its molecular formula was determined as C_27_H_40_O_7_ by (+)-HRESIMS analysis at *m*/*z* 477.2833 [M + H]^+^ (calcd for 477.2847). The IR spectrum of **1** showed the presence of carbonyl (1759 cm^–1^) and hydroxyl (3487 cm^–1^) bands. The ^1^H NMR spectrum of **1** (Table 2) exhibited ^1^H signals for two methyl groups at δ_H_ 1.06 (s, H-14) and 1.21 (d, *J* = 6.2 Hz, H-15), one exocyclic olefinic methylene at δ_H_ 5.56 (d, *J* = 2.5 Hz, H-13a) and 6.24 (d, *J* = 2.5 Hz, H-13b), and two methines of a characteristic cyclopropane moiety at δ_H_ 0.34 (ddd, *J* = 9.1, 7.0, 4.1 Hz, H-5) and 0.44 (td, *J* = 7.2, 4.1 Hz, H-1). The ^13^C NMR and DEPT135 spectra showed signals for 27 carbons (Table 1), including two methyl groups (δ_C_ 18.3 and 20.0, C-14 and C-15, respectively), 13 methylenes (one exocyclic methylene at δ_C_ 122.7, C-13), seven methines (four oxygenated methines at δ_C_ 69.0, 70.6, 75.8, and 84.7; C-10′, C-4, C-8, and C-9′), and five quaternary carbons (three carbonyl carbons at δ_C_ 170.6, 173.5, and 175.4, C-12, C-1′, and C-12′). ^1^H and ^13^C NMR spectroscopic data of **1** suggested the presence of a carabranolide unit and a furanoctanoic acid group in **1**.

**Table 1:** ^13^C NMR (150 MHz) Data of **1**–**11** (CDCl_3_)

no.	**1**	**2**	**3**	**4**	**5**	**6**	**7**	**8**	**9**	**10**	**11**
1	34.7, CH	34.7, CH	34.6, CH	34.7, CH	34.8, CH	34.8, CH	34.8, CH	34.8, CH	34.7, CH	34.8, CH	34.8, CH
2	25.0, CH_2_	25.0, CH_2_	25.0, CH_2_	24.9, CH_2_	25.0, CH_2_	25.0, CH_2_	25.0, CH_2_	25.0, CH_2_	25.0, CH_2_	25.0, CH_2_	25.0, CH_2_
3	35.9, CH_2_	35.9, CH_2_	35.9, CH_2_	35.9, CH_2_	36.0, CH_2_	36.0, CH_2_	36.0, CH_2_	36.0, CH_2_	36.0, CH_2_	36.0, CH_2_	36.0, CH_2_
4	70.6, CH	70.6, CH	71.5, CH	71.2, CH	70.5, CH	70.5, CH	70.4, CH	70.5, CH	71.2, CH	70.5, CH	70.4, CH
5	22.9, CH	22.9, CH	22.9, CH	22.9, CH	22.9, CH_2_	22.9, CH	22.9, CH	22.9, CH	22.9, CH	22.9, CH	22.9, CH_2_
6	30.8, CH_2_	30.8, CH_2_	30.8, CH_2_	30.8, CH_2_	30.8, CH_2_	30.8, CH_2_	30.8, CH_2_	30.8, CH_2_	30.8, CH_2_	30.8, CH_2_	30.8, CH_2_
7	37.8, CH	37.8, CH	37.8, CH	37.8, CH	37.8, CH	37.8, CH	37.8, CH	37.8, CH	37.8, CH	37.8, CH	37.8, CH
8	75.8, CH	75.8, CH	75.6, CH	75.7, CH	75.7, CH	75.7, CH	75.7, CH	75.7, CH	75.6, CH	75.7, CH	75.7, CH
9	37.4, CH_2_	37.4, CH_2_	37.3, CH_2_	37.3, CH_2_	37.4, CH_2_	37.4, CH_2_	37.4, CH_2_	37.4, CH_2_	37.4, CH_2_	37.4, CH_2_	37.4, CH_2_
10	17.1, C	17.1, C	17.1, C	17.0, C	17.1, C	17.1, C	17.1, C	17.1, C	17.1, C	17.1, C	17.1, C
11	139.0, C	139.0, C	139.0, C	139.1, C	139.0, C	139.0, C	139.0, C	139.0, C	139.0, C	139.0, C	139.0, C
12	170.6, C	170.7, C	170.5, C	170.7, C	170.6, C	170.6, C	170.6, C	170.6, C	170.5, C	170.6, C	170.5, C
13	122.7, CH_2_	122.7, CH_2_	122.6, CH_2_	122.6, CH_2_	122.6, CH_2_	122.6, CH_2_	122.6, CH_2_	122.6, CH_2_	122.6, CH_2_	122.6, CH_2_	122.5, CH_2_
14	18.3, CH_3_	18.3, CH_3_	18.3, CH_3_	18.2, CH_3_	18.3, CH_3_	18.3, CH_3_	18.3, CH_3_	18.3, CH_3_	18.3, CH_3_	18.2, CH_3_	18.2, CH_3_
15	20.0, CH_3_	20.0, CH_3_	20.0, CH_3_	20.0, CH_3_	20.0, CH_3_	20.0, CH_3_	20.0, CH_3_	20.0, CH_3_	19.9, CH_3_	20.0, CH_3_	20.0, CH_3_
1′	173.5, C	173.5, C	171.5, C	171.4, C	173.5, C	173.5, C	173.5, C	173.5, C	172.7, C	173.5, C	173.5, C
2′	34.6, CH_2_	34.6, CH_2_	36.5, CH_2_	38.8, CH_2_	34.7, CH_2_	34.7, CH_2_	34.7, CH_2_	34.7, CH_2_	41.6, CH_2_	34.7, CH_2_	34.7, CH_2_
3′	24.9, CH_2_	24.9^a^, CH_2_	36.4, CH	125.3, C	25.1^a^, CH_2_	25.0^a^, CH_2_	25.1, CH_2_	25.0, CH_2_	68.1, CH	25.0, CH_2_	25.1, CH_2_
4′	29.2^b^, CH_2_	28.9^b^, CH_2_	163.1, C	138.4, C	25.1^a^, CH_2_	25.1^a^, CH_2_	29.1^b^, CH_2_	29.2^b^, CH_2_	36.6, CH_2_	28.9^b^, CH_2_	29.5^b^, CH_2_
5′	29.0^b^, CH_2_	29.0^b^, CH_2_	127.7, CH	117.2, CH	37.2, CH_2_	37.4, CH_2_	29.2^b^, CH_2_	29.1^b^, CH_2_	25.5, CH_2_	28.8^b^, CH_2_	29.3^b^, CH_2_
6′	28.9^b^, CH_2_	28.9^b^, CH_2_	198.8, C	154.6, C	72.3, CH	72.1, CH	29.4^b^, CH_2_	29.2^b^, CH_2_	29.6^b^, CH_2_	28.9^b^, CH_2_	29.2^b^, CH_2_
7′	25.4, CH_2_	25.1^a^, CH_2_	34.1, CH_2_	112.8, CH	136.5, CH	136.5, CH	29.5^b^, CH_2_	25.7, CH_2_	29.6^b^, CH_2_	24.7, CH_2_	29.^b^, CH_2_
8′	28.3, CH_2_	33.1, CH_2_	27.4, CH_2_	131.3, CH	129.9, CH	129.5, CH	29.6^b^, CH_2_	32.7, CH_2_	29.7^b^, CH_2_	34.1, CH_2_	27.2^a^, CH_2_
9′	84.7, CH	87.5, CH	22.7, CH_3_	19.7, CH_3_	75.5, CH	75.6, CH	37.5, CH_2_	63.0, CH_2_	29.7^b^, CH_2_	179.0, C	130.0, CH
10′	69.0, CH	71.8, CH			74.5, CH	74.6, CH	72.0, CH		29.7^b^, CH_2_		129.7, CH
11′	39.4, CH_2_	37.7, CH_2_			32.9, CH_2_	32.9, CH_2_	37.4, CH_2_		29.7^b^, CH_2_		27.1^a^, CH_2_
12′	175.4, C	174.8, C			25.0^a^, CH_2_	25.0^a^, CH_2_	25.7^a^, CH_2_		29.6^b^, CH_2_		29.7^b^, CH_2_
13′					29.0^b^, CH_2_	29.0^b^, CH_2_	25.6^a^, CH_2_		29.4^b^, CH_2_		29.1^b^, CH_2_
14′					29.2^b^, CH_2_	29.0^b^, CH_2_	25.6^a^, CH_2_		29.7^b^, CH_2_		29.1^b^, CH_2_
15′					29.4^b^, CH_2_	29.4^b^, CH_2_	32.7, CH_2_		31.9, CH_2_		29.1^b^, CH_2_
16′					31.7, CH_2_	31.7, CH_2_	63.0, CH_3_		22.7, CH_2_		31.9, CH_2_
17′					22.6, CH_2_	22.6, CH_2_			14.1, CH_3_		22.7, CH_2_
18′					14.0, CH_3_	14.0, CH_3_					14.1, CH_3_

a,b Assignments may be interchanged within the same column.

**Table 2:** ^1^H NMR (600 MHz) Data of **1**–**5** (CDCl_3_)

no.	**1**	**2**	**3**	**4**	**5**
1	0.44, td (7.2, 4.1)	0.45, ttd (7.0, 3.9, 2.1)	0.44, td (7.2, 4.1)	0.36, td (7.1, 4.1)	0.44, td (7.3, 4.1)
2	1.31, m	1.31, m	1.31, m	1.18, m	1.30, m
	1.61, m	1.64, m	1.62, m	1.29, m	1.60, m
3	1.57, m	1.57, m	1.58, m	1.56, m	1.57, m
	1.69, m	1.69, m	1.71, m	1.63, m	1.69, m
4	4.91, q (6.3)	4.92, q (6.3)	4.96, qd (6.3, 6.3)	4.90, ddd (7.4, 6.3, 5.3)	4.92, q (6.3)
5	0.34, ddd (9.1, 7.0, 4.1)	0.34, ddd (9.0, 7.0, 4.1)	0.35, ddd (9.2, 7.0, 4.1)	0.26, ddd (8.8, 7.1, 4.1)	0.34, ddd (8.7, 7.1, 4.1)
6	0.90, m	0.92, m	0.94, ddd (14.5, 12.2, 9.2)	0.87, m	0.91, m
	2.35, m	2.37, m	2.36, dd (14.5, 7.0)	2.33, m	2.35, m
7	3.16, m	3.17, m	3.16, m	3.14, m	3.15, m
8	4.79, ddd (12.3, 9.2, 6.1)	4.79, ddd (11.5, 9.1, 6.1)	4.78, ddd (11.5, 8.8, 6.1)	4.76, ddd (11.5, 8.8, 6.2)	4.78, ddd (11.5, 8.8, 6.1)
9	0.96, m	0.96, m	0.97, dd (13.9, 11.5)	0.90, m	0.97, m
	2.33, m	2.32, m	2.32, dd (13.9, 6.1)	2.29, m	2.33, m
13	5.56, d (2.5)	5.56, d (2.6)	5.55, d (2.5)	5.55, d (2.5)	5.55, d (2.4)
	6.24, d (2.5)	6.24, d (2.6)	6.24, d (2.5)	6.24, d (2.5)	6.24, d (2.4)
14	1.06, s	1.06, s	1.07, s	1.00, s	1.07, s
15	1.21, d (6.2)	1.21, d (6.2)	1.24, d (6.2)	1.20, d (6.3)	1.21, d (6.2)
2′	2.27, t (7.4)	2.27, t (7.4)	2.42, dd (15.5, 9.7)	3.52, d (13.7)	2.26, m
			2.58, dd (15.5, 4.5)		
3′	1.62, m	1.62, m	2.81, dq (9.7, 4.8)		1.63, m
4′	1.32, m	1.32, m			1.63, m
5′	1.32, m	1.32, m	5.88, br s	6.65, d (2.7)	1.57, m
6′	1.32, m	1.32, m			4.13, q (6.5)
7′	1.62, m	1.62, m	2.35, dd (10.2, 7.2)	6.61, dd (8.2, 2.7)	5.79, dd (15.5, 6.5)
			2.46, dd (10.2, 4.9)		
8′	1.71, m	1.57, m	1.89, dtt (13.5, 7.0, 5.1)	7.04, d (8.2)	5.68, dd (15.5, 6.2)
	1.86, m	1.63, m	2.12, ddt (13.5, 10.0, 4.9)		
9′	4.35, ddd (8.8, 4.5, 3.8)	4.33, ddt (8.3, 6.7, 3.1)	1.97, s	2.26, s	3.93, t (6.2)
10′	4.49, t (4.5)	4.28, dt (6.7, 2.9)			3.46, ddd (9.1, 6.2, 2.8)
11′	2.56, dd (17.7, 1.0)	2.52, dd (17.9, 4.1)			1.41, m
	2.81, dd (17.7, 4.5)	2.83, dd (17.9,6.7)			1.50, m
12′					1.63, m
13′					1.31, m
14′					1.31, m
15′					1.31, m
16′					1.29, m
17′					1.30, m
18′					0.89, t (6.9)

**Figure 2:**
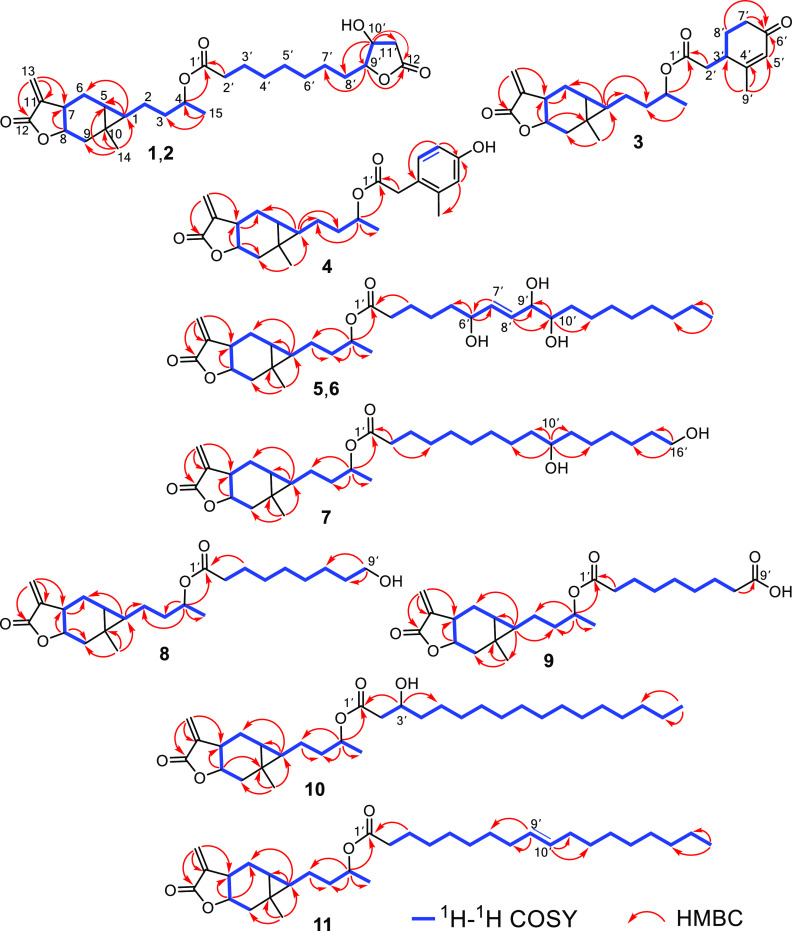
Key ^1^H–^1^H COSY and HMBC correlations of **1**–**11**.

The spin system H-15–H-4–H-3–H-2–H-1–H-5–H-6–H-7–H-8–H-9 was observed in the COSY spectrum. HMBC correlations from H-13 to C-7, C-11, and C-12 and from H-14 to C-1, C-5, C-9, and C-10 (Figure 2) established the carabrol unit of **1** as the same in carabrol (**14**).^18^ The remaining ^1^H and ^13^C NMR data of **1** were almost identical to those of lonfuranacid A, a natural furanoctanoic acid,^20^ indicating **1** has a lonfuranacid A unit, which was supported by the ^1^H–^1^H correlations of H-2′/H-3′/H-4′/H-5′/H-6′/H-7′/H-8′/H-9′/H-10′/H-11′ and HMBC correlations from H-2′ to C-1′, H-10′ to C-8′ and C-12′, and H-9′ to C-12′ (Figure 2). By comparing with the NMR data of **14**, the chemical shift of H-4 in **1** shifted from δ_H_ 3.82 (**14**) to 4.92 (**1**), suggesting the hydroxy group at C-4 of carabrol was esterified by the lonfuranacid A moiety in **1**. This hypothesis was confirmed by analysis of HMBC data, which showed a correlation between H-4 (δ_H_ 4.91) and C-1′ (δ_C_ 173.5). NOE correlations observed for H-4/H-1/H-9β, H-5/H-14/H-9α/H-7, and H-14/H-8 (Figure S1) further confirmed that **1** has the same relative configuration as carabrol (**14**).^15^ The similar NMR data between the subunit of **1** and lonfuranacid A, along with the coupling constant between H-9′ and 10′ (*J*_9′,10′_ = 4.5 Hz), supported the relative configurations of C-9′ and C-10′ as being the same as in lonfuranacid A. Thus, the structure of **1** was determined as lonfuranacid A-4-*O*-carabrol and named carabrolate A.

Compound **2** exhibited the same molecular formula as **1** by (+)-HRESIMS analysis at *m*/*z* 477.2838. The IR and NMR spectra of **2** were very similar to those of **1**, indicating that **2** was also a carabrol derivative. A comparison of the ^13^C NMR data (Table 1) of **2** with those of **1** revealed that these compounds were nearly identical, except that the resonances of C-8′, C-9′, and C-10′ of **2** shifted downfield in a range of 2–5 ppm compared with those of **1**, which could be due to the configurational change at C-9′ in the furanoctanoic acid unit of **2**. The *J*_9′,10′_ coupling constant of 6.7 Hz supported that H-9′ and H-10′ were opposite, the same as in lonfuranacid B.^20^ Therefore, the structure of **2** was determined as lonfuranacid B-4-*O*-carabrol, and it was named carabrolate B.

Compound **3** was purified as a yellow oil. Its molecular formula (C_24_H_32_O_5_) was inferred by (+)-the HRESIMS analysis at *m*/*z* 423.2135 [M + Na]^+^ (calcd for 423.2142). The ^1^H and ^13^C NMR data (Tables 1 and 2) of **3** identified it as a carboxylic acid ester of carabrol at C-4. The ^1^H–^1^H correlations of H-2′/H-3′/H-8′/H-7′ and HMBC correlations from H-2′ to C-1′, H-3′ to C-1′, C-5′, and C-7′, H-9′ to C-3′ and C-5′, H-7′ and H-8′ to C-6′, and H-5′ to C-6′ and C-7′ (Figure 2) established the acid unit of **3** as 2-methyl-4-oxo-2-cyclohexene-1-acetic acid, an oxidation product of limonene.^21^ The key correlation between H-4 (δ_H_ 4.96) and C-1′ (δ_C_ 171.5) confirmed that the acid unit is attached to C-4. Thus, the structure of **3** was determined as shown and named carabrolate C.

Compound **4** was isolated as a yellow oil. HRESIMS analysis of **4** gave a [M + H]^+^ peak at *m*/*z* 399.216 (calcd for 399.2166), indicating the molecular formula C_24_H_30_O_5_, with 10 degrees of unsaturation. The presence of a carabrol group in **4** was supported by an analysis of ^1^H and ^13^C NMR data (Tables 1 and 2). In addition to resonances for carabrol, an ABX aromatic system δ_H_ 6.61 (dd, *J* = 8.2, 2.7 Hz, H-7′), 6.65 (d, *J* = 2.7 Hz, H-5′), and 7.04 (d, *J* = 8.2 Hz, H-8′) and six aromatic carbons (δ_C_ 112.8, 117.2, 125.3, 131.3, 138.4, and 154.6) were observed in the ^1^H and ^13^C NMR spectra of **4**. The HMBC correlations from H-2′ to C-1′, C-4′, and C-8′, H-9′ to C-3′ and C-5′, and H-4 to C-1′ (Figure 2) were further indicative of a 2-methyl-4-hydroxyphenylacetic acid^22^ unit in **4**, attached to C-4 of the carabraol group. Compared to those of **3**, chemical shifts of H-1–6, H-9, H-14, and H-15 of **4** were upfield in a range of 0.04–0.33 ppm, which could be caused by the shielding effect, inductive effect, and spatial proximity of the 2-methyl-4-hydroxyphenylacetic acid moiety in **4**. Thus, the structure of **4** was determined as shown and named carabrolate D.

**Figure 3:**
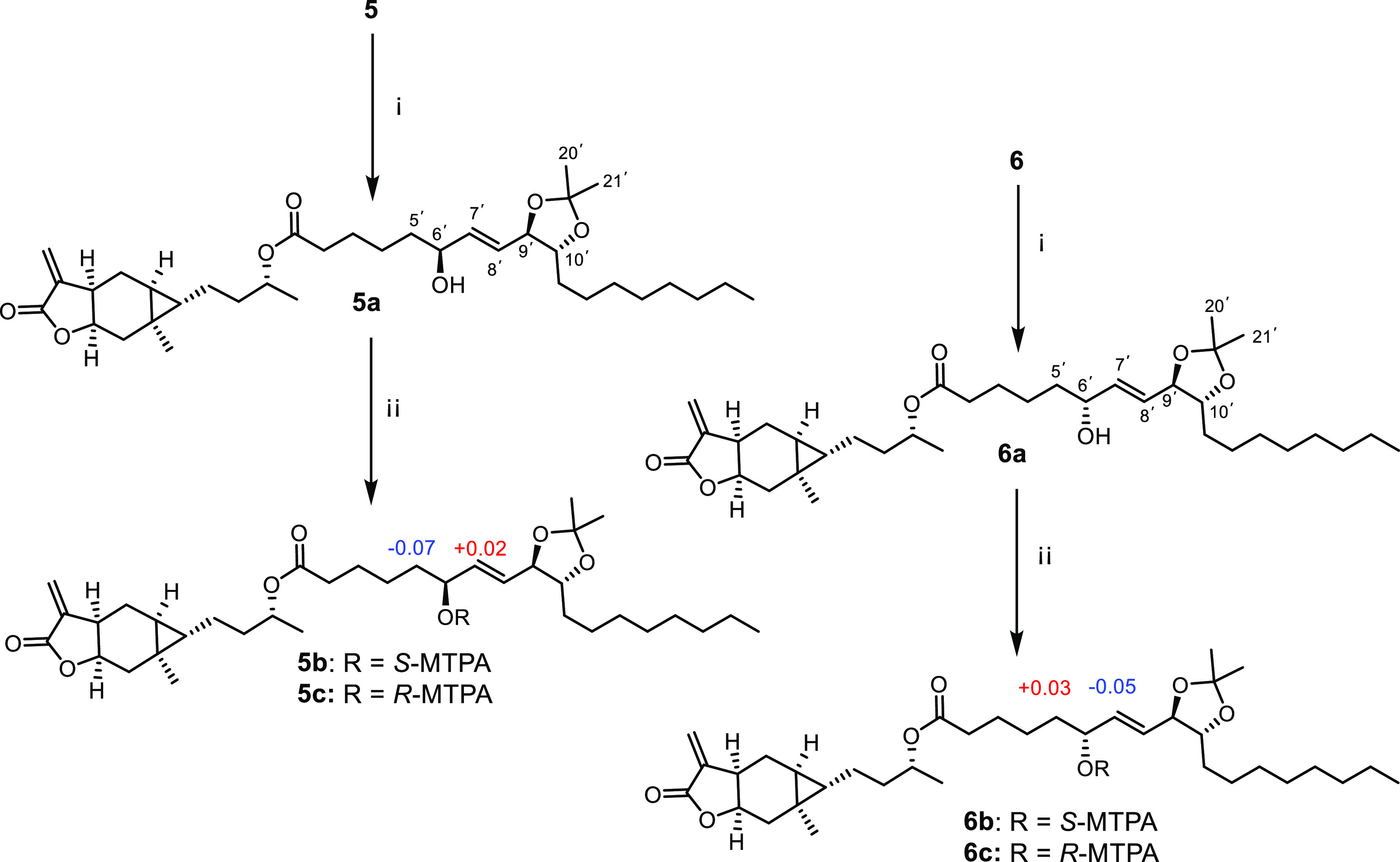
Ketalization and Mosher esterification for compounds **5** and **6**. Δδ_*S–R*_ values for Mosher esters (**5b**/**5c** and **6b**/**6c**) are shown. Reagents and conditions: (i) Acetone, *p*-toluene sulfonic acid (PDSA), 37 °C, 2 h; (ii) DMAP, (*S*)- or (*R*)-MTPA chloride, room temperature, 2 h.

Carabrolates E (**5**) and F (**6**) presented the same molecular formula C_33_H_54_O_7_ based on analysis of the HRESIMS and NMR data. The ^1^H and ^13^C NMR data (Tables 1–3) of **5** and **6** were similar, revealing that these compounds are carabraol-4-*O*-fatty acid esters. In the ^1^H NMR spectrum of **5**, resonances assigned to an oxylipin group were observed, including a triplet methyl group at δ_H_ 0.89 (t, *J* = 6.9 Hz, H-18′), 11 methylenes at δ_H_ 1.29–2.26, two olefinic protons at δ_H_ 5.79 (dd, *J* = 15.5, 6.5 Hz, H-7′) and 5.68 (dd, *J* = 15.5, 6.2 Hz, H-8′), and three oxygenated methines at δ_H_ 4.13 (q, *J* = 6.5 Hz, H-6′), 3.93 (t, *J* = 6.2 Hz, H-9′), and 3.46 (ddd, *J* = 9.1, 6.2, 2.8 Hz, H-10′). The oxylipin group in **5** exhibited almost identical NMR data to those of a natural 6,9,10-trihydroxyoctadec-7-enoic acid,^23^ which were confirmed by 2D NMR analysis (Figure 2). The coupling constant of H-9′, 10′ (*J*_9′,10′_ = 6.2 Hz) and identical NMR data to those of 9*R*,10*R*-trihydroxyoctadec-7-enoic acid^23^ deduced the 9′β-OH, and 10′β-OH in **5**. Thus, the structure of **5** was determined as trihydroxyoctadec-7-enate. 1D and 2D NMR data analysis revealed that **5** and **6** have the same planar structure. The major differences between them were the chemical shifts of C-5′ to C-8′ and the NOE correlation of H-6′/H-8′ in **5** but not in **6**, which suggested that the hydroxy group at the C-6′ in **6** was opposite to that of **5**.^24^ To establish the absolute configurations of the C-6′, C-9′, and C-10′ in **5** and **6**, vicinal diols in **5** and **6** were first protected by ketals to give **5a** and **6a** (Figure 3) according to the previously described procedure.^25^ The secondary alcohol at C-6′ in **5a** and **6a** was then derivatized with (*S*)- and (*R*)-α-methoxy-α-(trifluoromethyl)phenylacetyl (MTPA) chlorides,^26,27^ yielding their *S*- and *R*-MTPA esters (**5b**/**5c**, **6b**/**6c**) (Figure 3). By analysis of Δδ_*S–R*_ values of the ^1^H NMR chemical shift between **5b** and **5c** and between **6b** and **6c**, respectively, the absolute configurations of the C-6′ in **5** and **6** were designated as *S* and *R*, respectively (Figure 3). Based on the relative configuration of C-9′ and C-10′, the absolute configurations of oxylipin moieties in **5** and **6** were determined as 6′*S*,9′*R*,10′*R* and 6′*R*,9′*R*,10′*R*, respectively.

**Table 3:** ^1^H NMR (600 MHz) Data of **6**–**11** (CDCl_3_)

no.	**6**	**7**	**8**	**9**	**10**	**11**
1	0.44, td (7.3, 4.1)	0.44, td (7.2, 4.1)	0.44, td (7.2, 4.1)	0.44, td (7.2,4.1)	0.44, td (7.2, 4.1)	0.44, td (7.2, 4.1)
2	1.32, m	1.31, m	1.32, m,	1.29, m	1.31, m	1.30, m
	1.63, m	1.61, m	1.60, m	1.62, m	1.63, m	1.62, m
3	1.56, m	1.57, m	1.56, m	1.59, m	1.56, m	1.55, m
	1.68, m	1.69, m	1.68, m	1.72, m	1.68, m	1.67, m
4	4.92, q (6.3)	4.91, q (6.3)	4.91, q (6.3)	4.96, dt (5.9	4.91, h (6.3)	4.91, dq (12.1, 6.3)
5	0.34, ddd (8.7, 7.0, 4.1)	0.34, ddd (8.8, 7.1, 4.1)	0.34, ddd (8.8, 7.1, 4.1)	0.34, ddd (8.8, 7.0, 4.1	0.34, ddd (8.9, 7.1, 4.1)	0.34, ddd (8.7, 7.0, 4.1)
6	0.91, m	0.91, m	0.93, m	0.91, m	0.92, m	0.93, m
	2.35, m	2.36, m	2.35, m	2.36, m	2.35, m	2.36, dd (14.3,7.1)
7	3.15, m	3.15, m	3.25, m 3.15	3.15, m	3.16, m	3.15, m
8	4.78, ddd (11.5, 8.8, 6.1)	4.78, ddd (11.5, 8.8, 6.1)	4.78, ddd (11.5, 8.8, 6.1)	4.78, ddd (11.4, 8.8, 6.1)	4.78, ddd (11.5, 8.8, 6.1)	4.78, ddd (11.5, 8.8, 6.1)
9	0.96, m	0.96, m	0.95, m	0.96, m	0.95, m	0.96, dd (13.8, 11.5)
	2.32, m	2.32, m	2.32, m	2.32, m	2.32, m	2.32, dd (13.8,6.2)
13	5.55, d (2.5)	5.55, d (2.5)	5.55, d (2.5)	5.55, d (2.5)	5.55, d (2.50)	5.55, d (2.5)
	6.24, d (2.5)	6.24, d (2.5)	6.24, d (2.5)	6.24, d (2.5)	6.24, d (2.50)	6.24, d (2.5)
14	1.06, s	1.06, s	1.06, s	1.07, s	1.06, s	1.06, s
15	1.21, d (6.2)	1.21, d (6.2)	1.21, d (6.2)	1.23, d (6.3)	1.21, d (6.2)	1.21, d (6.3)
2′	2.25, m	2.26, t (7.6)	2.26, t (7.6)	2.38, dd (16.3,9.1)	2.26, t (7.5)	2.26, t (7.5)
				2.47, dd (16.3,3.1)		
3′	1.62, m	1.61, m	1.61, m	3.99, tt (7.8, 4.4)	1.31, m	1.61, m
4′	1.62, m	1.30, m	1.31 m	1.43, m	1.31, m	1.30, m
				1.52, m		
5′	1.54, m	1.30, m	1.31 m	1.29, m	1.31, m	1.30, m
6′	4.16, q (6.2)	1.30, m	1.31 m	1.25, m	1.31, m	1.30, m
7′	5.83, dd (15.5, 6.0, 1.1)	1.30, m	1.31 m	1.25, m	1.61, m	1.30, m
8′	5.71, dd (15.5, 6.4, 1.2)	1.30, m	1.57, m	1.25, m	2.32, t (7.5)	2.01, q (6.7)
9′	3.94, t (6.4)	1.43, m	3.63, t (6.6)	1.25, m		5.34, m
10′	3.46, ddd (9.3, 6.4, 3.2)	3.58, dq (8.4, 5.1)		1.25, m		5.35, m
11′	1.41, m 1.50, m	1.43, m		1.25, m		2.02, q (6.7)
12′	1.63, m	1.32, m		1.25, m		1.30, m
13′	1.31, m	1.32, m		1.25, m		1.30, m
14′	1.31, m	1.32, m		1.25, m		1.30, m
15′	1.31, m	1.57, m		1.25, m		1.30, m
16′	1.29, m	3.65, t (6.6)		1.25, m		1.26, m
17′	1.30, m			0.89, t (7.0)		1.29, m
18′	0.89, t (6.8)					0.88, t (7.0)

**Figure 4:**
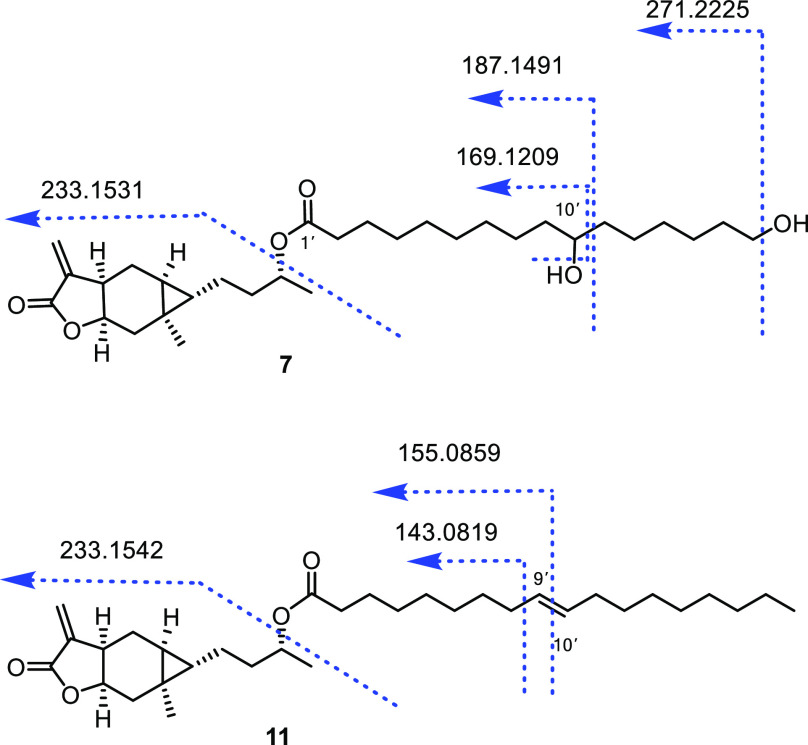
Some diagnostic HRESIMS/MS (+) fragment ions of **7** and **11**.

**Figure 5:**
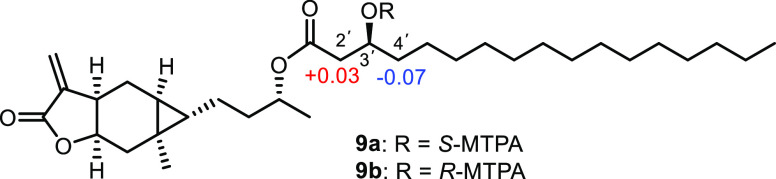
Δδ_S–R_ values for Mosher esters **9a** and **9b**.

Compounds **7**–**9** were obtained as a yellow oil, with the molecular formulas C_31_H_52_O_6_, C_24_H_38_O_5_, and C_32_H_54_O_5_, determined by analysis of the corresponding HRESIMS ions [M + Na]^+^ at *m*/*z* 543.3656, 429.2608, and 541.3863, respectively. Compounds **7**–**9** are also esters of carabrol composed of fatty acids, as indicated by the analysis of NMR data (Tables 1 and 3), except for varying degrees of oxidation. The fatty acid in **7** was established as 10,16-dihydroxy hexadecanoic acid by the observation of an oxygenated methylene at δ_H_ 3.65 (t, *J* = 6.6 Hz, H-16′), an oxygenated methine δ_H_ 3.58 (dq, *J* = 8.4, 5.1 Hz, H-10′), and 13 methylenes at δ_H_ 1.30–2.26 (m), as well as 16 carbon signals including 14 methylenes (an oxygenated methylene at δ_C_ 63.0, C-16′), an oxygenated methine at δ_C_ 72.0, C-10, and a quaternary carbon at δ_C_ 173.5 (C-1′) in the ^1^H and ^13^C NMR spectra of **7**, which were consistent with the data of 10,16-dihydroxy hexadecanoic acid.^28^ The position of the hydroxy group at C-10′ in **7** was further confirmed by characteristic fragment ions at *m*/*z* 271.2225, 187.1491, and 169.1209 in its HRESIMS/MS spectrum (Figure 4). By comparison with known spectroscopic data, the fatty acid groups of **8** and **9** were verified to be 9-hydroxynonanoic acid^29^ and 3-hydroxyheptadecanoic acid, respectively.^30^ The HMBC correlations from H-9′ to C-7′/C-8′ in **8** and from H-3′ to C-1′/C-2′/4′ in **8** also supported the existence of 9′-OH in **8** and 3′-OH in **9** (Figure 2). The secondary alcohol at C-3′ in **9** was derivatized with (*S*)- and (*R*)-MTPA chloride, yielding its *S*- and *R*-MTPA esters (**9a** and **9b**) (Figure 5). The absolute configuration of C-3′ in **9** was determined as *S* based on the Δδ_*S–R*_ values of its Mosher esters (**9a** and **9b**). Moreover, for compounds **7**–**9**, fatty acid groups attaching to C-4 of carabrol were established by their key HMBC correlations between H-4 (δ_H_ 4.91–4.96) and C-1′ (δ_C_ 172.7–173.5) (Figure 2). Hence, the structures of **7**–**9** were determined as shown and named carabrolates G (**7**), H (**8**), and I (**9**).

The molecular formula C_24_H_36_O_6_ of **10** was established by analysis of (+)-HRESIMS at *m*/*z* 421.2577 [M + H]^+^ (calcd for 421.2585). The ^1^H and ^13^C NMR spectroscopic data (Tables 1 and 3) of **10** showed the presence of the carabrol moiety. Moreover, the resonances of an azelaic acid group in **10** were revealed by the seven methylenes (δ_H_ 1.31–2.32 and δ_C_ 24.7–34.7) and two carbonyl carbons (δ_C_ 173.5, C-1′ and 179.0, C-9′) in the ^1^H and ^13^C NMR spectra. Furthermore, these data were identical to those of the natural azelaic acid.^31^ The HMBC cross-peak between H-4 (δ_H_ 4.91) and C-1′ (δ_C_ 173.5) proved the connection of the carabranolide moiety and azelaic acid (Figure 2). Thus, the structure of **10** was determined as shown and named carabrolate J.

Compound **11**, a yellow oil, presented the molecular formula C_33_H_54_O_4_ based on analysis of (+)-HRESIMS at *m*/*z* 537.391 [M + H]^+^ (calcd for 537.3914). Its ^1^H and ^13^C NMR spectroscopic data (Tables 1 and 3) were extremely close to those of carabrol-4-*O*-linoleate (**12**)^17^ in CDCl_3_, except for the absence of signals for a double-bond group at C-12′ and C-13′. Compound **11** was comprised of a carabrol unit and oleic acid group through HRMS and NMR data analysis.^32^ The position of the double bond at C-9′ and C-10′ in **11** was verified by fragment ions at *m*/*z* 155.0859 and 143.0819 through HRESIMS/MS analysis (Figure 4). Moreover, the oleic acid group attaching to C-4 of carabrol was defined by the key HMBC cross-peak (Figure 2) between H-4/C-1′. Therefore, compound **11** was established as carabrolate K.

Given the anti-inflammatory activity of carabrol,^33^ the activity of carabrol derivatives (**1**–**16**) on LPS-induced NO production in RAW264.7 cells was investigated. As shown in Table 4, compounds **1**–**8**, **9**, and **14**–**16** exhibited notable inhibitory effects on LPS-induced NO release in RAW264.7 cells, with IC_50_ values ranging from 5.8 to 15.1 μM. All tested compounds had no obvious effect on cell viability at a concentration of 20 μM. Structurally, the principal distinction among compounds **1**–**16** is the substituents at the C-4 position. The unsubstituted carabrol (**14**) exhibited considerable inhibitory activity against NO production with an IC_50_ value of 10.96 μM. When the 4-OH of carabrol was oxidized (**15**), acetylated (**16**), or esterified with 2-methyl-4-hydroxyphenylacetic acid (**4**), the IC_50_ values of **4** (5.82 μM), **15** (6.77 μM), and **16** (5.64 μM) decreased almost one-fold compared with carabrol. In contrast, the esterification of carabrol with the long-chain fatty acids diminished the bioactivity, while cyclic or unsaturated and oxidized fatty acid esters of carabrol (**2**, **3**, **5**–**8**, **10**) exhibited a similar activity to that of carabrol (Table 4).

**Table 4:** IC_50_ Values of **1**–**16** for NO Inhibitory Activity

no.	IC_50_ (μM)	no.	IC_50_ (μM)
**1**	15.06 ± 1.01	**9**	>20
**2**	8.67 ± 0.74	**10**	9.35 ± 0.78
**3**	9.15 ± 0.96	**11**	>20
**4**	5.82 ± 0.43	**12**	>20
**5**	11.80 ± 0.82	**13**	>20
**6**	11.56 ± 0.67	**14**	10.96 ± 0.43
**7**	9.87 ± 0.75	**15**	6.77 ± 0.57
**8**	9.75 ± 0.54	**16**	5.64 ± 0.32
Dex	8.52 ± 0.61		

**Figure 6:**
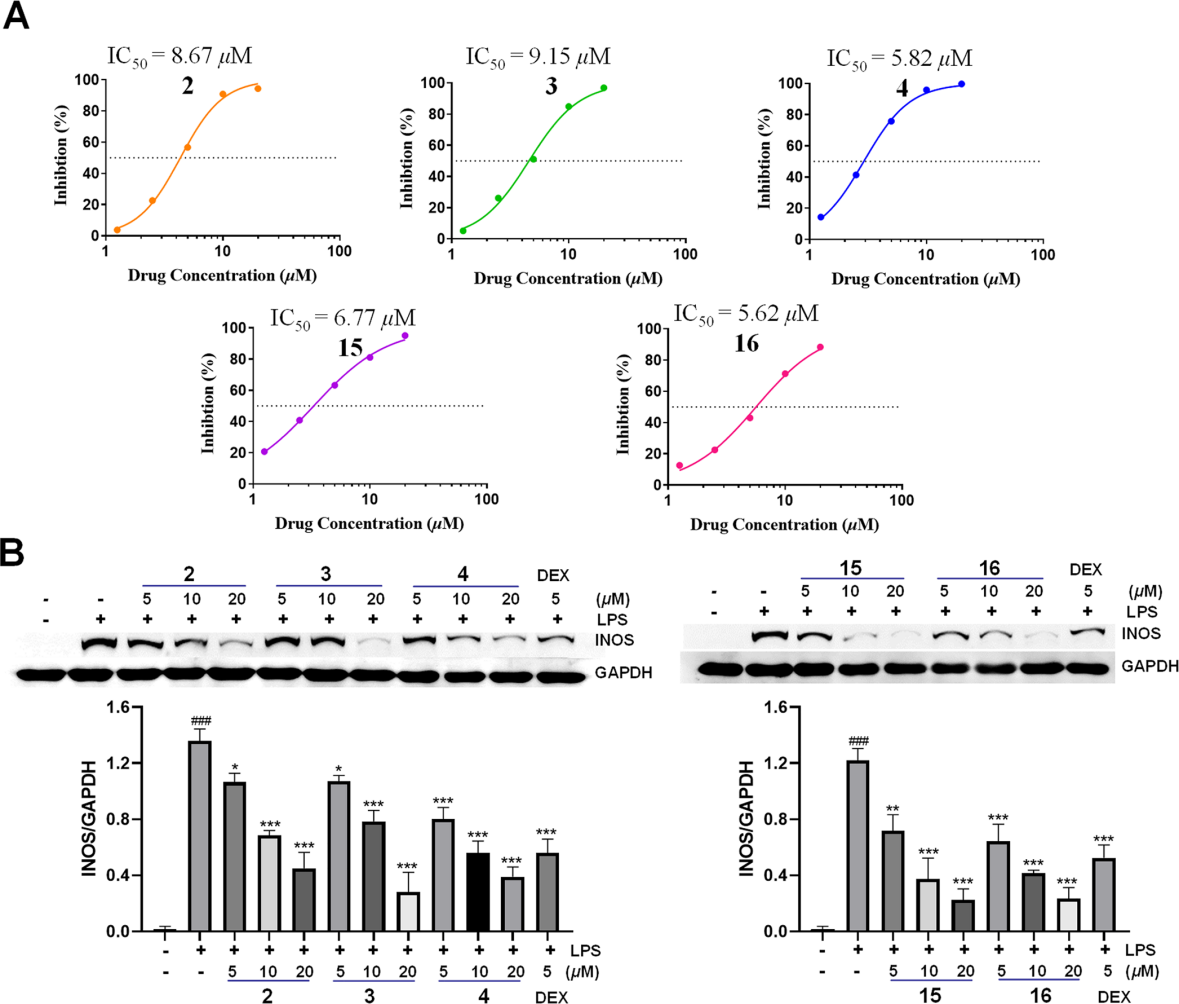
Anti-inflammatory activity of tested compounds on LPS-induced RAW264.7 cells. (A) Compounds **2**–**4**, **15**, and **16** inhibited LPS-induced NO production in a concentration-dependent manner. (B) Compounds **2**–**4**, **15**, and **16** dose-dependently decreased LPS-induced iNOS expression in RAW264.7 cells. Dexamethasone (DEX) was used as a positive control. (Data are presented as mean ± SD, ^###^*p* < 0.001, compared with the blank control group; *p < 0.05, **p < 0.01, and ***p < 0.001, compared with the LPS group.)

Inducible nitric oxide synthase (iNOS) is a key enzyme for LPS-induced NO release, and the effects of selected compounds (**2**–**4**, **15**, and **16**) on LPS-stimulated iNOS protein expression were then evaluated in RAW264.7 cells. As a result, compounds **2**–**4**, **15**, and **16** dose-dependently inhibited LPS-induced iNOS expression at 5, 10, and 20 μM (Figure 6). These results suggested that carabrol derivatives are anti-inflammatory agents.

## Experimental Section

### General Experimental Procedures

Optical rotations were defined by an Autopol I polarimeter (Rudolph, Flanders, USA). UV and ECD spectra were recorded with a J-1500 circular dichroism spectrometer (JASCO, Japan). IR data were measured with an IR Affinity-1S spectrometer (Shimadzu, Japan). NMR experiments were recorded with a Bruker Ascend-600 spectrometer (Bruker, Germany) with TMS used as an internal standard. An Agilent-6230 Q-TOF mass spectrometer with an Agilent 1260 UHPLC system was used to obtain HRESIMS data (Agilent, USA). HRESIMS/MS analyses for **7** and **11** were performed on an Agilent-6230 Q-TOF with the energy variable from 10 to 40 eV. Medium-pressure liquid chromatography (MPLC) was performed on a Buchi C-620 system (Buchi, Switzerland) equipped with a Siliabond C_18_ column (ODS gel, 5 μm, 49 × 460 mm) with a flow rate of 100 mL/min. Semipreparative HPLC was performed on an Agilent 1260 HPLC system (Agilent, USA) coupled with a DAD detector by using an XBrdige C_18_ column (5 μm, 10 × 250 mm, Waters, USA) at a flow rate of 3 mL/min. Silica gel (200–300 mesh, Qingdao Haiyang Company, People’s Republic of China) was used for column chromatography (CC). The analysis by thin-layer chromatography (TLC) was carried out by silica gel GF254 (Merck, Darmstadt, Germany), and potassium bismuth iodide was used as a visualizing reagent.

Analytical grade petroleum ether (PE), ethyl acetate (EtOAc), and methanol (MeOH) and HPLC grade MeOH and acetonitrile (MeCN) were purchased from Anaqua Global International Inc. (Cleveland, OH, USA). CDCl_3_ containing TMS (1%) was purchased from Cambridge Isotope Laboratories, Inc. (Andover, MA, USA). Deionized water was obtained using a Millipore Milli-Q-plus system (Millipore, Bedford, MA, USA).

The primary antibody against iNOS (CST no. 13120S) was purchased from Cell Signaling Technology (Beverly, MA, USA). The primary antibody against GAPDH (cat. no. 60004-1-Ig) was obtained from Proteintech Group (Chicago, IL, USA). Secondary antibodies, goat anti-mouse (cat. no. 926-32210) and goat anti-rabbit (cat. no. 926-32211) IgG, were bought from LI-COR 800CW (Lincoln, NE, USA). The anti-inflammatory agent dexamethasone (lot no. 923H058) was purchased from Solarbio (China). LPS was purchased from Sigma-Aldrich (St. Louis, MO, USA).

### Plant Material

The fruits of *C. abrotanoides* were purchased in May 2022 from the Bozhou medicinal materials market in Anhui Province, People’s Republic of China, and identified by Dr. Guo-yuan Zhu from Macau University of Science and Technology. The voucher specimen (CA-2022-05) is already stored in the State Key Laboratory of Quality Research in Chinese Medicine, Macau University of Science and Technology.

### Extraction and Isolation

The dried fruits of *C. abrotanoides* (15 kg) were powdered and extracted with 80% EtOH at room temperature. The extract was concentrated under reduced pressure to afford 1.6 L of the concentrated extract, which was then partitioned successively with PE and EtOAc. The ^1^H and ^13^C NMR spectra of PE and EtOAc extracts demonstrated the characteristic signals indicative of carabranolide derivatives, which were used to guide the further isolation of targeted compounds.

The PE extract (241g) was separated by silica gel CC (PE–EtOAc–MeOH, 1:0:0 to 0:1:1), and the eluates were analyzed by TLC and combined to give 6 fractions. NMR analyses of these fractions showed that fraction P.3 contained carabranolide derivatives. Fraction P.3 (26 g) was then subjected to preparative MPLC (MeCN–H_2_O, 40:60 to 100:0) to give 9 subfractions (P.3.1–9). Fraction P.3.6 (2.4 g) was further fractionated by MPLC (MeCN–H_2_O, 85:15) to yield 4 subfractions (P.3.6.1–4). Fraction P.3.6.2 was purified by semipreparative HPLC (MeCN–H_2_O, 90:10) to afford **12** (*t*_R_ 18 min, 12 mg). Afterward, compounds **11** (*t*_R_ = 15 min, 16 mg) and **13** (*t*_R_ = 32 min, 28 mg) were purified by HPLC (MeOH–H_2_O, 90:10) from fraction P.3.6.3.

The EtOAc extract (678 g) was fractionated by silica gel CC (PE–EtOAc–MeOH, 1:0:0 to 0:1:1). A total of 50 fractions (1000 mL/each) were collected. After TLC analysis, the fractions were combined into 9 fractions. Carabrol derivatives were found in fractions E.6 and E.9 by NMR analysis. Fraction E.6 (98 g) was further separated into 10 fractions (E.6.1–10) by MPLC with a gradient mobile phase of MeCN–H_2_O (30:100 to 100:0). Fraction E.6.8 was separated by HPLC with MeCN and H_2_O (78:22) as eluent to yield 8 subfractions (E.6.8.1–8). Fraction E.6.8.2 was purified by preparative HPLC eluted with MeCN–H_2_O (80:20) to give **3** (*t*_R_ 23 min, 6.2 mg). Compounds **8** (*t*_R_ 28 min, 3.1 mg) and **10** (*t*_R_ 31 min, 2.8 mg) were obtained from fraction E.6.8.8 via semipreparative HPLC eluted with a MeCN–H_2_O mobile phase (90:10). Fraction E.7 (50 g) was subjected to MPLC eluted with MeCN–H_2_O (30:100 → 100:0), giving 11 fractions (E.7.1–11). Fraction E.7.4 (2.5 g) was separated again into 9 subfractions (E.7.4.1–9) by MPLC with 70% MeCN. Compound **9** (*t*_R_ 30 min, 2.2 mg) was obtained by HPLC eluted with 95% ACN from fraction E.7.4.4. Fraction E.7.4.5 was purified by semipreparative HPLC (MeOH–H_2_O, 60:40) to give **15** (*t*_R_ 12 min, 11 mg) and **14** (*t*_R_ 18 min, 3.5 mg). Fraction E.7.5 was separated by MPLC (MeCN–H_2_O, 70:30 → 100:0) to give 11 fractions (E.7.1–11). Fraction E.7.5.5 was applied to semipreparative HPLC (MeOH–H_2_O, 61:39) to yield **16** (*t*_R_ 21 min, 3.6 mg). Moreover, compound **4** (*t*_R_ 28 min, 1.1 mg) was obtained from Fr.E.7.5.6. via semipreparative HPLC eluted with 75% MeCN. Fraction E.7.7 (3.3 g) was separated by HPLC (MeCN–H_2_O, 60:40) to yield 12 fractions (E.7.7.1–12). Fraction E.7.7.12 was purified by semipreparative HPLC eluted with 82% MeOH to give **7** (*t*_R_ 21 min, 3.2 mg). Fraction E.9 (95 g) was separated by MPLC (MeCN–H_2_O 70:100 to 100:0) to obtain 8 fractions (E.9.1–8). Fraction E.9.4 (4.5 g) was subjected to MPLC eluted with isocratic MeCN–H_2_O (80:20) to get 11 fractions (E.9.4.1–11). Subsequently, fraction E.9.4.9 (79 mg) was further purified by semipreparative HPLC (MeOH–H_2_O, 85:15) to give **1** (*t*_R_ 31 min, 3.8 mg) and **2** (*t*_R_ 39 min, 3.2 mg). Fraction E.9.4.11 was fractionated using HPLC eluted with 89% MeOH to get 6 fractions (E.9.4.11.1–6). Subsequently, compounds **5** (*t*_R_ 31 min, 1.2 mg) and **6** (*t*_R_ 39 min, 2.9 mg) were isolated via semipreparative HPLC eluted with 85% MeOH from fraction E.9.4.11.4.

#### Carabrolate A (**1**)

Yellow oil; [α]_D_^25^ +12.6 (*c* 0.5, MeOH); IR (KBr) ν_max_ 3487, 2940, 2862, 2353, 1759, 1543, 1450, 1258, and 1188 cm^–1^; UV (MeOH) λ_max_ (log ε) 195 (3.80) nm; ^1^H and ^13^C NMR data, see Tables 1 and 2; HRESIMS *m*/*z* 477.2833 [M + H]^+^ (calcd for C_27_H_40_O_7_, 477.2847).

#### Carabrolate B (**2**)

Yellow oil; [α]_D_^25^ +21.9 (*c* 0.5, MeOH); IR (KBr) ν_max_ 3487, 2932, 2862, 2361, 2338, 1759, 1366, 1265, and 1180 cm^–1^; UV (MeOH) λ_max_ (log ε) 195 (4.00) nm; ^1^H and ^13^C NMR data, see Tables 1 and 2; HRESIMS *m*/*z* 477.2838 [M + H]^+^ (calcd for C_27_H_40_O_7_, 477.2847).

#### Carabrolate C (**3**)

Yellow oil; [α]_D_^25^ +48.2 (*c* 0.5, MeOH); IR (KBr) ν_max_ 2940, 2870, 2361, 2338, 1728, 1667, 1265, 1180, and 1150 cm^–1^; UV (MeOH) λ_max_ (log ε) 229 (4.05) nm; ^1^H and ^13^C NMR data, see Tables 1 and 2; HRESIMS *m*/*z* 423.2135 [M + Na]^+^ (calcd for C_24_H_32_O_5_, 423.2142).

#### Carabrolate D (**4**)

Yellow oil; [α]_D_^25^ +25.4 (*c* 0.5, MeOH); IR (KBr) ν_max_ 3726, 2940, 2870, 2361, 2338, 1736, 1667, 1505, 1458, 1265, 1227, 1157, and 995 cm^–1^; UV (MeOH) λ_max_ (log ε) 197 (4.21) nm; ^1^H and ^13^C NMR data, see Tables 1 and 2; HRESIMS *m*/*z* 399.216 [M + H]^+^ (calcd for C_24_H_30_O_5_, 399.2166).

#### Carabrolate E (**5**)

Yellow oil; [α]_D_^25^ +17.1 (*c* 0.5, MeOH); IR (KBr) ν_max_ 3688, 3372, 2932, 2862, 1728, 1458, 1373, 1350, 1265, 1188, and 1150 cm^–1^; UV (MeOH) λ_max_ (log ε) 195 (3.90) nm; ^1^H and ^13^C NMR data, see Tables 1 and 2; HRESIMS *m*/*z* 580.4204 [M + NH_4_]^+^ (calcd for C_33_H_54_O_7_, 580.4208).

#### Carabrolate F (**6**)

Yellow oil; [α]_D_^25^ +26.60 (*c* 0.5, MeOH); IR (KBr) ν_max_ 3726, 3402, 2932, 2862, 2361, 1759, 1728, 1667, 1458, 1373, 1265, 1188, 1142, 1103, 1042, and 988 cm^–1^; UV (MeOH) λ_max_ (log ε) 195 (3.92) nm; ^1^H and ^13^C NMR data, see Tables 2 and 3; HRESIMS *m*/*z* 580.4202 [M + NH_4_]^+^ (calcd for C_33_H_54_O_7_, 580.4208).

#### Carabrolate G (**7**)

Yellow oil; [α]_D_^25^ +23.5 (*c* 0.5, MeOH); IR (KBr) ν_max_ 3626, 2931.80 2862.36 2360.87 2338, 1759, 1728, 1458, 1373, 1265, 1180, and 995 cm^–1^; UV (MeOH) λ_max_ (log ε) 195 (4.14) nm; ^1^H and ^13^C NMR data, see Tables 2 and 3; HRESIMS *m*/*z* 543.3656 [M + Na]^+^ (calcd for C_31_H_52_O_4_, 543.36525); (+)-HRESIMS/MS: 233.1531 [C_15_H_21_O_2_]^+^, 271.2225 [C_16_H_31_O_3_]^+^, 187.1491 [C_10_H_19_O_3_]^+^, and 169.1209 [C_10_H_17_O_2_]^+^.

#### Carabrolate H (**8**)

Yellow oil; [α]_D_^25^ +22.0 (*c* 0.5, MeOH); IR (KBr) ν_max_ 3726, 2932, 2862, 2361, 1728, 1373, 1188, and 995 cm^–1^; UV (MeOH) λ_max_ (log ε) 195 (3.83) nm; ^1^H and ^13^C NMR data, see Tables 2 and 3; HRESIMS *m*/*z* 429.2608 [M + Na]^+^ (calcd for C_24_H_38_O_5_, 429.2611).

#### Carabrolate I (**9**)

Yellow oil; [α]_D_^25^ +23.5 (*c* 0.5, MeOH); IR (KBr) ν_max_ 3726, 2924, 2855, 2361, 2338, 1759, 1728, 1265, 1180, 1150, and 995 cm^–1^; UV (MeOH) λ_max_ (log ε) 211 (3.80) nm; ^1^H and ^13^C NMR data, see Tables 2 and 3; HRESIMS *m*/*z* 541.3854 [M + Na]^+^ (calcd for C_32_H_54_O_5_, 541.3863).

#### Carabrolate J (**10**)

Yellow oil; [α]_D_^25^ +20.0 (*c* 0.5, MeOH); IR (KBr) ν_max_ 3726, 2932, 2862, 2361, 2338, 1759, 1728, 1458, 1265, 1180, 1150, 1096, 995, and 964 cm^–1^; UV (MeOH) λ_max_ (log ε) 212 (4.17) nm; ^1^H and ^13^C NMR data, see Tables 2 and 3; HRESIMS *m*/*z* 421.2577 [M + H]^+^ (calcd for C_24_H_36_O_6_, 421.2585).

#### Carabrolate K (**11**)

Yellow oil; [α]_D_^25^ +12.8 (*c* 0.5, MeOH); IR (KBr) ν_max_ 2361, 2338, 1728, 1659, 1188, and 995 cm^–1^; UV (MeOH) λ_max_ (log ε) 195 (3.82) nm; ^1^H and ^13^C NMR data, see Tables 2 and 3; HRESIMS *m*/*z* 537.3910 [M + Na]^+^ (calcd for C_33_H_54_O_4_, 537.3914). (+)-HRESIMS/MS: 233.1542 [C_15_H_21_O_2_]^+^, 155.0859 [C_9_H_15_O_2_]^+^, and 143.0819 [C_8_H_14_O_2_]^+^.

##### (*S*)-MTPA ester of **5a** (**5b**):

^1^HNMR (C_5_D_5_N, 600 MHz) δ_H_ 0.42 (1H, m, H-1), 1.29 (2H, m, H-2), 1.59 (1H, m, H-3a), 1.74 (1H, m, H-3b), 5.11 (1H, m, H-4), 0.24 (1H, m, H-5), 0.82 (1H, m, H-6a), 2.22 (1H, d, *J* = 13.2 Hz, H-6b), 3.06 (1H, m, H-7), 4.77 (1H, m, H-8), 0.94 (1H, m, H-9a), 2.21 (1H, m, H-9b), 5.51 (1H, br s, H-13a), 6.30 (1H, br s, H-13b), 0.96 (3H, s, H-14), 0.84 (3H, d, *J* = 6.2 Hz, H-15), 2.37 (2H, m, H-2′), 1.57–1.78 (4H, overlapped, H-3′ and 4′), 1.24 (2H, m, H-5′), 5.27 (1H, m, H-6′), 5.64 (1H, overlapped, H-7′), 5.68 (1H, overlapped, H-8′), 4.26 (1H, t, *J* = 6.1 Hz, H-9′), 3.84 (1H, m, H-10′), 1.17–1.34 (14H, overlapped, H-11′–17′), 0.83 (3H, t, *J* = 6.3 Hz, H-18′), 1.22 (3H, s, H-20′), 1.23 (3H, s, H-21′).

##### (*R*)-MTPA ester of **5a** (**5c**):

^1^HNMR (C_5_D_5_N, 600 MHz) δ_H_ 0.42 (1H, m, H-1), 1.30 (2H, m, H-2), 1.60 (1H, m, H-3a), 1.75 (1H, m, H-3b), 5.10 (1H, m, H-4), 0.24 (1H, m, H-5), 0.84 (1H, m, H-6a), 2.22 (1H, d, *J* = 13.2 Hz, H-6b), 3.05 (1H, m, H-7), 4.77 (1H, m, H-8), 0.92 (1H, m, H-9a), 2.19 (1H, m, H-9b), 5.51 (1H, d, *J* = 2.6 Hz, H-13a), 6.30 (1H, d, *J* = 2.6 Hz, H-13b), 0.97 (3H, s, H-14), 0.84 (3H, d, *J* = 6.5 Hz, H-15), 2.38 (2H, m, H-2′), 1.57–1.77 (4H, overlapped, H-3′ and 4′), 1.31 (2H, m, H-5′), 5.27 (1H, m, H-6′), 5.62 (1H, dd, *J* = 15.6, 6.1 Hz, H-7′), 5.64 (1H, dd, *J* = 15.6, 6.2 Hz, H-8′), 4.26 (1H, t, *J* = 6.2 Hz, H-9′), 3.85 (1H, m, H-10′), 1.19–1.33 (14H, overlapped, H-11′∼17′), 0.85 (3H, t, *J* = 6.7 Hz, H-18′), 1.19 (3H, s, H-20′), 1.20 (3H, s, H-21′).

##### (*S*)-MTPA ester of **6a** (**6b**):

^1^HNMR (C_5_D_5_N, 600 MHz) δ_H_ 0.42 (1H, td, *J* = 7.3, 3.8 Hz, H-1), 1.28 (2H, m, H-2), 1.59 (1H, m, H-3a), 1.74 (1H, m, H-3b), 5.11 (1H, m, H-4), 0.26 (1H, td, *J* = 7.9, 4.2 Hz, H-5), 0.81 (1H, m, H-6a), 2.20 (1H, d, *J* = 13.4 Hz, H-6b), 3.02 (1H, m, H-7), 4.78 (1H, ddd, *J* = 11.5, 8.7, 6.6 Hz, H-8), 0.96 (1H, m, H-9a), 2.18 (1H, dd, *J* = 13.1, 6.5 Hz, H-9b), 5.51 (1H, d, *J* = 2.5 Hz, H-13a), 6.30 (1H, d, *J* = 2.5 Hz, H-13b), 0.97 (3H, s, H-14), 0.84 (3H, d, *J* = 6.6 Hz, H-15), 2.38 (2H, m, H-2′), 1.57–1.74 (4H, overlapped, H-3′ and 4′), 1.73 (2H, m, H-5′), 5.73 (1H, q, *J* = 6.6 Hz, H-6′), 5.95 (1H, dd, *J* = 15.2, 6.6 Hz, H-7′), 5.98 (1H, dd, *J* = 15.2, 6.1 Hz, H-8′), 4.16 (1H, t, *J* = 6.1 Hz, H-9′), 3.80 (1H, m, H-10′), 1.19–1.38 (14H, overlapped, H-11′∼17′), 0.83 (3H, t, *J* = 7.8 Hz, H-18′), 1.24 (3H, s, H-20′), 1.26 (3H, s, H-21′).

##### (*R*)-MTPA ester of **6a** (**6c**):

^1^HNMR (C_5_D_5_N, 600 MHz) δ_H_ 0.41 (1H, td, *J* = 7.1, 3.6 Hz, H-1), 1.29 (2H, m, H-2), 1.56 (1H, m, H-3a), 1.73 (1H, m, H-3b), 5.11 (1H, m, H-4), 0.26 (1H, td, *J* = 7.7, 4.1 Hz, H-5), 0.80 (1H, m, H-6a), 2.18 (1H, d, *J* = 13.2 Hz, H-6b), 3.03 (1H, m, H-7), 4.76 (1H, m, H-8), 0.92 (1H, m, H-9a), 2.16 (1H, d, *J* = 13.0 Hz, H-9b), 5.51 (1H, s, H-13a), 6.30 (1H, s, H-13b), 0.96 (3H, s, H-14), 0.84 (3H, d, *J* = 6.4 Hz, H-15), 2.37 (2H, m, H-2′), 1.58–1.75 (4H, overlapped, H-3′ and 4′), 1.70 (2H, m, H-5′), 5.73 (1H, q, *J* = 6.8 Hz, H-6′), 6.00 (1H, dd, *J* = 15.1, 6.3 Hz, H-7′), 5.98 (1H, dd, *J* = 15.1, 6.8 Hz, H-8′), 4.16 (1H, t, *J* = 6.8 Hz, H-9′), 3.79 (1H, m, H-10′), 1.18–1.38 (14H, overlapped, H-11′∼17′), 0.83 (3H, t, *J* = 7.3 Hz, H-18′), 1.24 (3H, s, H-20′), 1.26 (3H, s, H-21′).

##### (*S*)-MTPA ester of **9** (**9a**):

^1^HNMR (C_5_D_5_N, 600 MHz) δ_H_ 0.43 (1H, td, *J* = 7.1, 3.9 Hz, H-1), 1.30 (2H, m, H-2), 1.63 (1H, m, H-3a), 1.76 (1H, m, H-3b), 5.11 (1H, dt, *J* = 12.7, 6.4 Hz, H-4), 0.28 (1H, td, *J* = 8.0, 4.2 Hz, H-5), 0.85 (1H, m, H-6a), 2.21 (1H, d, *J* = 13.1 Hz, H-6b), 3.01 (1H, m, H-7), 4.78 (1H, ddd, *J* = 11.6, 8.8, 6.2 Hz, H-8), 0.95 (1H, m, H-9a), 2.17 (1H, dd, *J* = 10.8, 5.0 Hz, H-9b), 5.51 (1H, d, *J* = 1.3 Hz, H-13a), 6.32 (1H, d, *J* = 1.3 Hz, H-13b), 0.98 (3H, s, H-14), 0.87 (3H, d, *J* = 5.8 Hz, H-15), 2.93 (2H, dd, *J* = 10.5, 3.0 Hz, H-2′), 5.87 (1H, m, H-3′), 1.75 (2H, m, H-4′), 1.22–1.31 (24H, overlapped, H-5′∼16′), 0.88 (3H, t, *J* = 7.3 Hz, H-17′).

##### (*R*)-MTPA ester of **9** (**9b**):

^1^HNMR (C_5_D_5_N, 600 MHz) δ_H_ 0.42 (1H, td, *J* = 7.2, 4.0 Hz, H-1), 1.32 (2H, m, H-2), 1.58 (1H, m, H-3a), 1.74 (1H, m, H-3b), 5.08 (1H, dt, *J* = 12.7, 6.4 Hz, H-4), 0.26 (1H, td, *J* = 8.0, 4.2 Hz, H-5), 0.84 (1H, m, H-6a), 2.22 (1H, d, *J* = 13.9 Hz, H-6b), 3.01 (1H, m, H-7), 4.78 (1H, ddd, *J* = 12.9, 8.8, 6.7 Hz, H-8), 0.96 (1H, m, H-9a), 2.17 (1H, dd, *J* = 12.8, 6.8 Hz, H-9b), 5.51 (1H, d, *J* = 2.1 Hz, H-13a), 6.31 (1H, d, *J* = 2.1 Hz, H-13b), 0.98 (3H, s, H-14), 0.87 (3H, d, *J* = 5.7 Hz, H-15), 2.90 (2H, dd, *J* = 10.0, 2.0 Hz, H-2′), 5.90 (1H, m, H-3′), 1.82 (2H, m, H-4′), 1.21–1.30 (24H, overlapped, H-5′∼16′), 0.87 (3H, t, *J* = 7.1 Hz, H-17′).

### Ketalization for Compounds **5** and **6**

Treatment of **5** and **6** (1.0 mg) with PDSA (30 μg) in 400 μL of acetone afforded the acetone ketal at 37 °C for 2 h. The reaction was monitored by UHPLC-QTOF-MS analysis. Then the mixtures were partitioned with CHCl_3_ and H_2_O to give **5a** and **6a**.

### Preparation of (*S*)- and (*R*)-MTPA Esters of **5a**, **6a**, and **9**

Compounds **5a**, **6a**, **and 9** (0.5 mg) were dissolved in 500 μL of deuterated pyridine. Then, 1.0 mg of 4-dimethylaminopyridine and 5 μL of (*S*)- or (*R*)-MTPA chlorides were added to the above solutions, which were allowed to react at room temperature for 2 h. The mixtures were subjected to an NMR spectrometer, and their ^1^H and ^1^H–^1^H COSY NMR spectra were recorded.

### Cell Lines and Cell Culture

The RAW264.7 cell line was obtained from ATCC (USA) and cultured in DMEM medium supplemented with 10% fetal bovine serum (FBS) (Gibco, UK) in a humidified atmosphere with 5% CO_2_ at 37 °C.

### Assay for NO Inhibitory Activity

NO inhibitory activity of selected compounds was evaluated by using an LPS-induced cell model. Briefly, RAW264.7 cells were inoculated into 96-well plates (1 × 10^5^ cells/well) and were treated with the test compounds or dexamethasone (DEX, a positive control) at concentrations of 1.25, 2.5, 5, 10, and 20 μM in triplicate. After being induced with LPS (100 ng/mL) for 24 h, the cell culture supernatants were collected to measure levels of NO by using the Griess reagent (Beyotime Biotechnology, People’s Republic of China) according to the manufacturer’s instructions. Finally, the absorbance at 550 nm was measured with a microplate reader. Concurrently, the MTT assay was employed to assess the viability of RAW264.7 cells, thereby evaluating the cytotoxic effects of the compounds under investigation.^34,35^

### Western Blot Analysis

In brief, RAW264.7 cells were seeded in six-well plates at a density of 5 × 10^5^ cells/well and treated with various concentrations (10, 20, and 40 μM) of compounds **2**–**4**, **15**, and **16** or DEX (5 μM), followed by stimulation with LPS (0.5 μg/mL) for 24 h. Subsequently, cells were collected and lysed by using RIPA buffer. The extracted proteins were then separated by 8% SDS-PAGE and transferred onto PVDF membranes. These membranes were incubated overnight at 4 °C with primary antibodies of iNOS and GAPDH. After washing thrice with TBST, the membranes were exposed to a mouse monoclonal IgG conjugated secondary antibody for 1 h at room temperature. The protein bands were visualized by the LI-COR Odyssey imaging system (Lincoln, NE, USA).

### Statistical Analysis

In the graph, data are presented as mean ± SD. The one-way ANOVA was used to assess the differences among the groups. Analysis of data was derived from the results of GraphPad Prism 8 (GraphPad Software Inc., San Diego, CA, USA). Values of *p* < 0.05 were considered to indicate statistical significance.

## Supporting Information Available

NMR, HRESIMS, IR, and UV spectra of compounds **1**–**11** (PDF)
