# Supplementary material for: NMR-Guided Isolation of Anti-inflammatory Carabranolides from the Fruits of Carpesium abrotanoides L
Source: J Nat Prod. 2024 Jul 10;87(7):1786–97. doi: 10.1021/acs.jnatprod.4c00338 (PMC11287756; doi:10.1021/acs.jnatprod.4c00338)
Supplement: Supplementary file 1 — np4c00338_si_001.pdf [file np4c00338_si_001.pdf]

# Supplementary material

NMR-guided isolation of anti-inflammatory carabranolides from the fruits of *Carpesium abrotanoides* L.

Lu Fu, Can-Can Wang, Wenyue Tian, Zhiyan Liu, Meng-Yu Bao, Jiazheng Liu, Wei Zhang, Li-Ping Bai*, Zhi-Hong Jiang*, Guo-Yuan Zhu*

*State Key Laboratory of Quality Research in Chinese Medicine, Macau Institute for Applied Research in Medicine and Health, Macau University of Science and Technology, Macau SAR 999078, People's Republic of China*

**Corresponding Authors*

E-mail addresses: lpbai@must.edu.mo (L.-P. Bai), zhjiang@must.edu.mo (Z.-H. Jiang), and gyzhu@must.edu.mo (G.-Y. Zhu)

Contents of Supplementary Information


| Position | contents | Page NO. |
|---|---|---|
| Figure S1. | Key NOESY correlations of **1–11** | S5 |
| Figure S2. | $^{1}$H NMR spectrum (600 MHz) of carabrolate A (**1**) in $CDCl_3$ | S6 |
| Figure S3. | $^{13}$C NMR spectrum (150 MHz) of carabrolate A (**1**) in $CDCl_3$ | S6 |
| Figure S4. | DEPT 135 spectrum (150 MHz) of carabrolate A (**1**) in $CDCl_3$ | S7 |
| Figure S5. | HSQC spectrum (600 MHz) of carabrolate A (**1**) in $CDCl_3$ | S7 |
| Figure S6. | $^{1}$H-$^{1}$H COSY spectrum (600 MHz) of carabrolate A (**1**) in $CDCl_3$ | S8 |
| Figure S7. | HMBC spectrum (600 MHz) of carabrolate A (**1**) in $CDCl_3$ | S8 |
| Figure S8. | NOESY spectrum (600 MHz) of carabrolate A (**1**) in $CDCl_3$ | S9 |
| Figure S9. | UV spectrum of carabrolate A (**1**) in MeOH | S9 |
| Figure S10. | IR spectrum (film on KBr plate) of carabrolate A (**1**) | S10 |
| Figure S11. | HRESIMS spectrum of carabrolate A (**1**) | S10 |
| Figure S12. | $^{1}$H NMR spectrum (600 MHz) of carabrolate B (**2**) in $CDCl_3$ | S11 |
| Figure S13. | $^{13}$C NMR spectrum (150 MHz) of carabrolate B (**2**) in $CDCl_3$ | S11 |
| Figure S14. | DEPT 135 spectrum (150 MHz) of carabrolate B (**2**) in $CDCl_3$ | S12 |
| Figure S15. | HSQC spectrum (600 MHz) of carabrolate B (**2**) in $CDCl_3$ | S12 |
| Figure S16. | $^{1}$H-$^{1}$H COSY spectrum (600 MHz) of carabrolate B (**2**) in $CDCl_3$ | S13 |
| Figure S17. | HMBC spectrum (600 MHz) of carabrolate B (**2**) in $CDCl_3$ | S13 |
| Figure S18. | NOESY spectrum (600 MHz) of carabrolate B (**2**) in $CDCl_3$ | S14 |
| Figure S19. | UV spectrum of carabrolate B (**2**) in MeOH | S14 |
| Figure S20. | IR spectrum (film on KBr plate) of carabrolate B (**2**) | S15 |
| Figure S21. | HRESIMS spectrum of carabrolate B (**2**) | S15 |
| Figure S22. | $^{1}$H NMR spectrum (600 MHz) of carabrolate C (**3**) in $CDCl_3$ | S16 |
| Figure S23. | $^{13}$C NMR spectrum (150 MHz) of carabrolateC (**3**) in $CDCl_3$ | S16 |
| Figure S24. | DEPT 135 spectrum (150 MHz) of carabrolate C (**3**) in $CDCl_3$ | S17 |
| Figure S25. | HSQC spectrum (600 MHz) of carabrolate C (**3**) in $CDCl_3$ | S17 |
| Figure S26. | $^{1}$H-$^{1}$H COSY spectrum (600 MHz) of carabrolate C (**3**) in $CDCl_3$ | S18 |
| Figure S27. | HMBC spectrum (600 MHz) of carabrolate C (**3**) in $CDCl_3$ | S18 |
| Figure S28. | NOESY spectrum (600 MHz) of carabrolate C (**3**) in $CDCl_3$ | S19 |
| Figure S29. | UV spectrum of carabrolate C (**3**) in MeOH | S19 |
| Figure S30. | IR spectrum (film on KBr plate) of carabrolate C (**3**) | S20 |
| Figure S31. | HRESIMS spectrum of carabrolate C (**3**) | S20 |
| Figure S32. | $^{1}$H NMR spectrum (600 MHz) of carabrolate D (**4**) in $CDCl_3$ | S21 |
| Figure S33. | $^{13}$C NMR spectrum (150 MHz) of carabrolate D (**4**) in $CDCl_3$ | S21 |
| Figure S34. | HSQC spectrum (600 MHz) of carabrolate D (**4**) in $CDCl_3$ | S22 |
| Figure S35. | DEPT 135 spectrum (150 MHz) of carabrolate D (**4**) in $CDCl_3$ | S22 |
| Figure S36. | $^{1}$H-$^{1}$H COSY spectrum (600 MHz) of carabrolate D (**4**) in $CDCl_3$ | S23 |
| Figure S37. | HMBC spectrum (600 MHz) of carabrolate D (**4**) in $CDCl_3$ | S23 |
| Figure S38. | NOESY spectrum (600 MHz) of carabrolate D (**4**) in $CDCl_3$ | S24 |
| Figure S39. | UV spectrum of carabrolate D (**4**) in MeOH | S24 |
| Figure S40. | IR spectrum (film on KBr plate) of carabrolate D (**4**) | S25 |
| Figure S41. | HRESIMS spectrum of carabrolate D (**4**) | S25 |
| Figure S42. | $^{1}$H NMR spectrum (600 MHz) of carabrolate E (**5**) in $CDCl_3$ | S26 |
| Figure S43. | $^{13}$C NMR spectrum (150 MHz) of carabrolate E (**5**) in $CDCl_3$ | S26 |
| Figure S44. | DEPT 135 spectrum (150 MHz) of carabrolate E (**5**) in $CDCl_3$ | S27 |
| Figure S45. | HSQC spectrum (600 MHz) of carabrolate E (**5**) in $CDCl_3$ | S27 |

| | | |
|---|---|---|
| Figure S46. | $^1$H-$^1$H COSY spectrum (600 MHz) of carabrolate E (**5**) in $CDCl_3$ | S28 |
| Figure S47. | HMBC spectrum (600 MHz) of carabrolate E (**5**) in $CDCl_3$ | S28 |
| Figure S48. | NOESY spectrum (600 MHz) of carabrolate E (**5**) in $CDCl_3$ | S29 |
| Figure S49. | UV spectrum of carabrolate E (**5**) in MeOH | S29 |
| Figure S50. | IR spectrum (film on KBr plate) of carabrolate E (**5**) | S30 |
| Figure S51. | HRESIMS spectrum of carabrolate E (**5**) $CDCl_3$ | S30 |
| Figure S52. | $^1$H NMR spectrum (600 MHz) of carabrolate F (**6**) in $CDCl_3$ | S31 |
| Figure S53. | $^{13}$C NMR spectrum (150 MHz) of carabrolate G (**6**) in $CDCl_3$ | S31 |
| Figure S54. | DEPT 135 spectrum (150 MHz) of carabrolate F (**6**) in $CDCl_3$ | S32 |
| Figure S55. | HSQC spectrum (600 MHz) of carabrolate F (**6**) in $CDCl_3$ | S32 |
| Figure S56. | $^1$H-$^1$H COSY spectrum (600 MHz) of carabrolate F (**6**) in $CDCl_3$ | S33 |
| Figure S57. | HMBC spectrum (600 MHz) of carabrolate F (**6**) in $CDCl_3$ | S33 |
| Figure S58. | NOESY spectrum (600 MHz) of carabrolate F (**6**) in $CDCl_3$ | S34 |
| Figure S59. | UV spectrum of carabrolate F (**6**) in MeOH | S34 |
| Figure S60. | IR spectrum (film on KBr plate) of carabrolate F (**6**) | S35 |
| Figure S61. | HRESIMS spectrum of carabrolate F (**6**) | S35 |
| Figure S62. | $^1$H NMR spectrum (600 MHz) of carabrolate G (**7**) in $CDCl_3$ | S36 |
| Figure S63. | $^{13}$C NMR spectrum (150 MHz) of carabrolate G (**7**) in $CDCl_3$ | S36 |
| Figure S64. | DEPT 135 spectrum (150 MHz) of carabrolate G (**7**) in $CDCl_3$ | S37 |
| Figure S65. | HSQC spectrum (600 MHz) of carabrolate G (**7**) in $CDCl_3$ | S37 |
| Figure S66. | $^1$H-$^1$H COSY spectrum (600 MHz) of carabrolate G (**7**) in $CDCl_3$ | S38 |
| Figure S67. | HMBC spectrum (600 MHz) of carabrolate G (**7**) in $CDCl_3$ | S38 |
| Figure S68. | NOESY spectrum (600 MHz) of carabrolate G (**7**) in $CDCl_3$ | S39 |
| Figure S69. | UV spectrum of carabrolate G (**7**) in MeOH | S39 |
| Figure S70. | IR spectrum (film on KBr plate) of carabrolate G (**7**) | S40 |
| Figure S71. | HRESIMS spectrum of carabrolate G (**7**) | S40 |
| Figure S72. | $^1$H NMR spectrum (600 MHz) of carabrolate H (**8**) in $CDCl_3$ | S41 |
| Figure S73. | $^{13}$C NMR spectrum (150 MHz) of carabrolate H (**8**) in $CDCl_3$ | S41 |
| Figure S74. | DEPT 135 spectrum (150 MHz) of carabrolate H (**8**) in $CDCl_3$ | S42 |
| Figure S75. | HSQC spectrum (600 MHz) of carabrolate H (**8**) in $CDCl_3$ | S42 |
| Figure S76. | $^1$H-$^1$H COSY spectrum (600 MHz) of carabrolate H (**8**) in $CDCl_3$ | S43 |
| Figure S77. | HMBC spectrum (600 MHz) of carabrolate H (**8**) in $CDCl_3$ | S43 |
| Figure S78. | NOESY spectrum (600 MHz) of carabrolate H (**8**) in $CDCl_3$ | S44 |
| Figure S79. | UV spectrum of carabrolate H (**8**) in MeOH | S44 |
| Figure S80. | IR spectrum (film on KBr plate) of carabrolate H (**8**) | S45 |
| Figure S81. | HRESIMS spectrum of ccarabrolate H (**8**) | S45 |
| Figure S82. | $^1$H NMR spectrum (600 MHz) of carabrolate I (**9**) in $CDCl_3$ | S46 |
| Figure S83. | $^{13}$C NMR spectrum (150 MHz) of carabrolate I (**9**) in $CDCl_3$ | S46 |
| Figure S84. | HSQC spectrum (600 MHz) of carabrolate I (**9**) in $CDCl_3$ | S47 |
| Figure S85. | DEPT 135 spectrum (150 MHz) of carabrolate I (**9**) in $CDCl_3$ | S47 |
| Figure S86. | $^1$H-$^1$H COSY spectrum (600 MHz) of carabrolate I (**9**) in $CDCl_3$ | S48 |
| Figure S87. | HMBC spectrum (600 MHz) of carabrolate I (**9**) in $CDCl_3$ | S48 |
| Figure S88. | NOESY spectrum (600 MHz) of carabrolate I (**9**) in $CDCl_3$ | S49 |
| Figure S89. | UV spectrum of carabrolate I (**9**) in MeOH | S49 |
| Figure S90. | IR spectrum (film on KBr plate) of carabrolate I (**9**) | S50 |
| Figure S91. | HRESIMS spectrum of carabrolate I (**9**) | S50 |
| Figure S92. | $^1$H NMR spectrum (600 MHz) of carabrolate J (**10**) in $CDCl_3$ | S51 |

| | | |
|---|---|---|
| Figure S93. | $^{13}C$ NMR spectrum (150 MHz) of carabrolate J (**10**) in $CDCl_3$ | S51 |
| Figure S94. | DEPT 135 spectrum (150 MHz) of carabrolate J (**10**) in $CDCl_3$ | S52 |
| Figure S95. | HSQC spectrum (600 MHz) of carabrolate J (**10**) in $CDCl_3$ | S52 |
| Figure S96. | $^1H$-$^1H$ COSY spectrum (600 MHz) of carabrolate J (**10**) in $CDCl_3$ | S53 |
| Figure S97. | HMBC spectrum (600 MHz) of carabrolate J (**10**) in $CDCl_3$ | S53 |
| Figure S98. | NOESY spectrum (600 MHz) of carabrolate J (**10**) in $CDCl_3$ | S54 |
| Figure S99. | UV spectrum of carabrolate J (**10**) in MeOH | S54 |
| Figure S100. | IR spectrum (film on KBr plate) of carabrolate J (**10**) | S55 |
| Figure S101. | HRESIMS spectrum of carabrolate J (**10**) $CDCl_3$ | S55 |
| Figure S102. | $^1H$ NMR spectrum (600 MHz) of carabrolate K (**11**) in $CDCl_3$ | S56 |
| Figure S103. | $^{13}C$ NMR spectrum (150 MHz) of carabrolate K (**11**) in $CDCl_3$ | S56 |
| Figure S104. | DEPT 135 spectrum (150 MHz) of carabrolate K (**11**) in $CDCl_3$ | S57 |
| Figure S105. | HSQC spectrum (600 MHz) of carabrolate K (**11**) in $CDCl_3$ | S57 |
| Figure S106. | $^1H$-$^1H$ COSY spectrum (600 MHz) of carabrolate K (**11**) in $CDCl_3$ | S58 |
| Figure S107. | HMBC spectrum (600 MHz) of carabrolate K (**11**) in $CDCl_3$ | S58 |
| Figure S108. | NOESY spectrum (600 MHz) of carabrolate K (**11**) in $CDCl_3$ | S59 |
| Figure S109. | UV spectrum of carabrolate K (**11**) in MeOH | S59 |
| Figure S110. | IR spectrum (film on KBr plate) of carabrolate K (**11**) | S60 |
| Figure S111. | HRESIMS spectrum of carabrolate K (**11**) $CDCl_3$ | S60 |
| Figure S112. | HRESIMS spectrum of the ketal of carabrolate E (**5**) | S61 |
| Figure S113. | HRESIMS spectrum of the ketal of carabrolate F (**6**) | S61 |
| Figure S114. | MS/MS spectrum (positive mode) of carabrolate G (**7**). | S62 |
| Figure S115. | MS/MS spectrum (positive mode) of carabrolate K (**11**). | S62 |
| Figure S116. | $^1H$ NMR spectra (600 MHz) of **5**, **5a**, **5b**, and **5c** in $C_5D_5N$. | S63 |
| Figure S117. | $^1H$-$^1H$ COSY spectra (600 MHz) of **5b** and **5c** in $C_5D_5N$. | S63 |
| Figure S118. | Enlarged $^1H$-$^1H$ COSY spectra (600 MHz) of **5b** and **5c** in $C_5D_5N$. | S64 |
| Figure S119. | $^1H$ NMR spectra (600 MHz) of **6**, **6a**, **6b**, and **6c** in $C_5D_5N$. | S64 |
| Figure S120. | $^1H$-$^1H$ COSY spectra (600 MHz) of **6b** and **6c** in $C_5D_5N$. | S65 |
| Figure S121. | Enlarged $^1H$-$^1H$ COSY spectra (600 MHz) of **6b** and **6c** in $C_5D_5N$. | S65 |
| Figure S122. | $^1H$ NMR spectra (600 MHz) of **9**, **9a**, and **9b** in $C_5D_5N$. | S66 |
| Figure S123. | $^1H$-$^1H$ COSY spectra (600 MHz) of **9a** and **9b** in $C_5D_5N$. | S66 |
| Figure S124. | Enlarged $^1H$-$^1H$ COSY spectra (600 MHz) of **9a** and **9b** in $C_5D_5N$. | S67 |

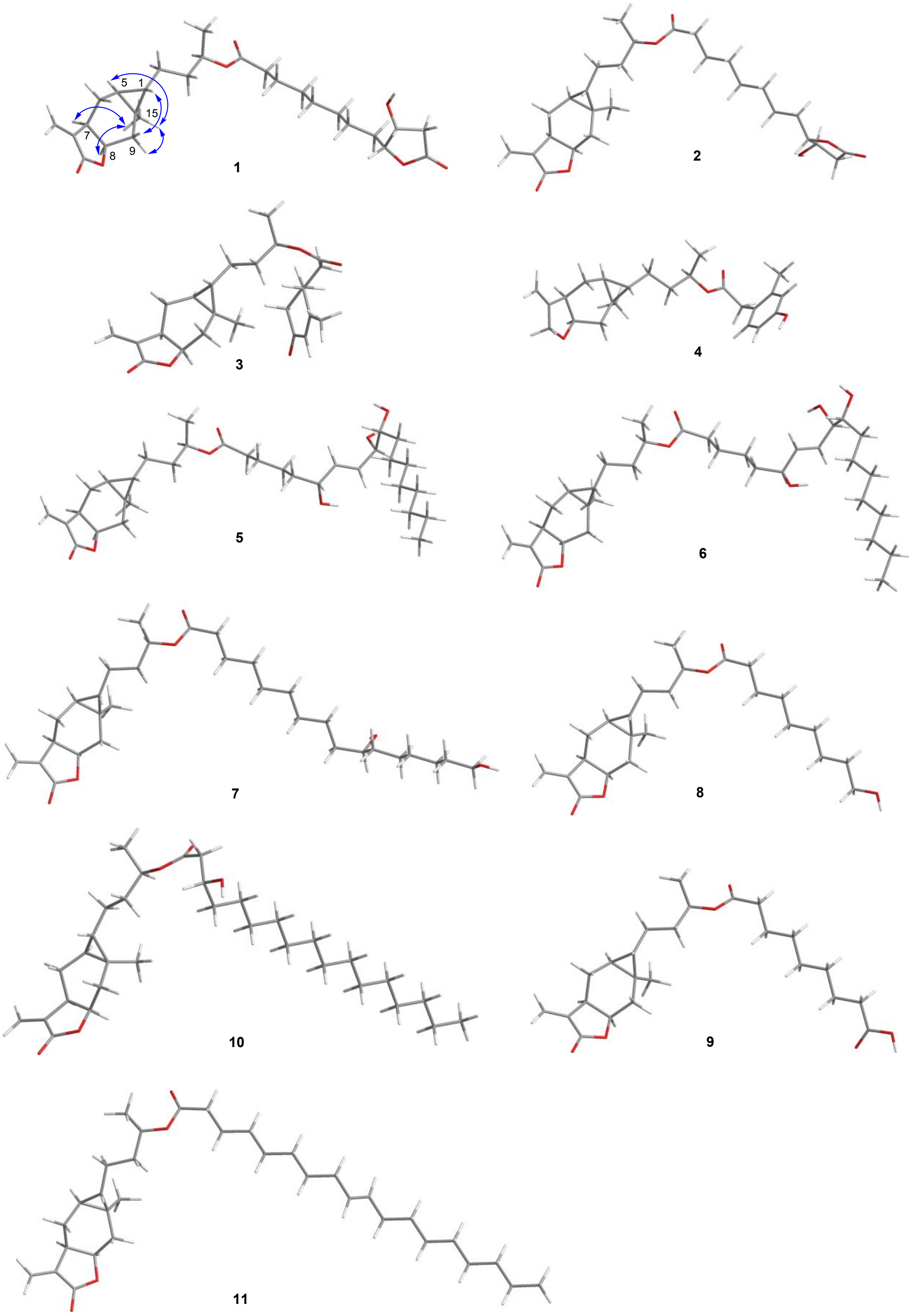


Figure S1.Key NOESY correlations of **1–11**.

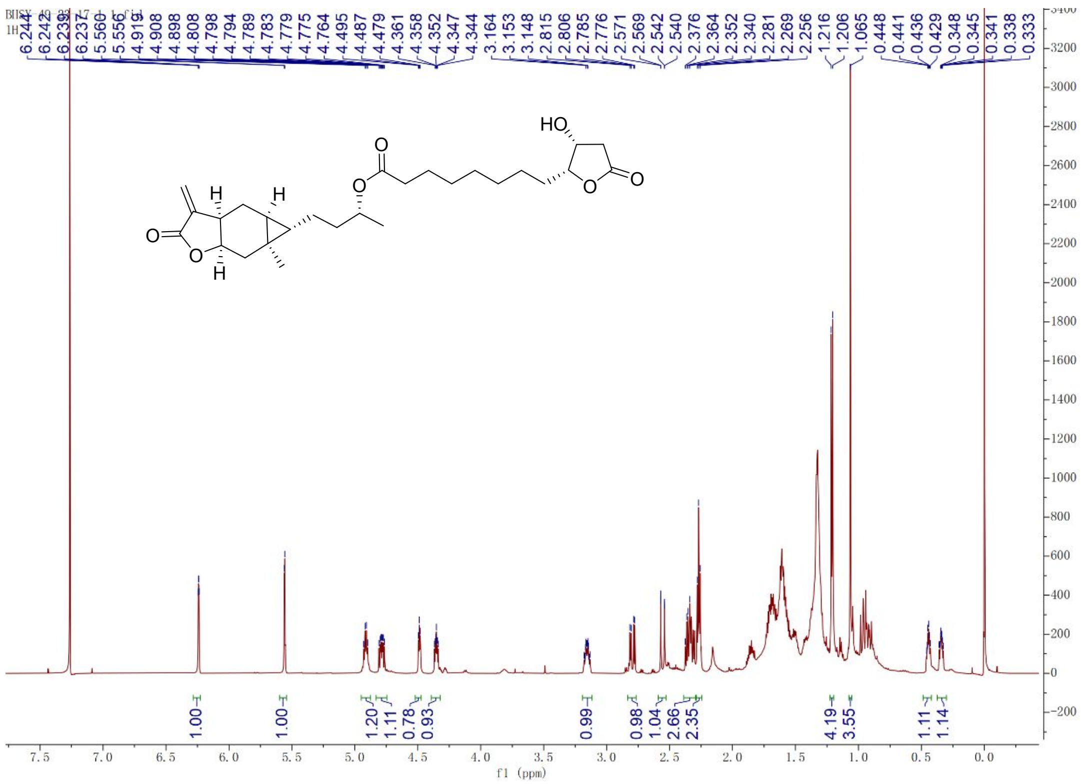


Figure S2. $^{1}H$ NMR spectrum (600 MHz) of carabrolate A (**1**) in $CDCl_3$

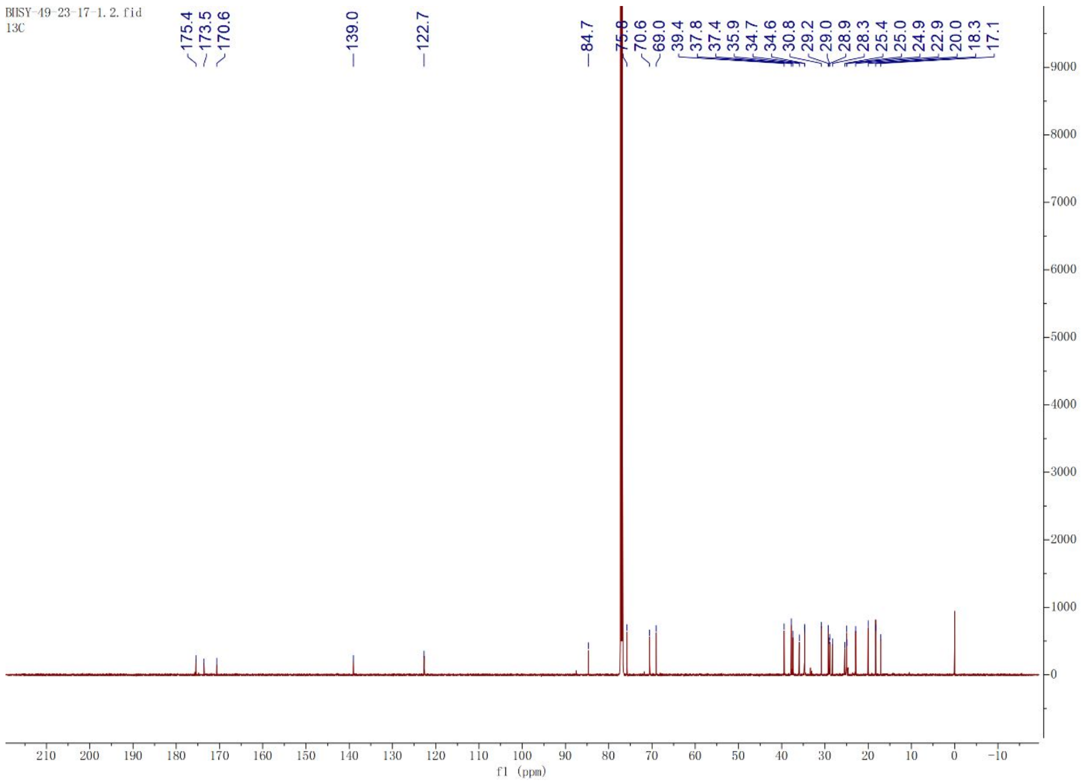


Figure S3. $^{13}C$ NMR spectrum (150 MHz) of carabrolate (**1**) in $CDCl_3$

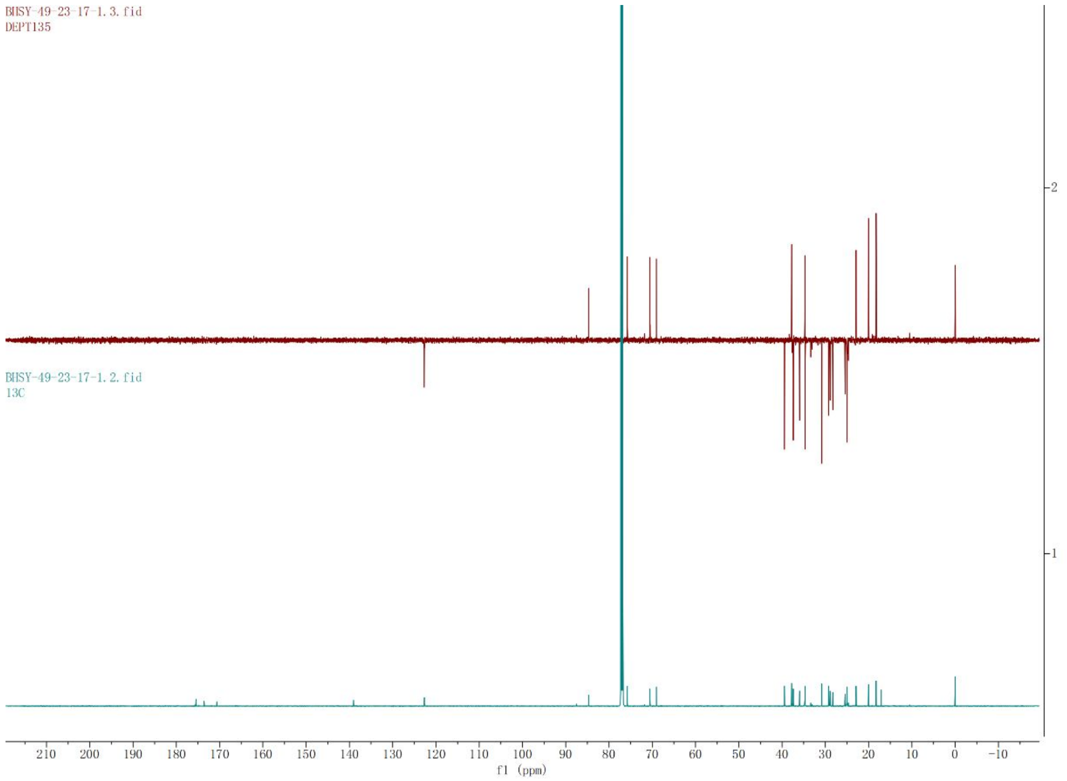


Figure S4. DEPT 135 spectrum (150 MHz) of carabrolate A (**1**) in $CDCl_3$

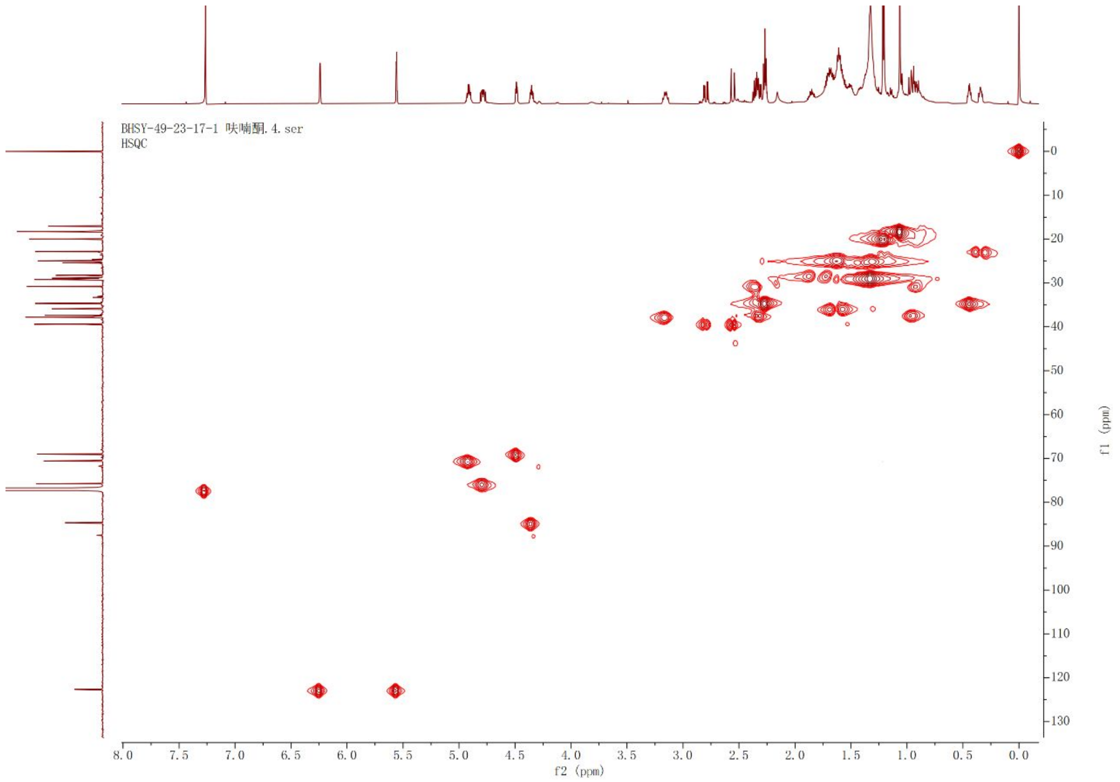


Figure S5. HSQC spectrum (600 MHz) of carabrolate A (**1**) in $CDCl_3$

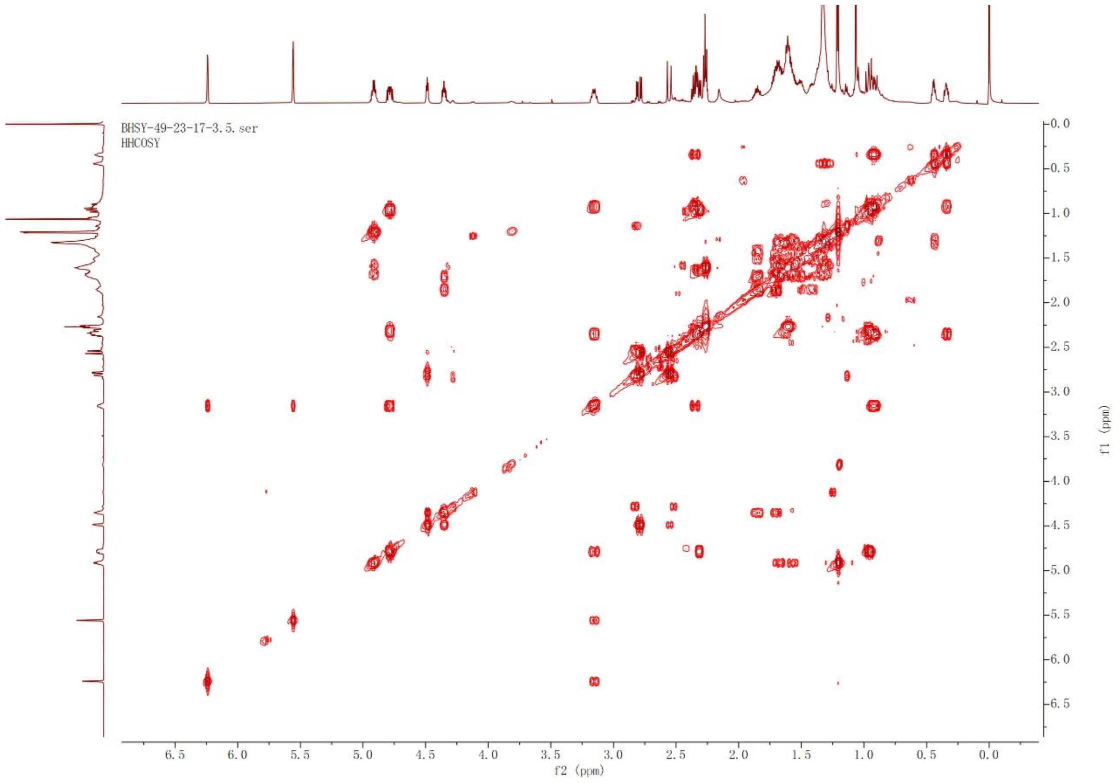


Figure S6. $^1H$-$^1H$ COSY spectrum (600 MHz) of carabrolate A (**1**) in $CDCl_3$

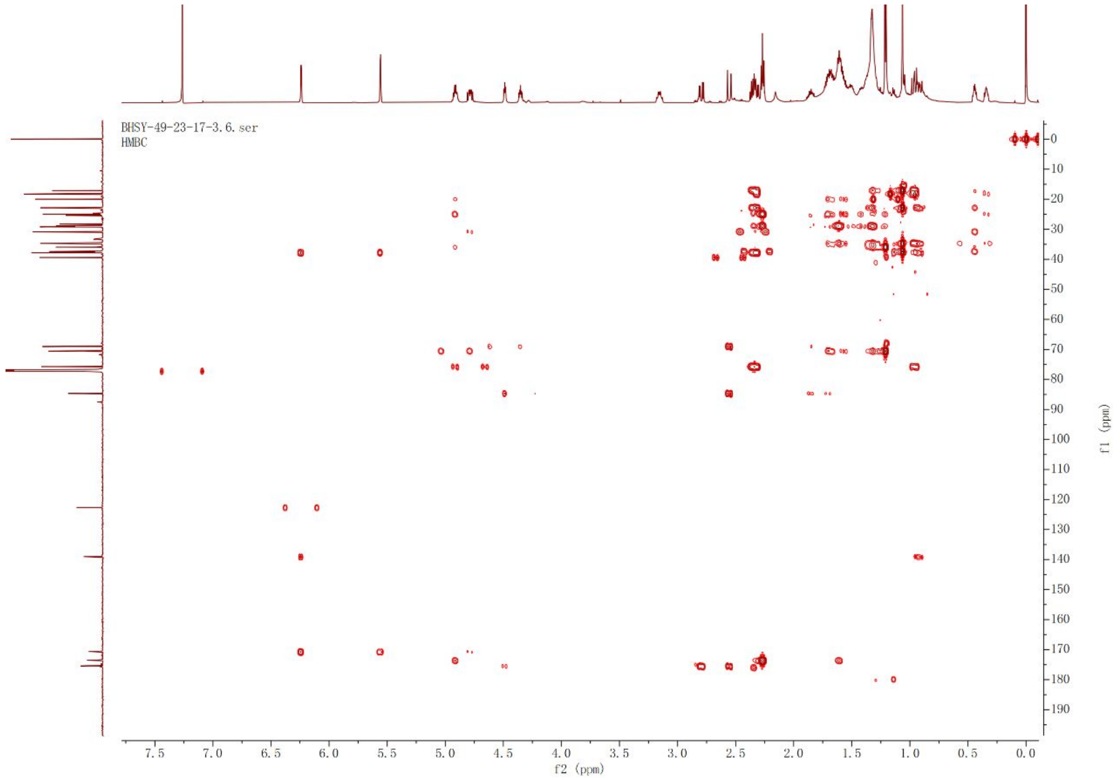


Figure S7. HMBC spectrum (600 MHz) of carabrolate A (**1**) in $CDCl_3$

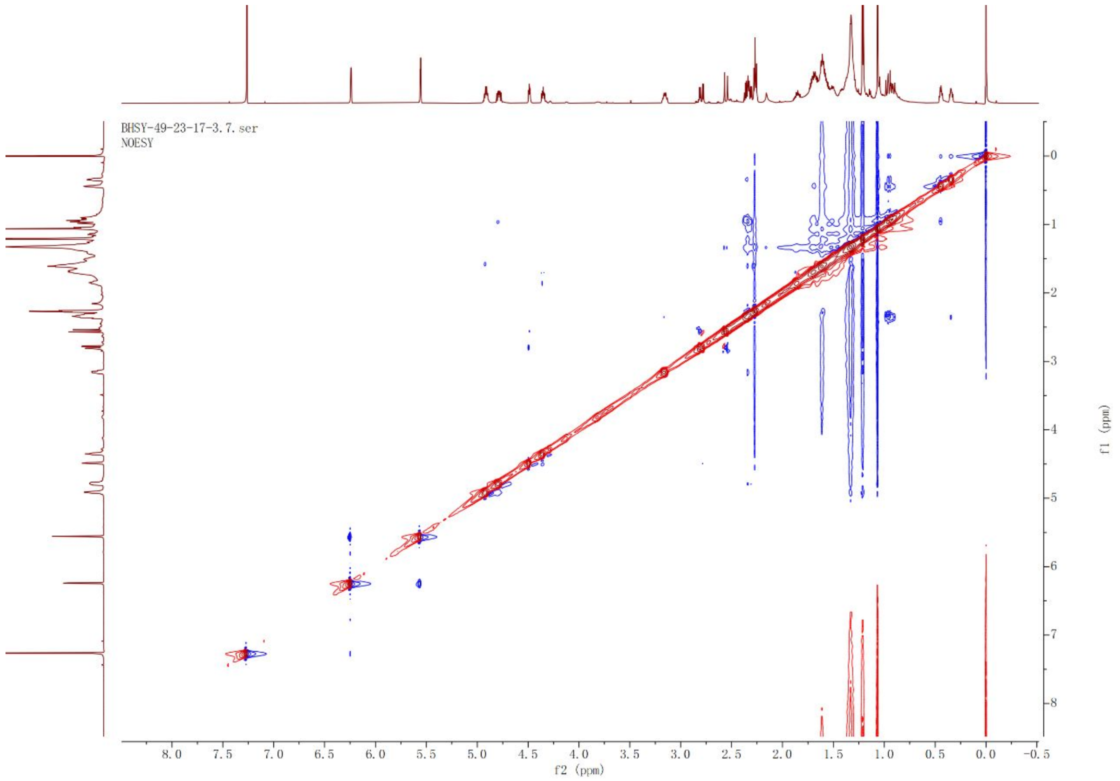


Figure S8. NOESY spectrum (600 MHz) of carabrolate A (**1**) in $CDCl_3$

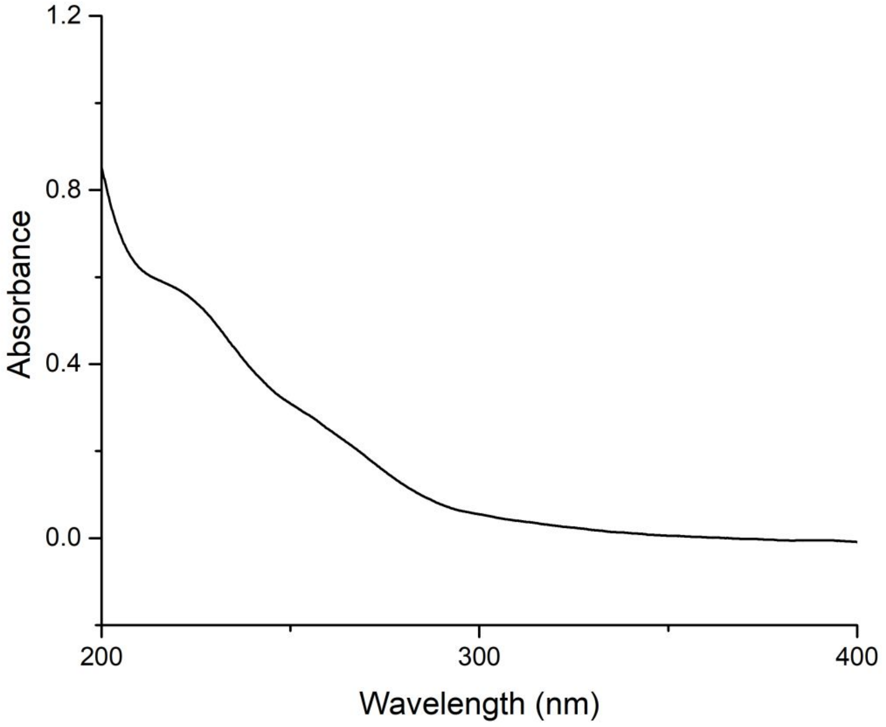


Figure S9. UV spectrum of carabrolate A (**1**) in MeOH

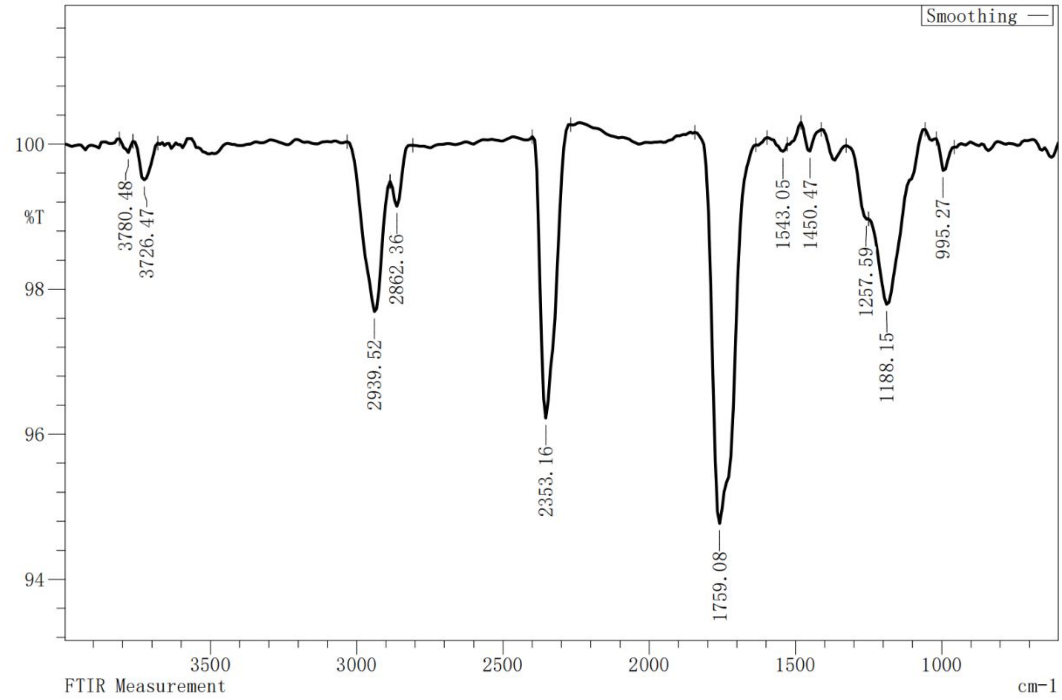


Figure S10. IR spectrum (film on KBr plate) of carabrolate A (**1**)

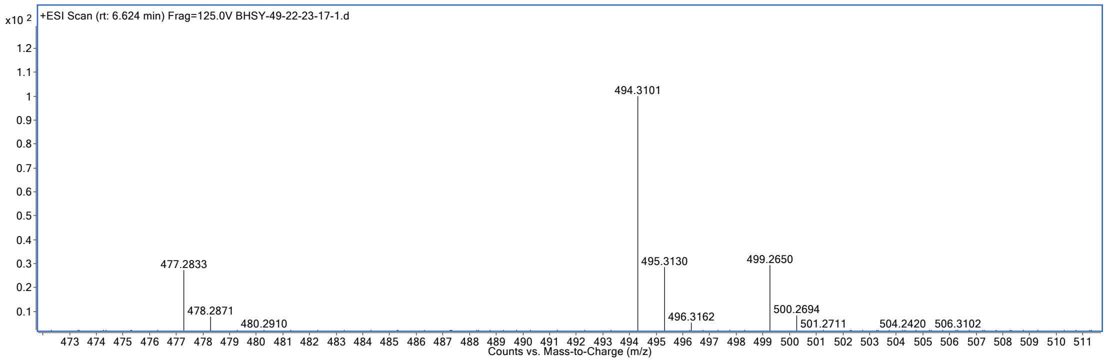


| Formula (M) | Score (MFG) | Mass | Mass (MFG) | m/z (Calc) | Diff (ppm) | Ion Formula | m/z |
|---|---|---|---|---|---|---|---|
| $C_{27}H_{40}O_7$ | 96.5 | 476.276 | 476.2774 | 477.2847 | 2.9 | $C_{27}H_{41}O_7$ | 477.2833 |
| $C_{27}H_{40}O_7$ | 97.62 | 476.2763 | 476.2774 | 494.3112 | 2.37 | $C_{27}H_{44}NO_7$ | 494.3101 |

Figure S11. HRESIMS spectrum of carabrolate A (**1**)

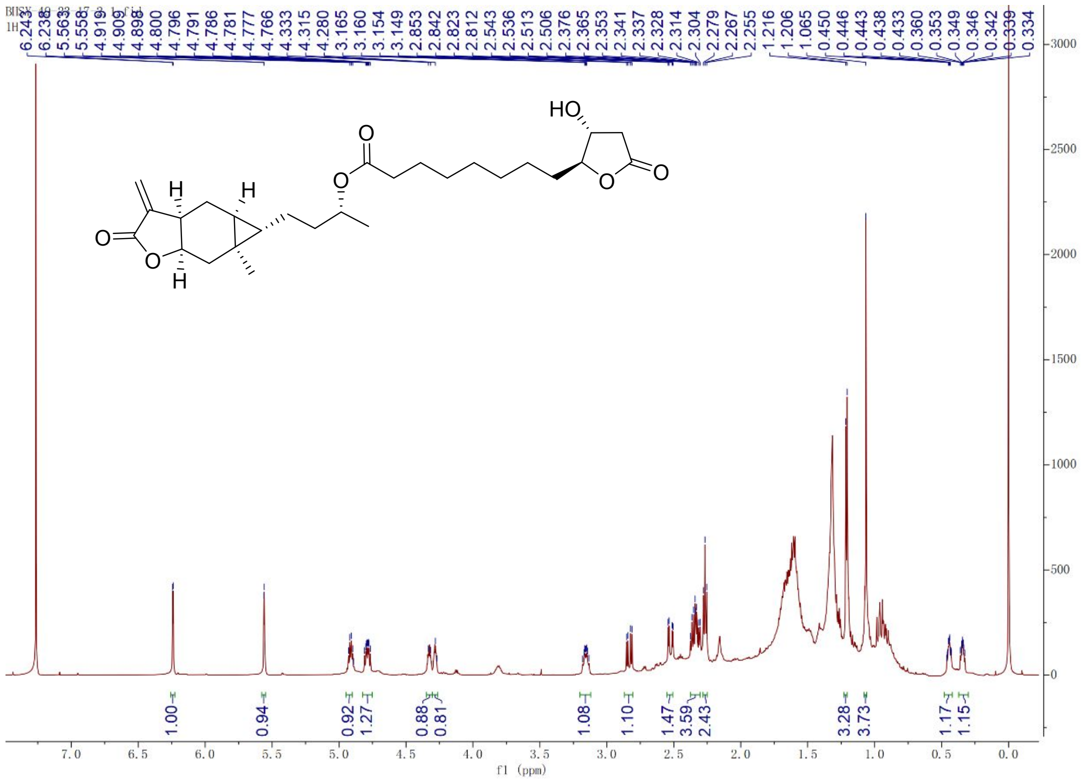

Figure S12. $^{1}$H NMR spectrum (600 MHz) of carabrolate B (**2**) in $CDCl_3$

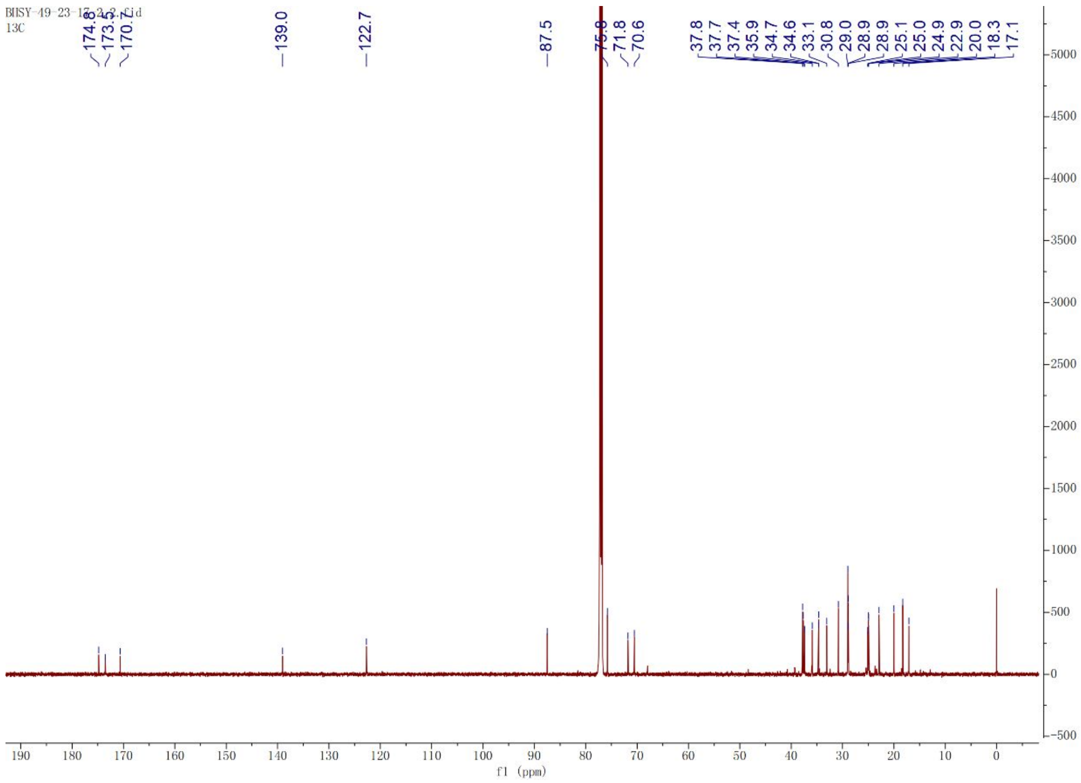

Figure S13. $^{13}$C NMR spectrum (150 MHz) of carabrolate B (**2**) in $CDCl_3$

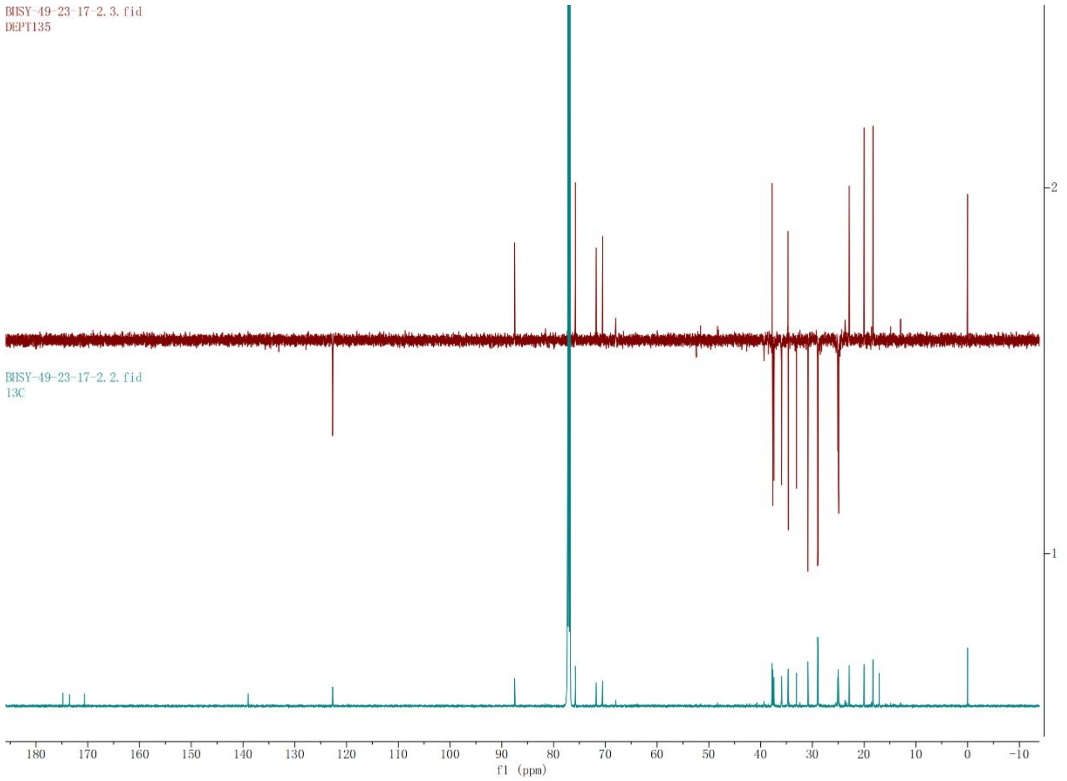


Figure S14. DEPT 135 spectrum (150 MHz) of carabrolate B (**2**) in $CDCl_3$

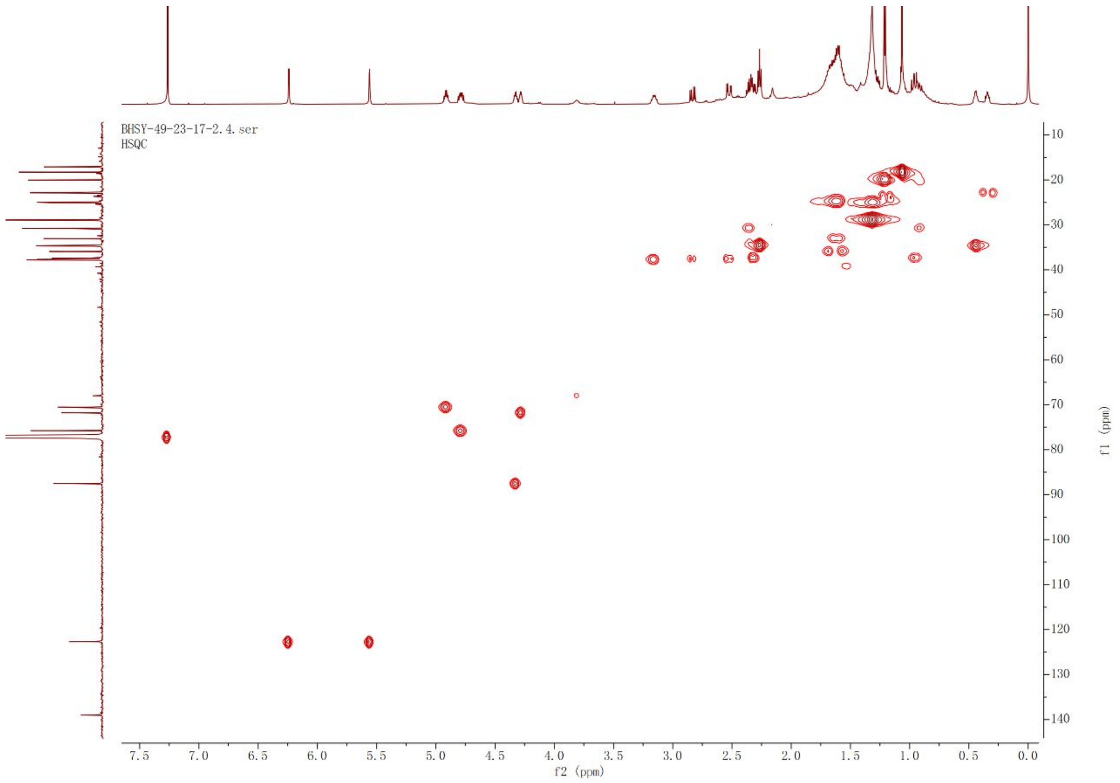


Figure S15. HSQC spectrum (600 MHz) of carabrolate B (**2**) in $CDCl_3$

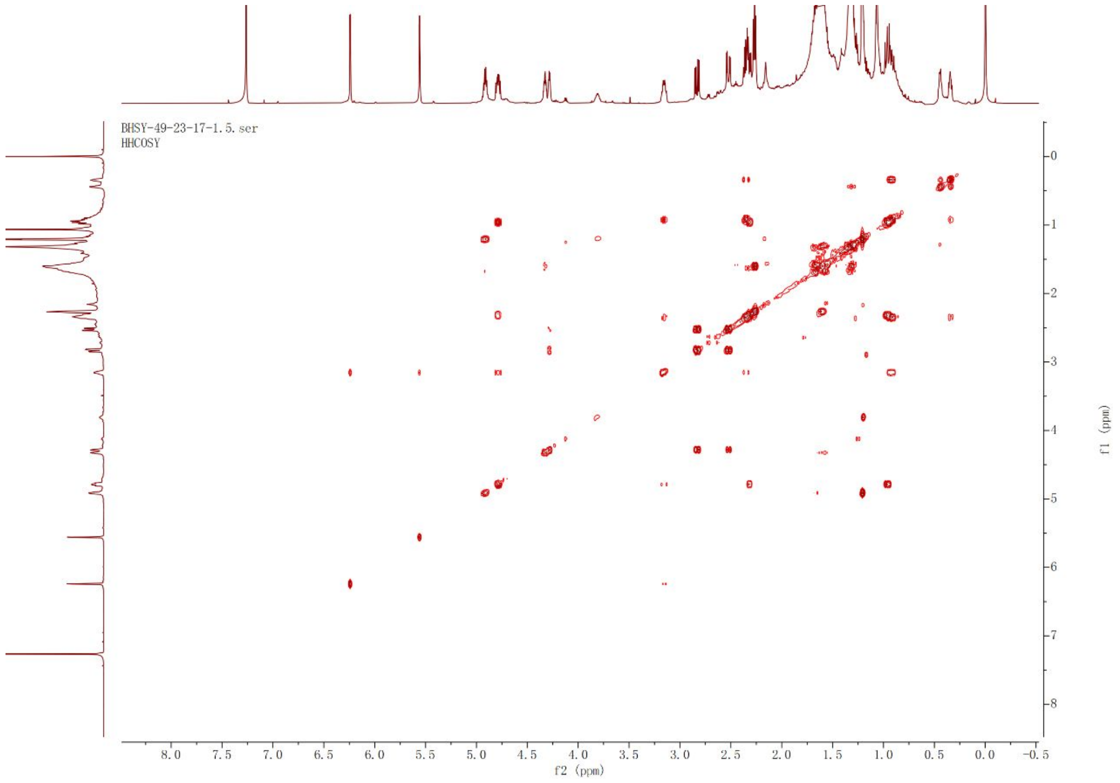


Figure S16. $^1$H-$^1$H COSY spectrum (600 MHz) of carabrolate B (**2**) in $CDCl_3$

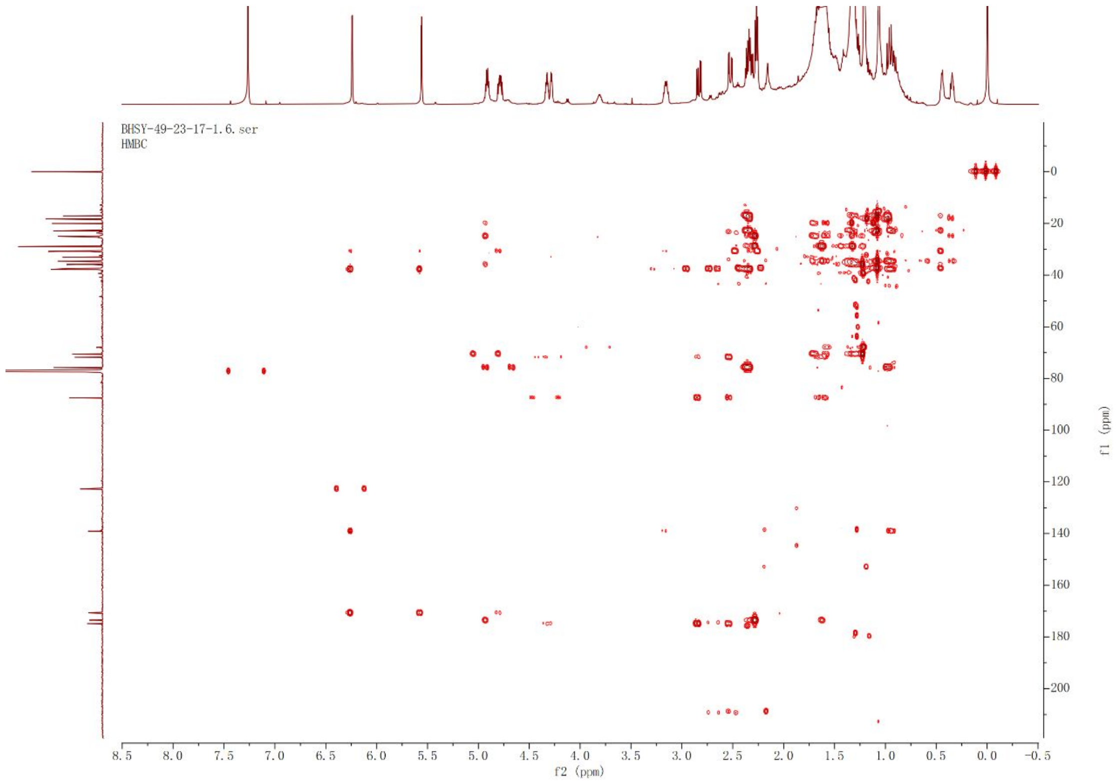


Figure S17. HMBC spectrum (600 MHz) of carabrolate B (**2**) in $CDCl_3$

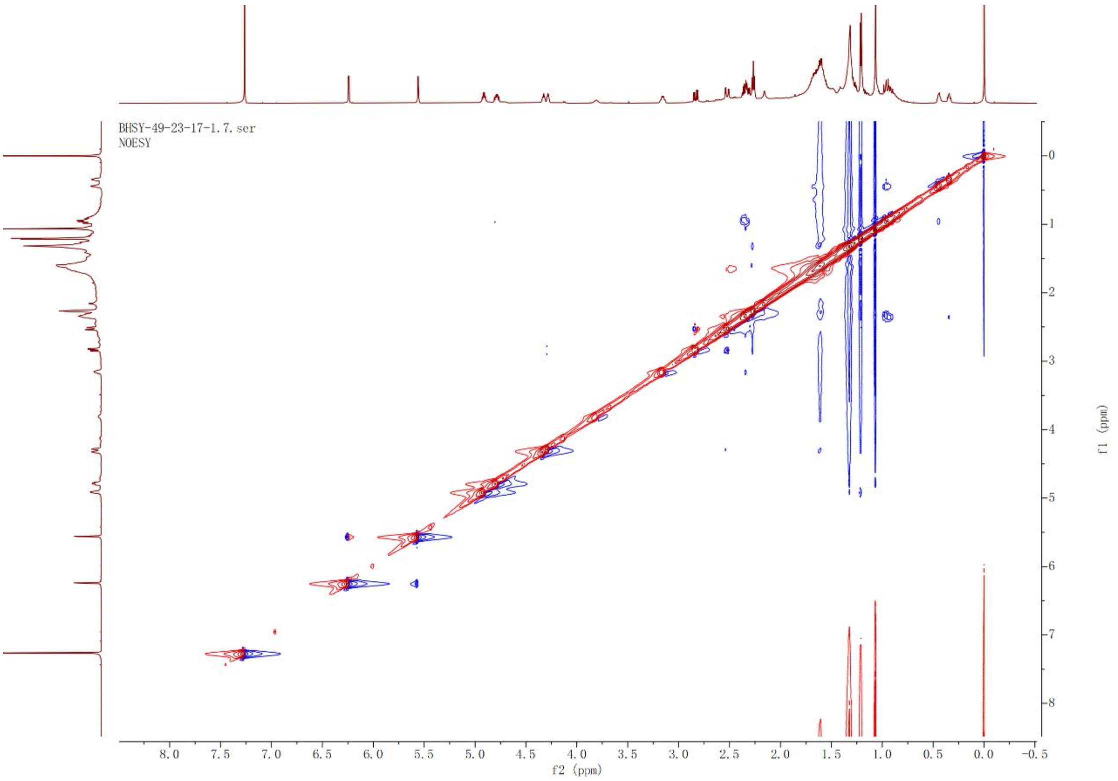


Figure S18. NOESY spectrum (600 MHz) of carabrolate B (**2**) in $CDCl_3$

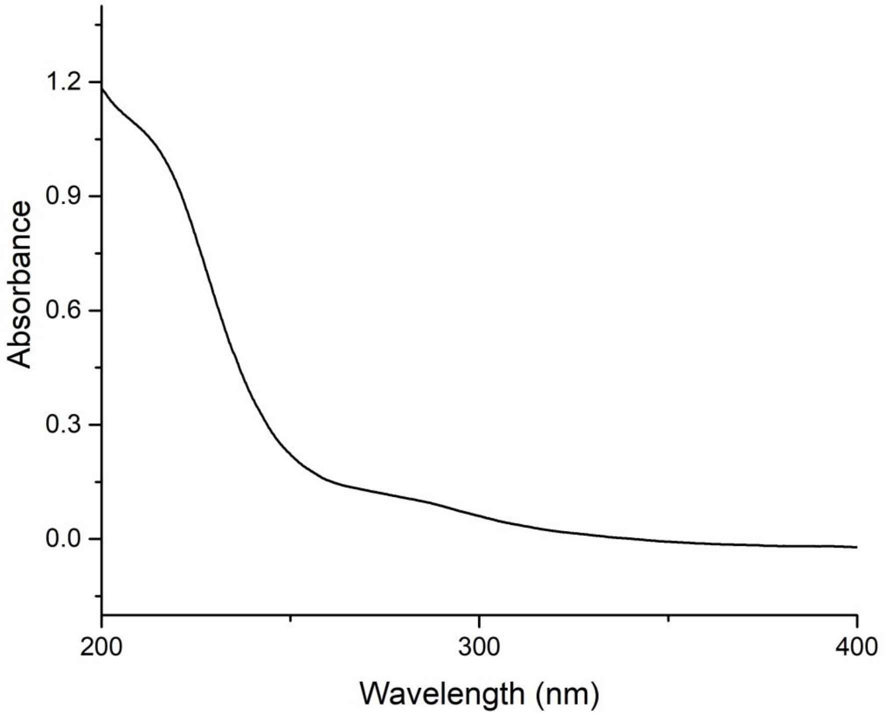


Figure S19. UV spectrum of carabrolate B (**2**) in MeOH

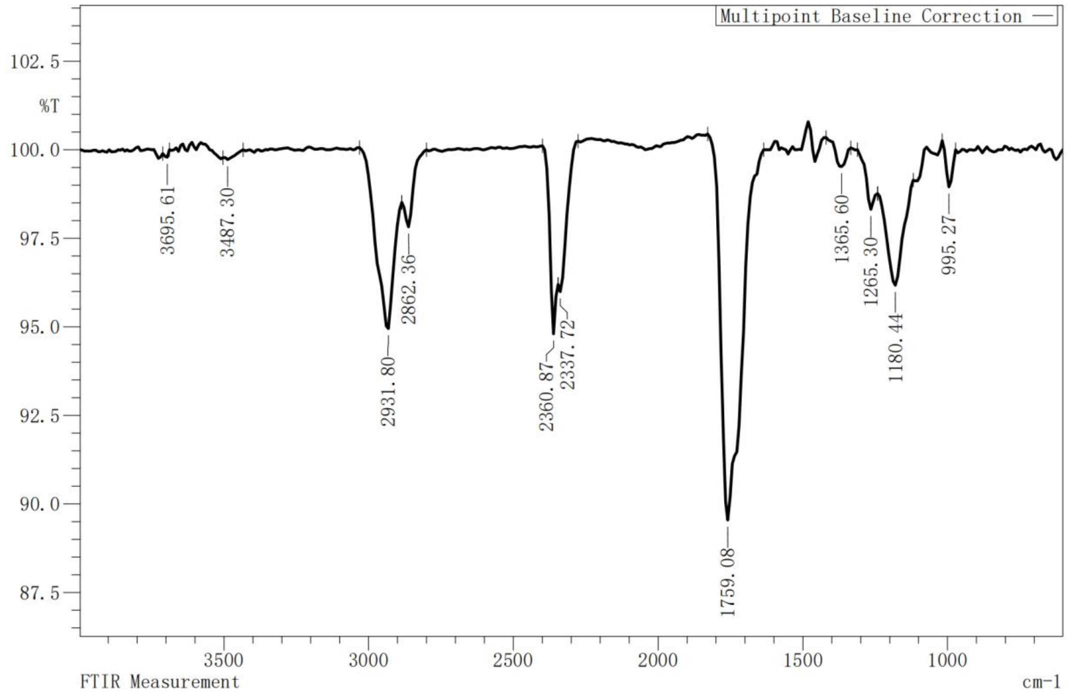


Figure S20. IR spectrum (film on KBr plate) of carabrolate B (**2**)

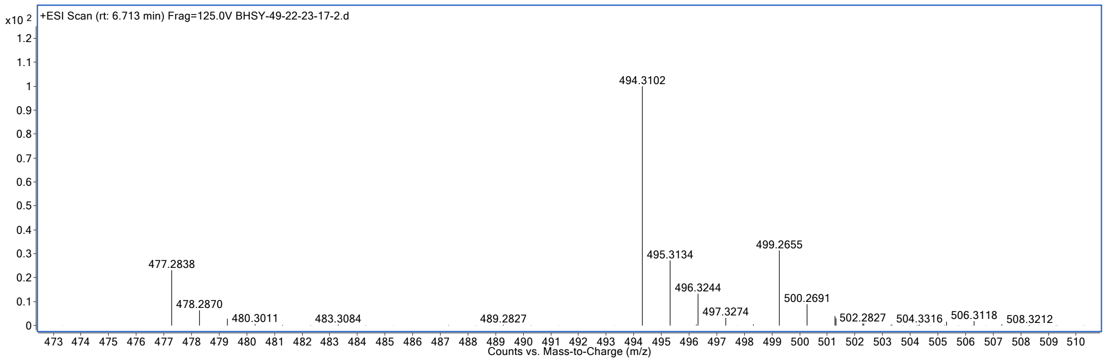


| Formula (M) | Score (MFG) | Mass | Mass (MFG) | m/z (Calc) | Diff (ppm) | Ion Formula | m/z |
|---|---|---|---|---|---|---|---|
| $C_{27}H_{40}O_7$ | 98.54 | 476.2765 | 476.2774 | 477.2847 | 1.85 | $C_{27}H_{41}O_7$ | 477.2838 |
| $C_{27}H_{40}O_7$ | 98.01 | 476.2764 | 476.2774 | 494.3112 | 2.16 | $C_{27}H_{44}NO_7$ | 494.3102 |

Figure S21. HRESIMS spectrum of carabrolate B (**2**).

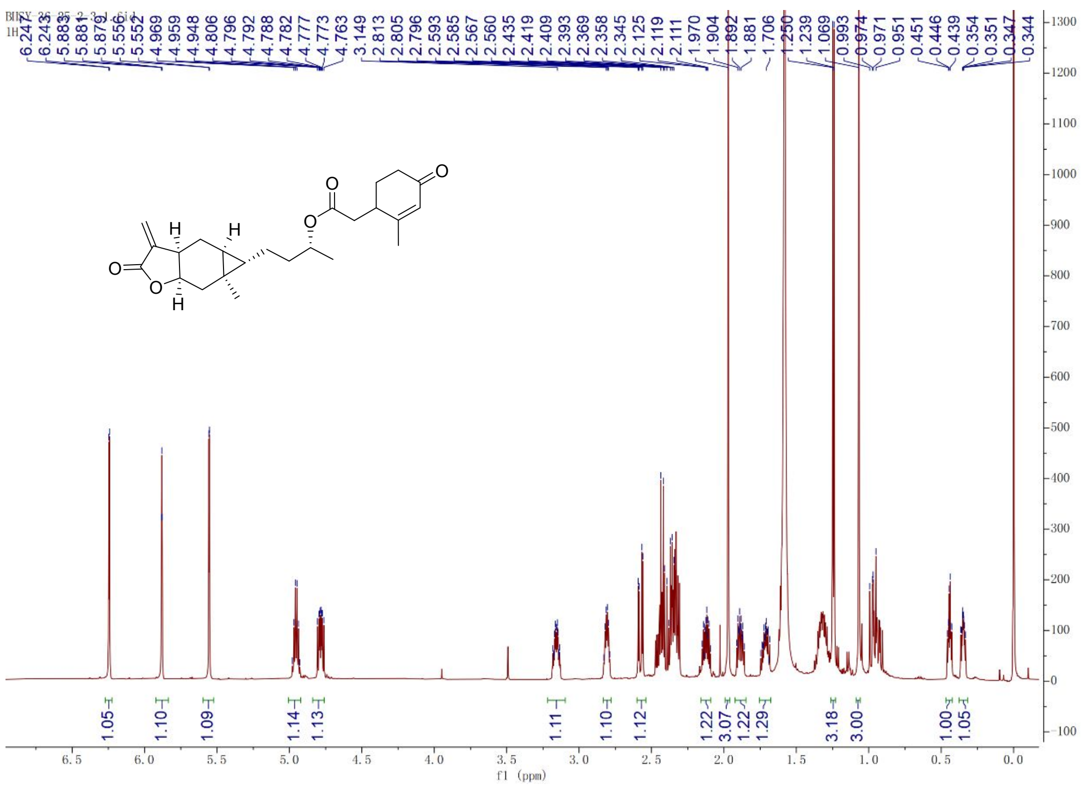

Figure S22. $^{1}H$ NMR spectrum (600 MHz) of carabrolate C (**3**) in $CDCl_3$

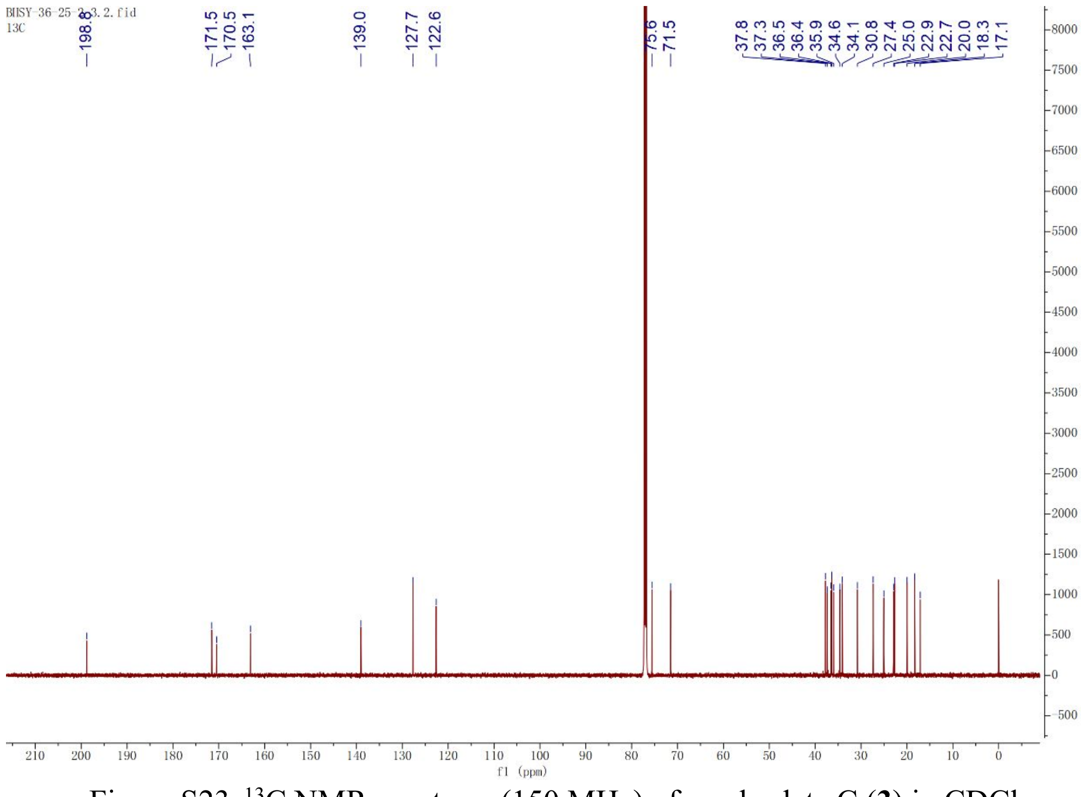

Figure S23. $^{13}C$ NMR spectrum (150 MHz) of carabrolate C (**3**) in $CDCl_3$

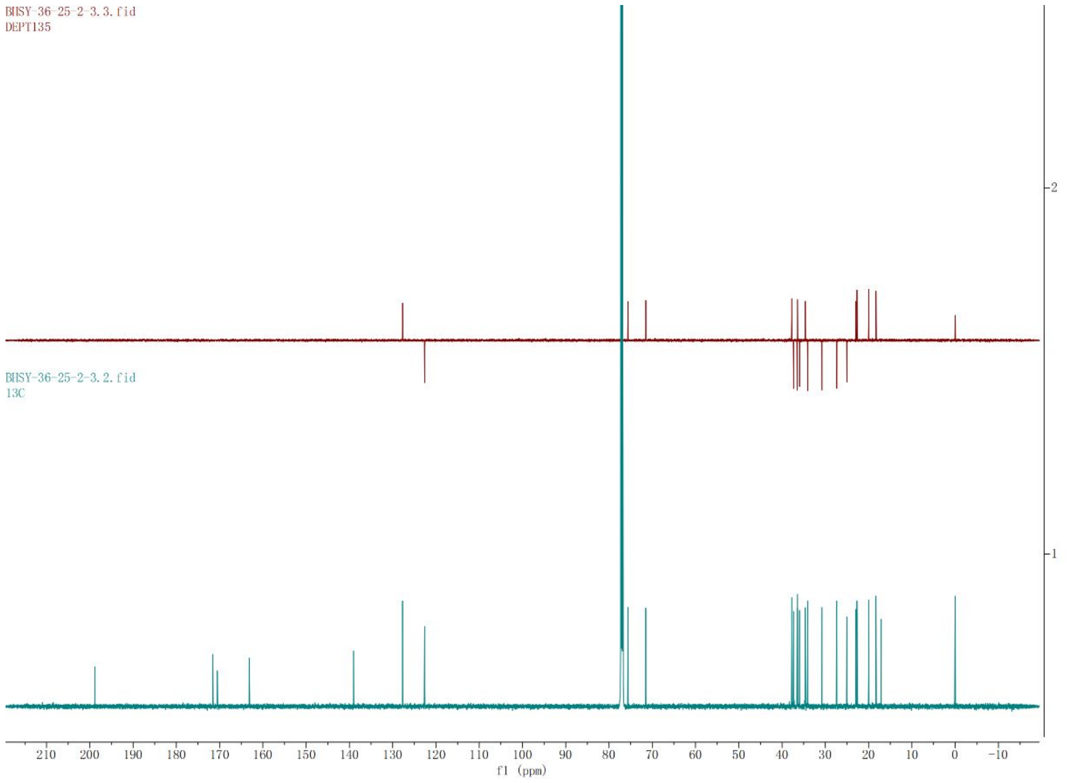


Figure S24. DEPT 135 spectrum (150 MHz) of carabrolate C (**3**) in $CDCl_3$

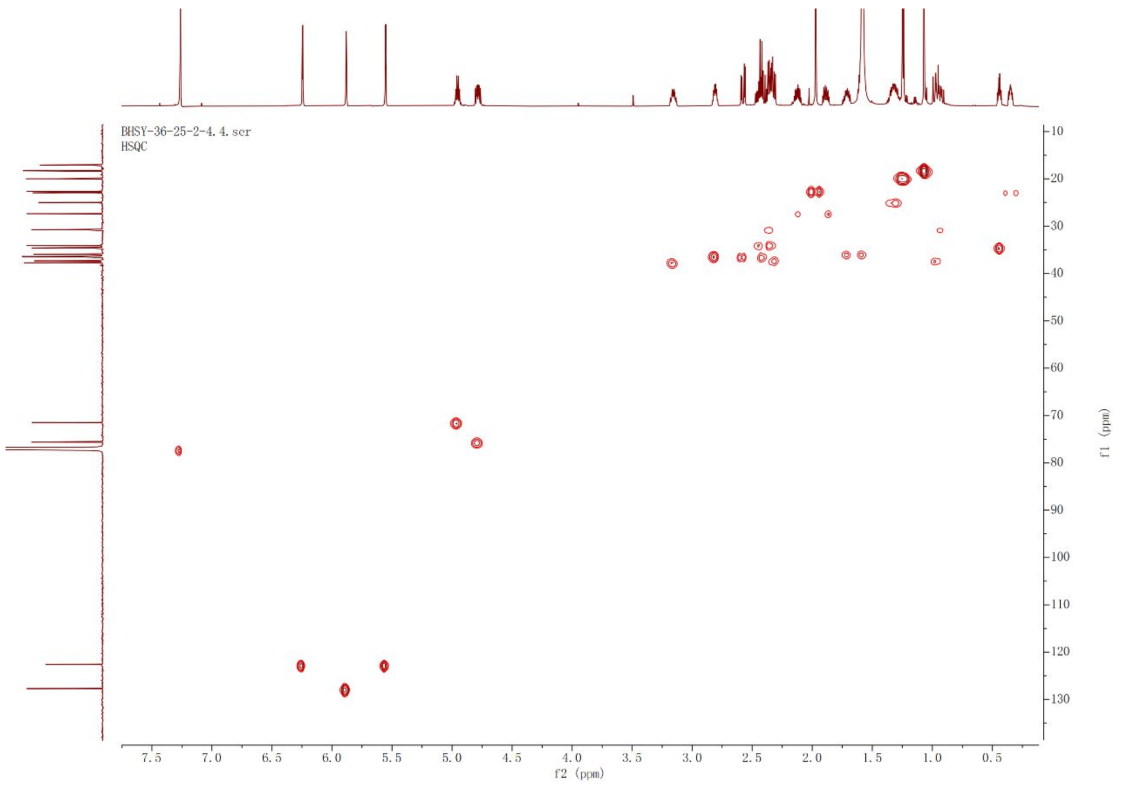


Figure S25. HSQC spectrum (600 MHz) of carabrolate C (**3**) in $CDCl_3$

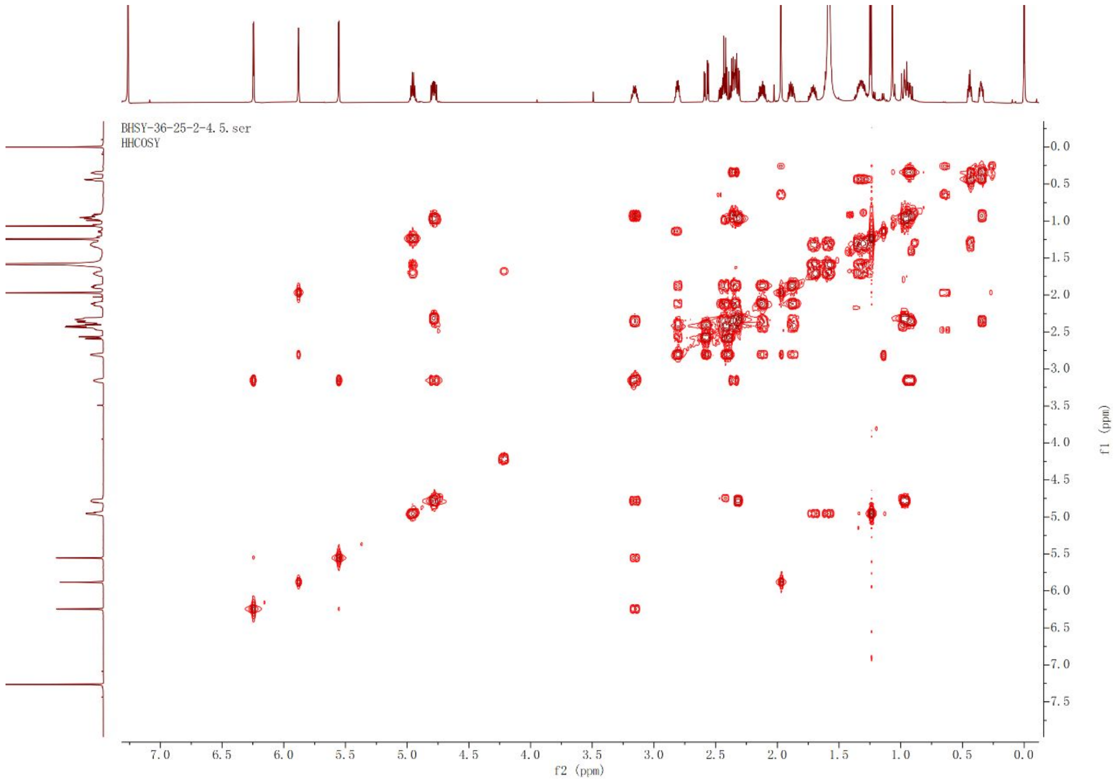


Figure S26. $^{1}H$-$^{1}H$ COSY spectrum (600 MHz) of carabrolate C (**3**) in $CDCl_3$

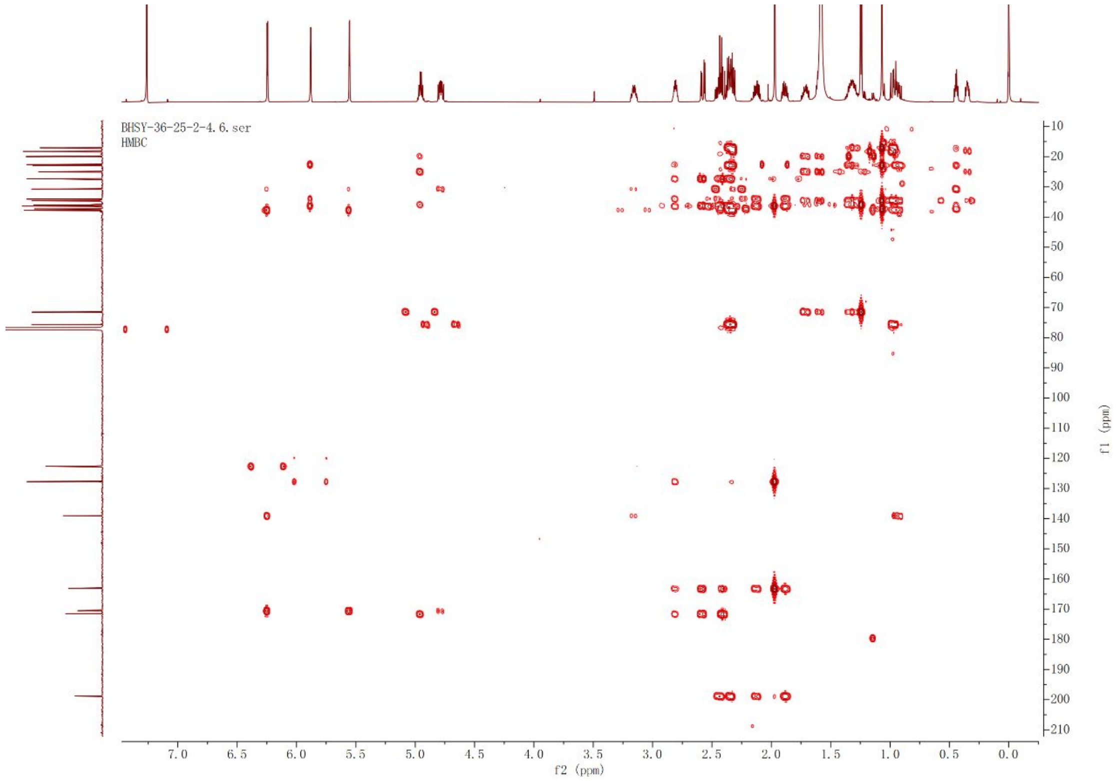


Figure S27. HMBC spectrum (600 MHz) of carabrolate C (**3**) in $CDCl_3$

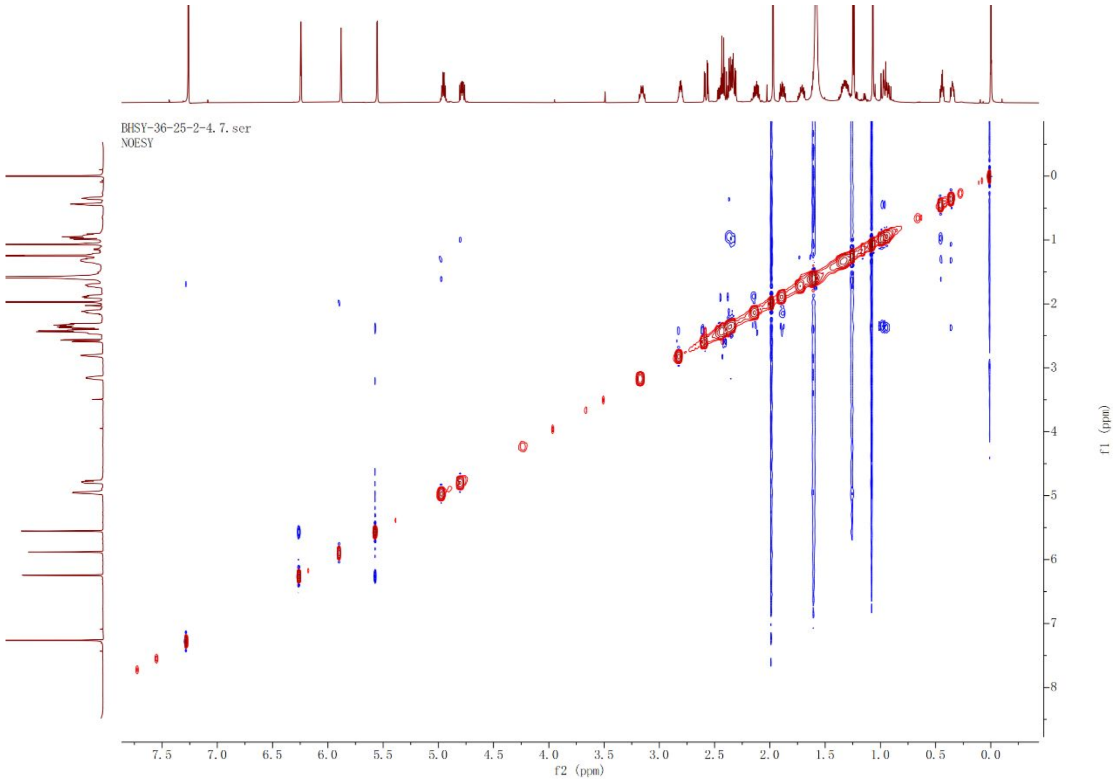


Figure S28. NOESY spectrum (600 MHz) of carabrolate C (**3**) in $CDCl_3$

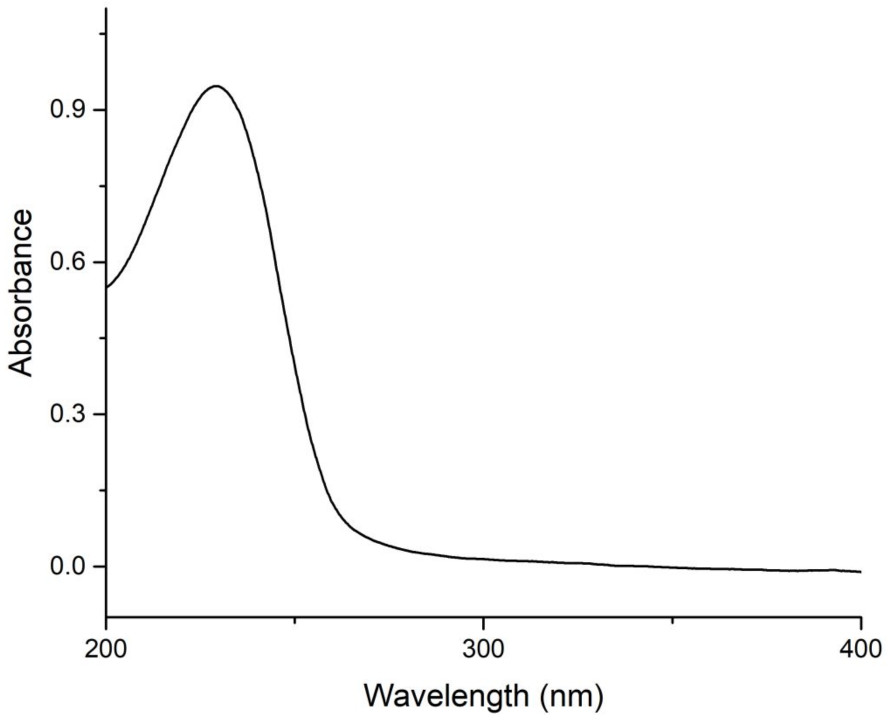


Figure S29. UV spectrum of carabrolate C (**3**) in MeOH

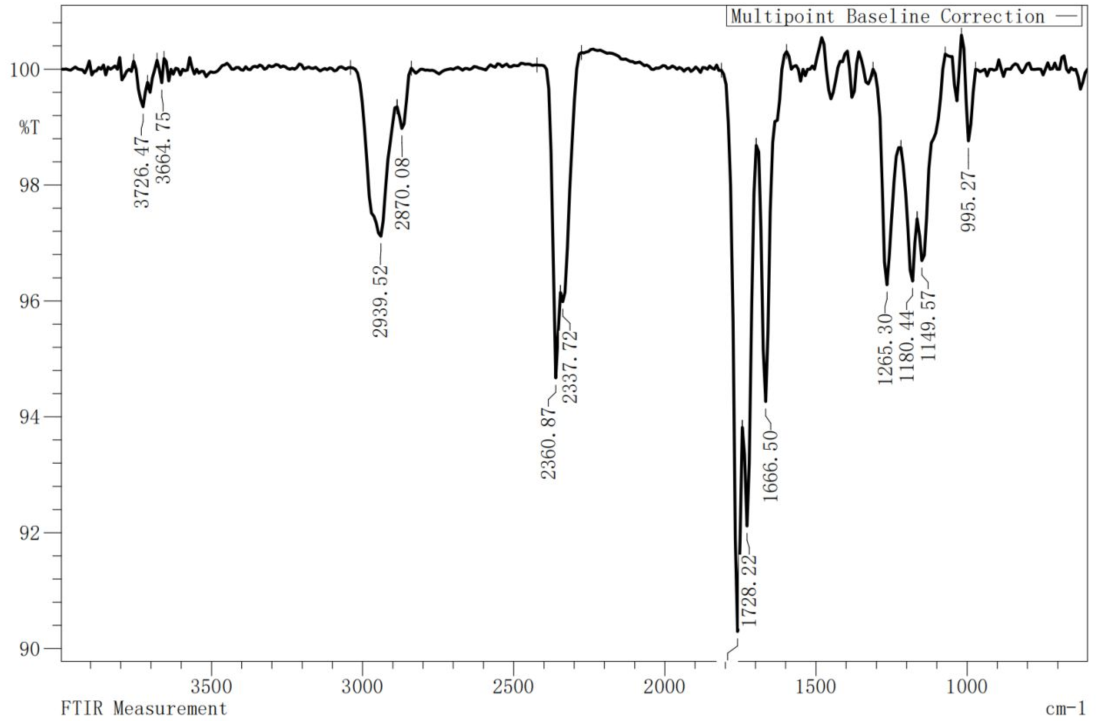


Figure S30. IR spectrum (film on KBr plate) of carabrolate C (**3**)

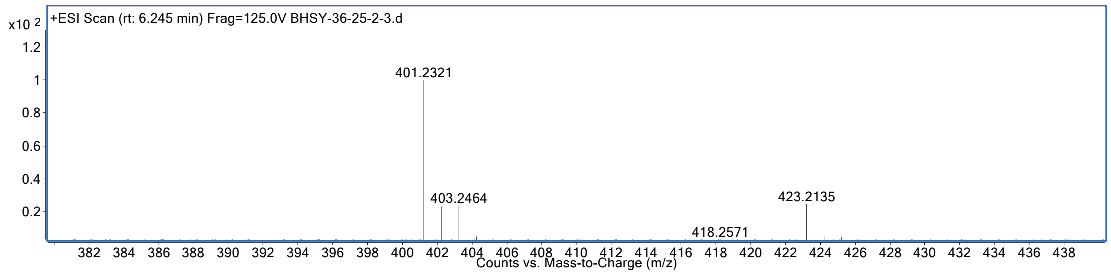


| Formula (M) | Score (MFG) | Mass | Mass (MFG) | m/z (Calc) | Diff (ppm) | Ion Formula | m/z |
|---|---|---|---|---|---|---|---|
| $C_{27}H_{40}O_7$ | 98.54 | 476.2765 | 476.2774 | 477.2847 | 1.85 | $C_{27}H_{41}O_7$ | 477.2838 |
| $C_{27}H_{40}O_7$ | 98.01 | 476.2764 | 476.2774 | 494.3112 | 2.16 | $C_{27}H_{44}NO_7$ | 494.3102 |

Figure S31. HRESIMS spectrum of carabrolate C (**3**)

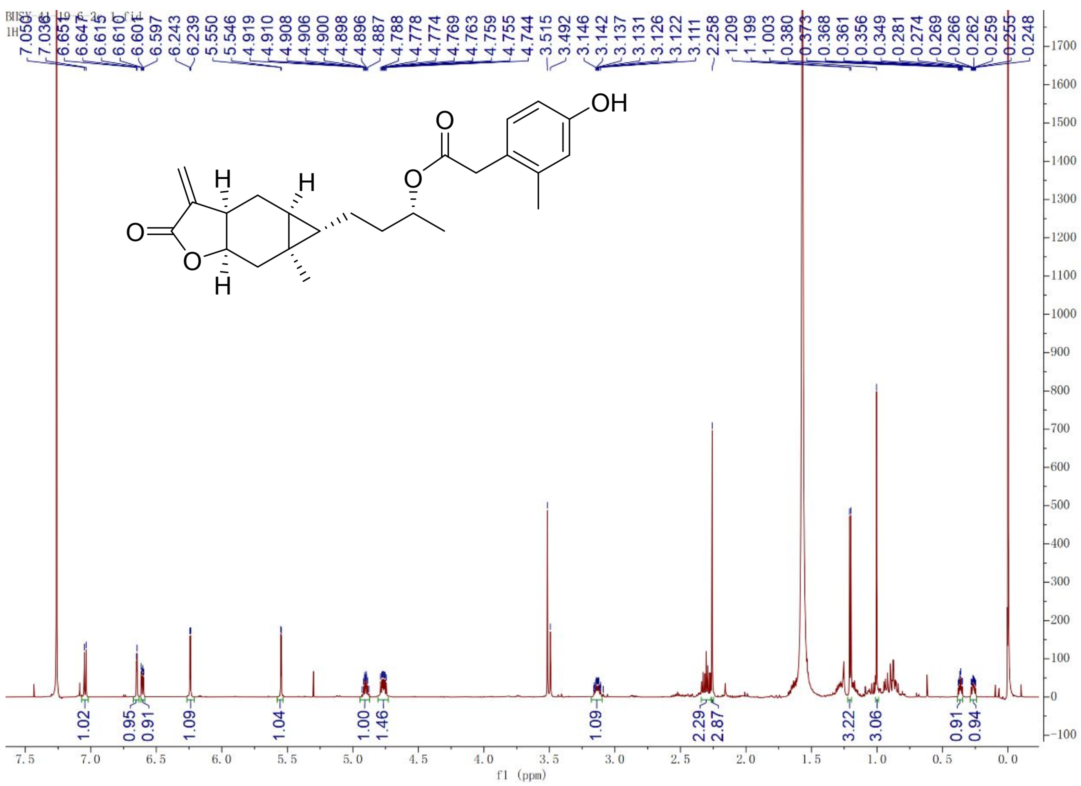


Figure S32. $^{1}$H NMR spectrum (600 MHz) of carabrolate D (**4**) in $CDCl_3$

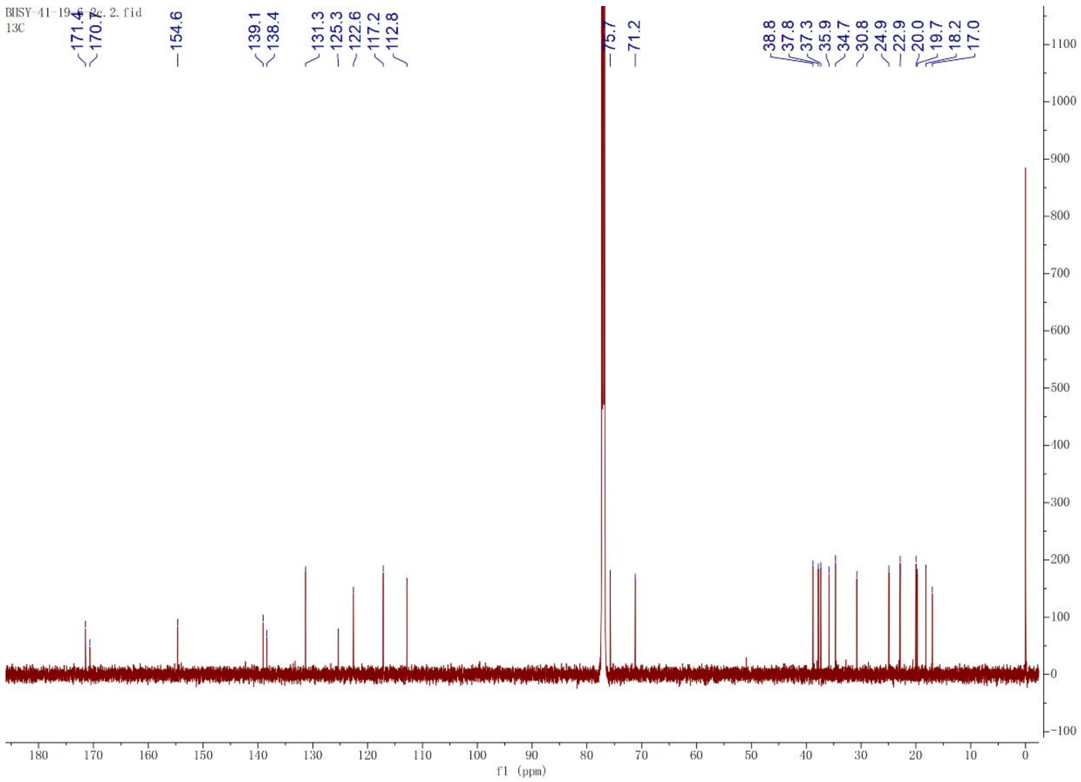


Figure S33. $^{13}$C NMR spectrum (150 MHz) of carabrolate D (**4**) in $CDCl_3$

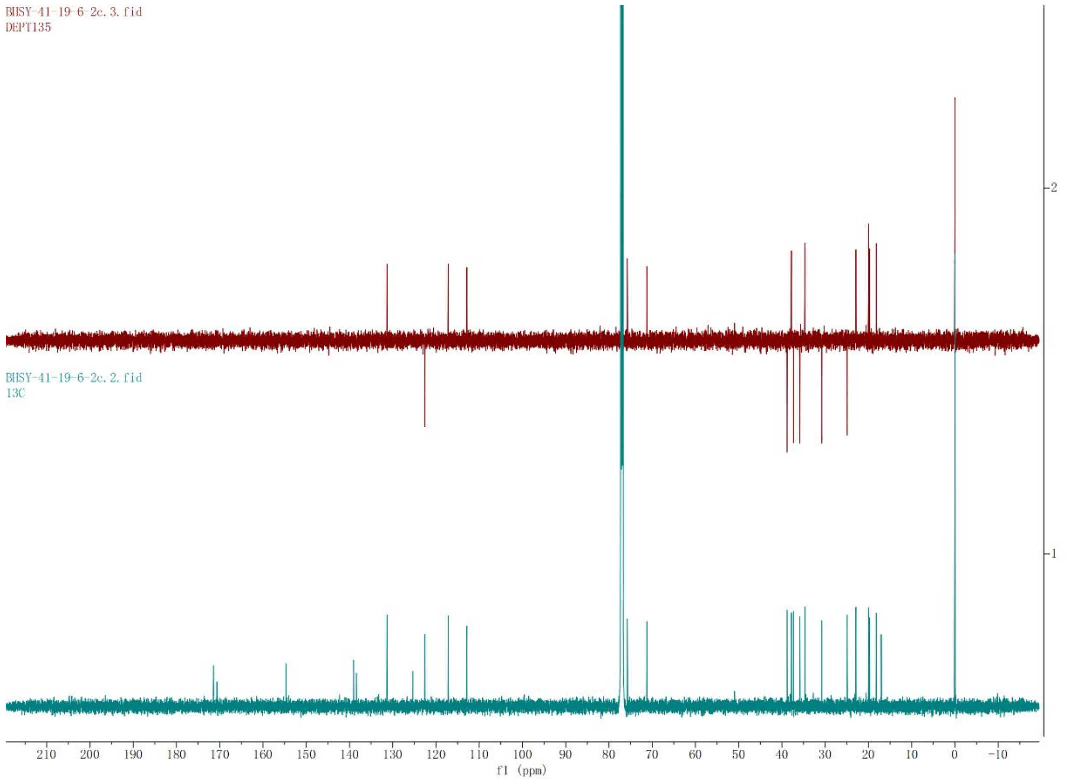


Figure S34. DEPT 135 spectrum (150 MHz) of carabrolate D (**4**) in $CDCl_3$

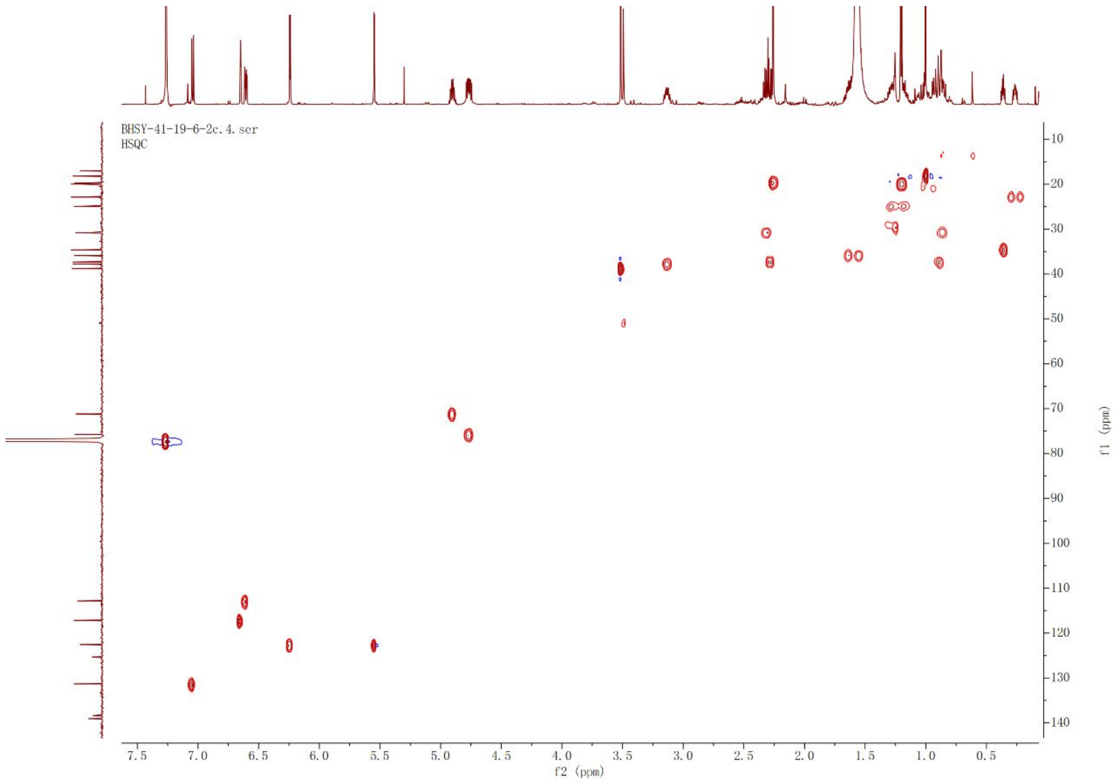


Figure S35. HSQC spectrum (600 MHz) of carabrolate D (**4**) in $CDCl_3$

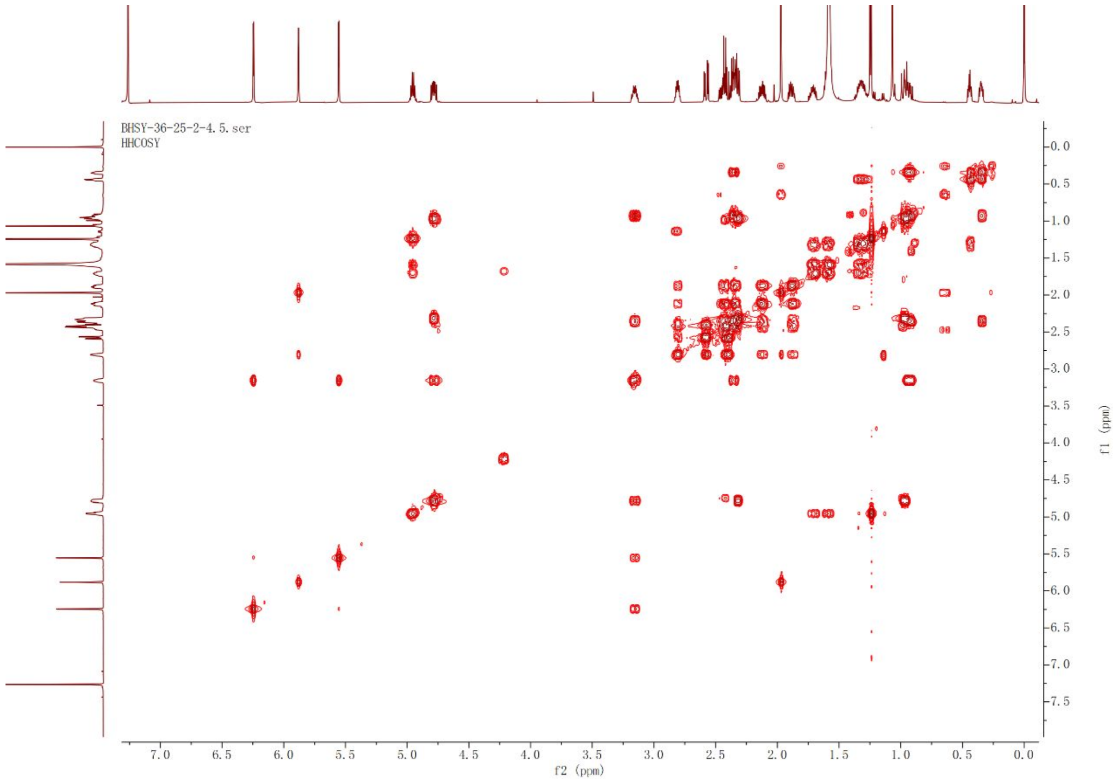


Figure S36. $^{1}H$-$^{1}H$ COSY spectrum (600 MHz) of carabrolate D (**4**) in $CDCl_3$

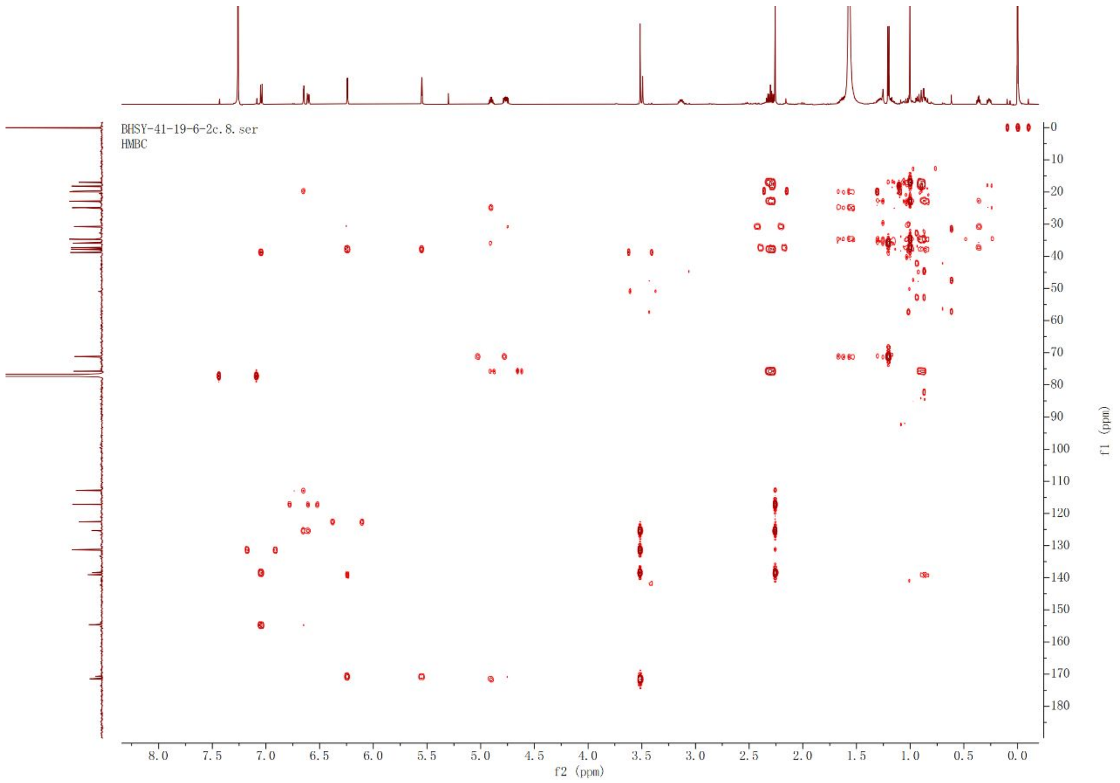


Figure S37. HMBC spectrum (600 MHz) of carabrolate D (**4**) in $CDCl_3$

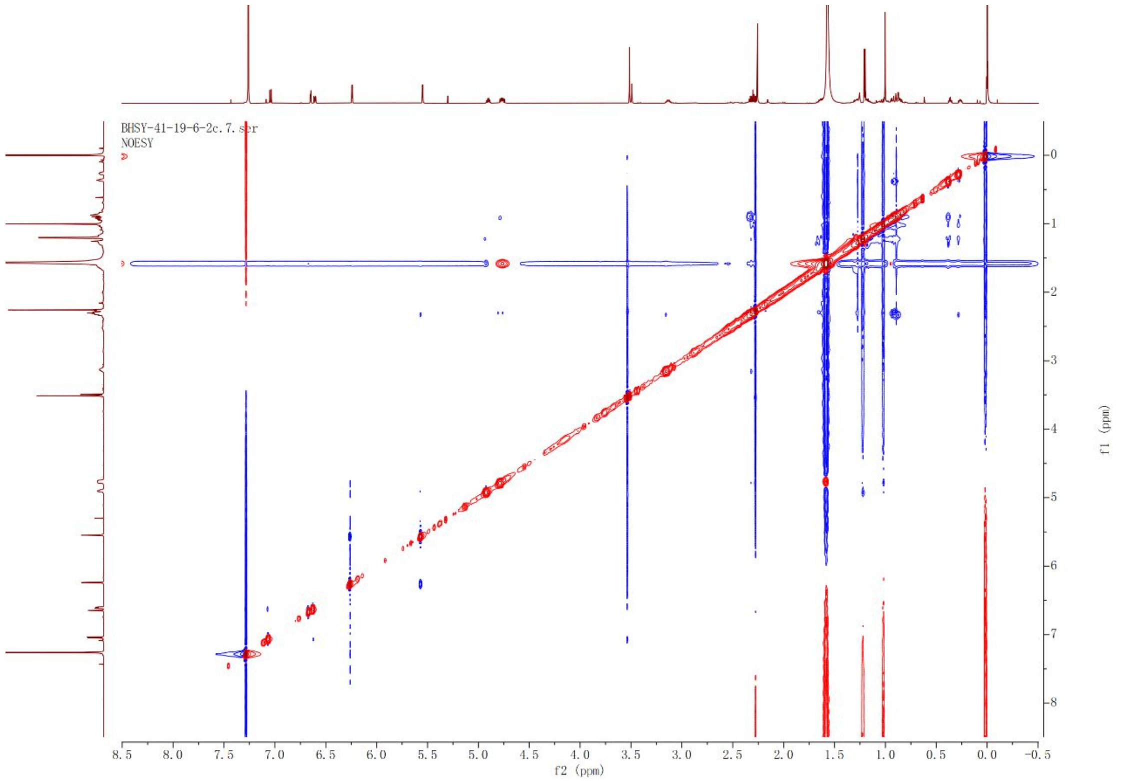


Figure S38. NOESY spectrum (600 MHz) of carabrolate D (**4**) in $CDCl_3$

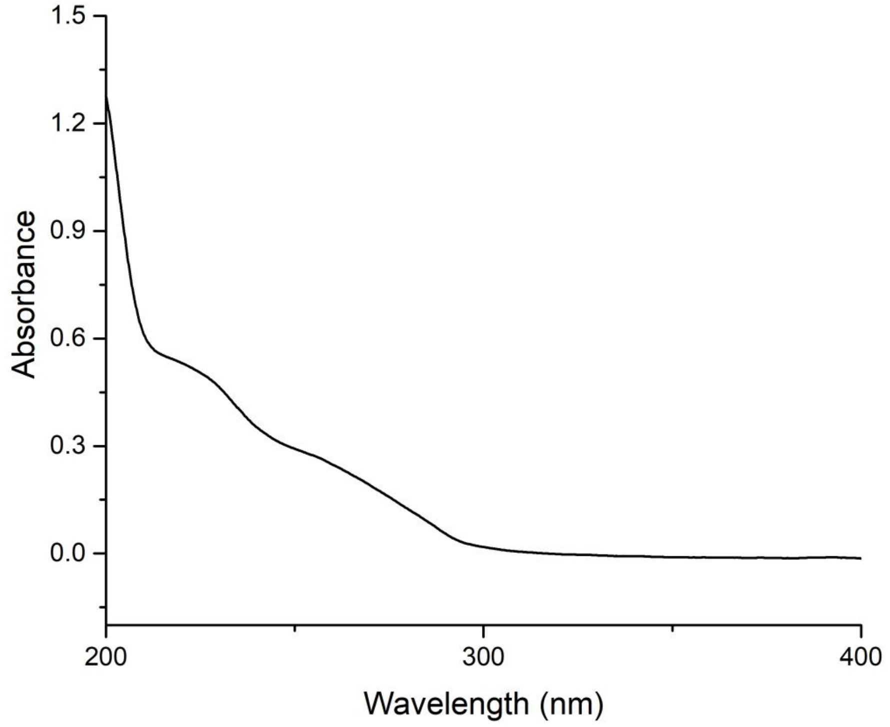


Figure S39. UV spectrum of carabrolate D (**4**) in MeOH

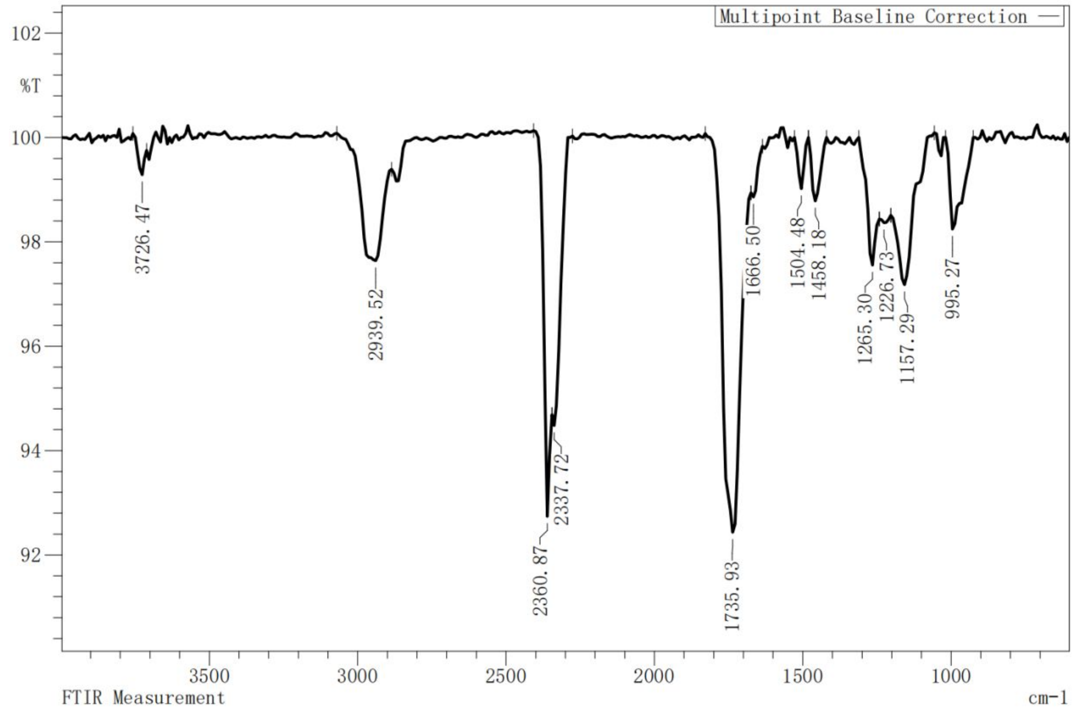


Figure S40. IR spectrum (film on KBr plate) of carabrolate D (**4**)

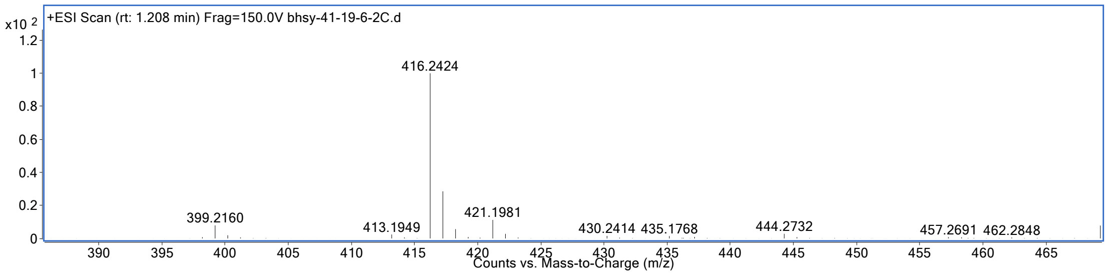


| Formula (M) | Score (MFG) | Mass | Mass (MFG) | m/z (Calc) | Diff (ppm) | Ion Formula | m/z |
|---|---|---|---|---|---|---|---|
| $C_{24}H_{30}O_5$ | 99.09 | 398.2087 | 398.2093 | 399.2166 | 1.51 | $C_{24}H_{31}O_5$ | 399.216 |
| $C_{24}H_{30}O_5$ | 98.59 | 398.2086 | 398.2093 | 416.2431 | 1.88 | $C_{24}H_{34}NO_5$ | 416.2424 |

Figure S41. HRESIMS spectrum of carabrolate D (**4**)

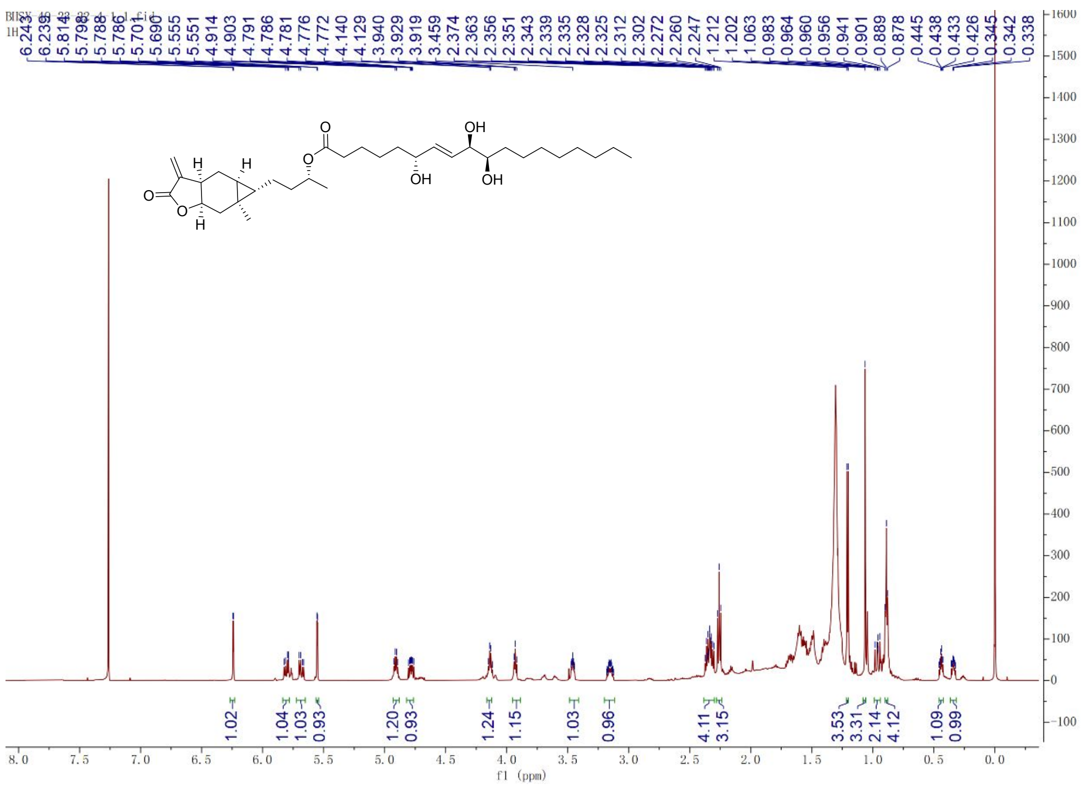

Figure S42. $^{1}H$ NMR spectrum (600 MHz) of carabrolate E (**5**) in $CDCl_3$

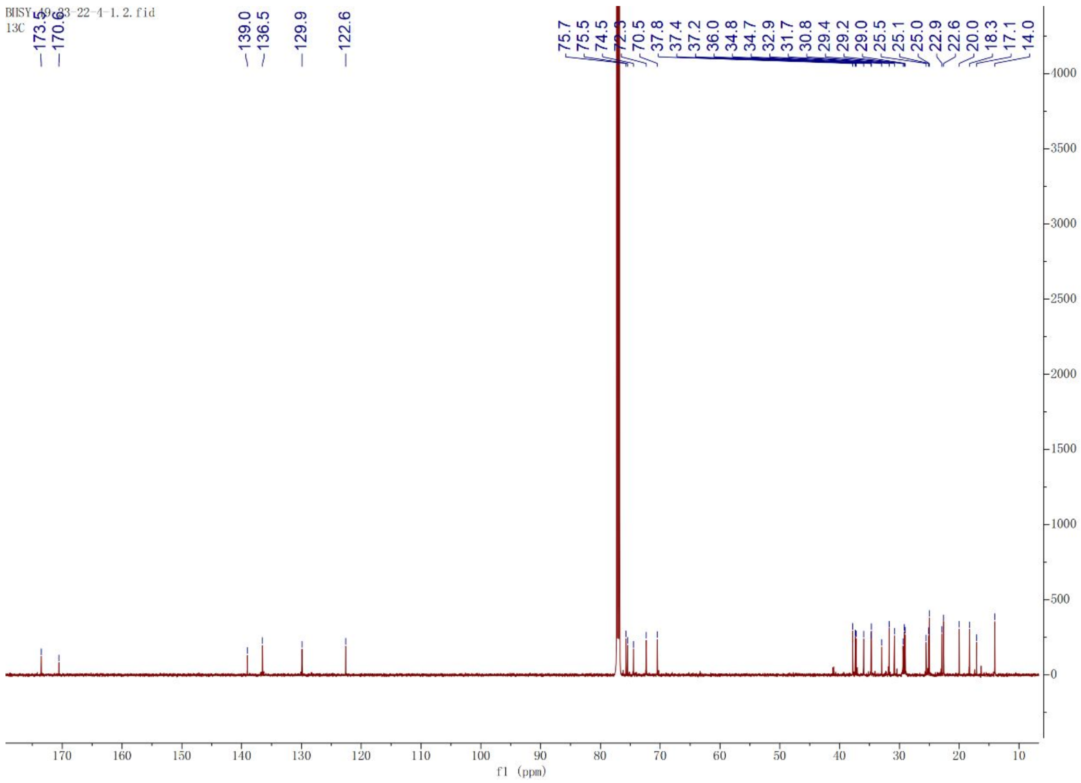

Figure S43. $^{13}C$ NMR spectrum (150 MHz) of carabrolate E (**5**) in $CDCl_3$

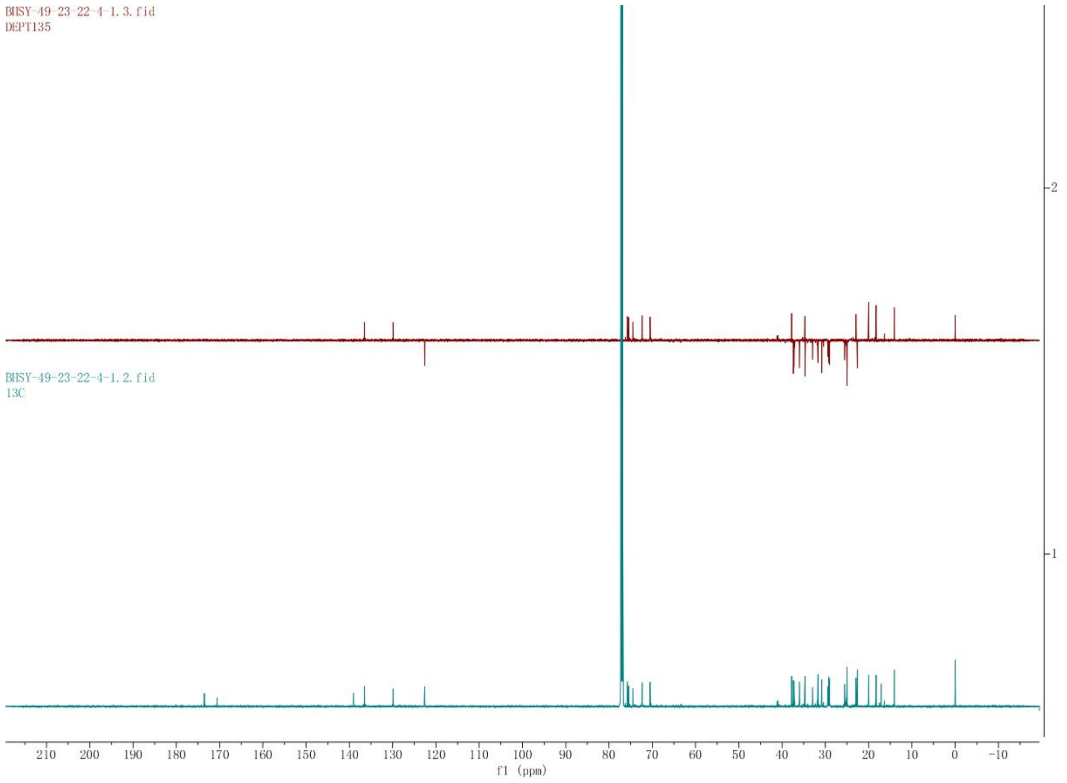


Figure S44. DEPT 135 spectrum (150 MHz) of carabrolate E (**5**) in $CDCl_3$

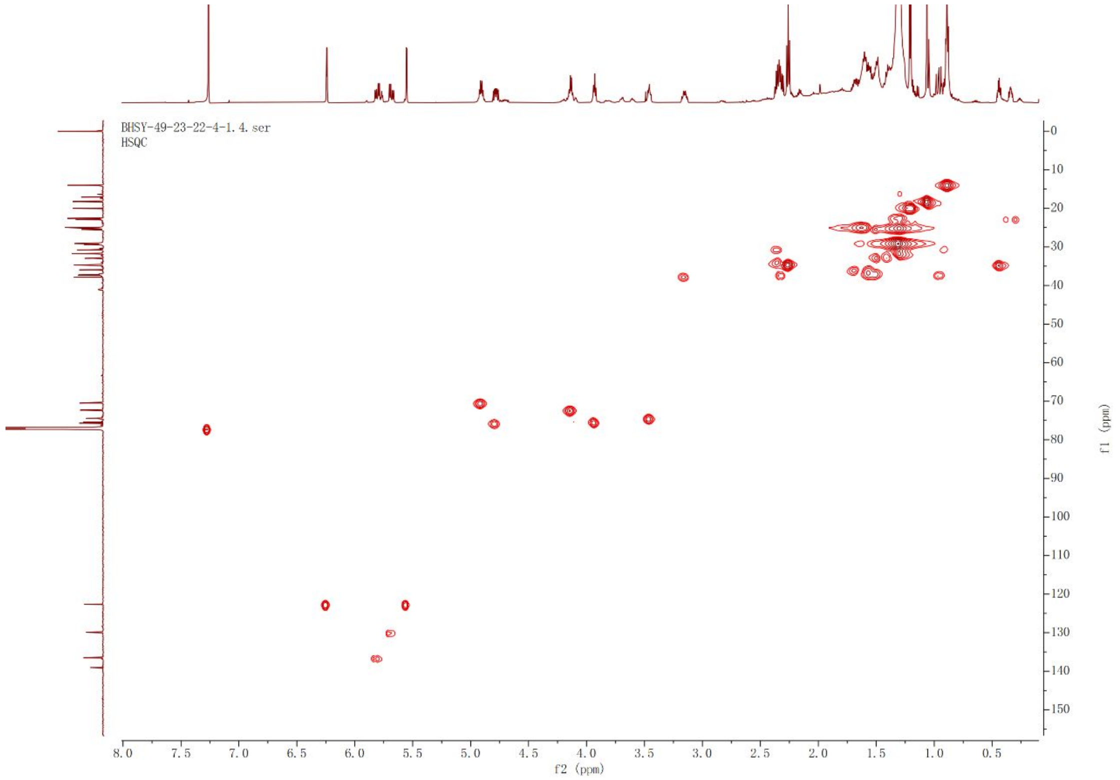


Figure S45. HSQC spectrum (600 MHz) of carabrolate E (**5**) in $CDCl_3$

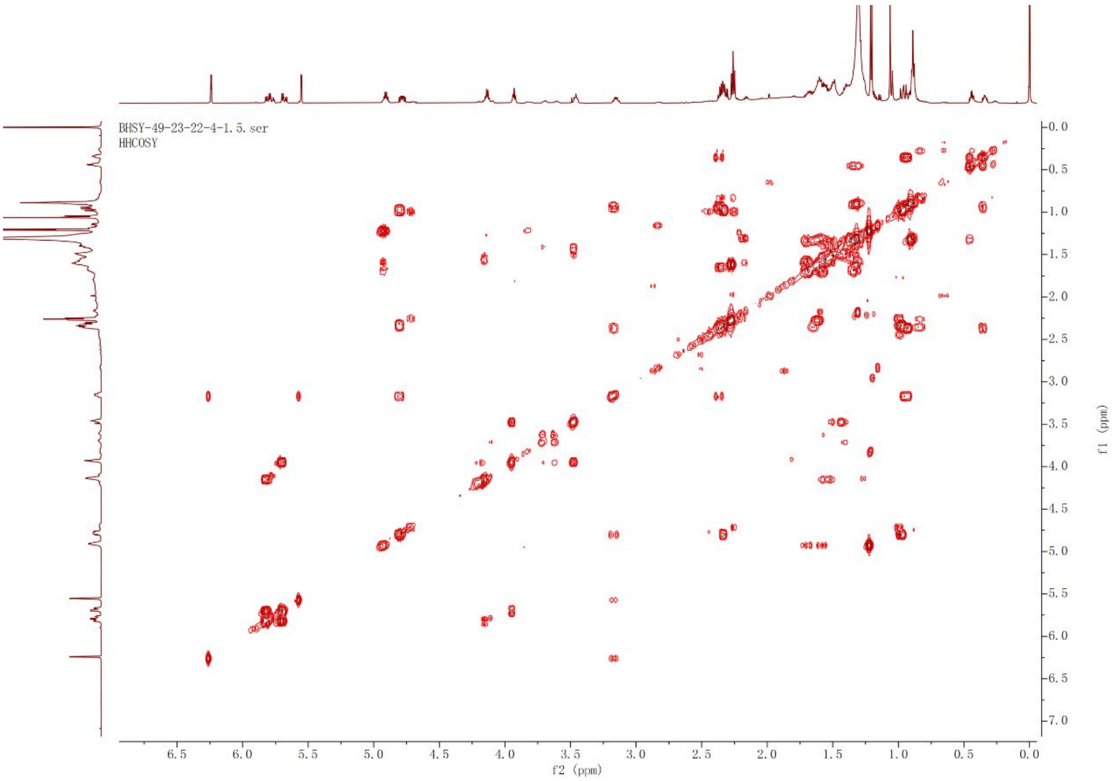


Figure S46. $^{1}H$-$^{1}H$ COSY spectrum (600 MHz) of carabrolate E (**5**) in $CDCl_3$

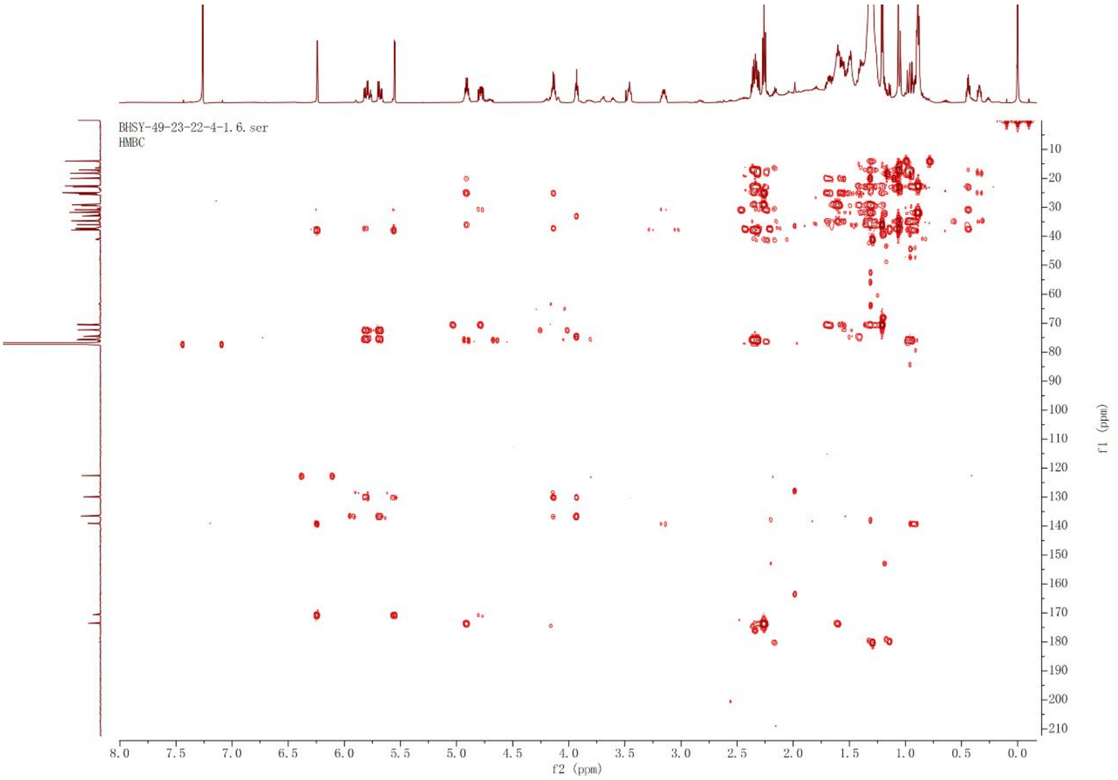


Figure S47. HMBC spectrum (600 MHz) of carabrolate E (**5**) in $CDCl_3$

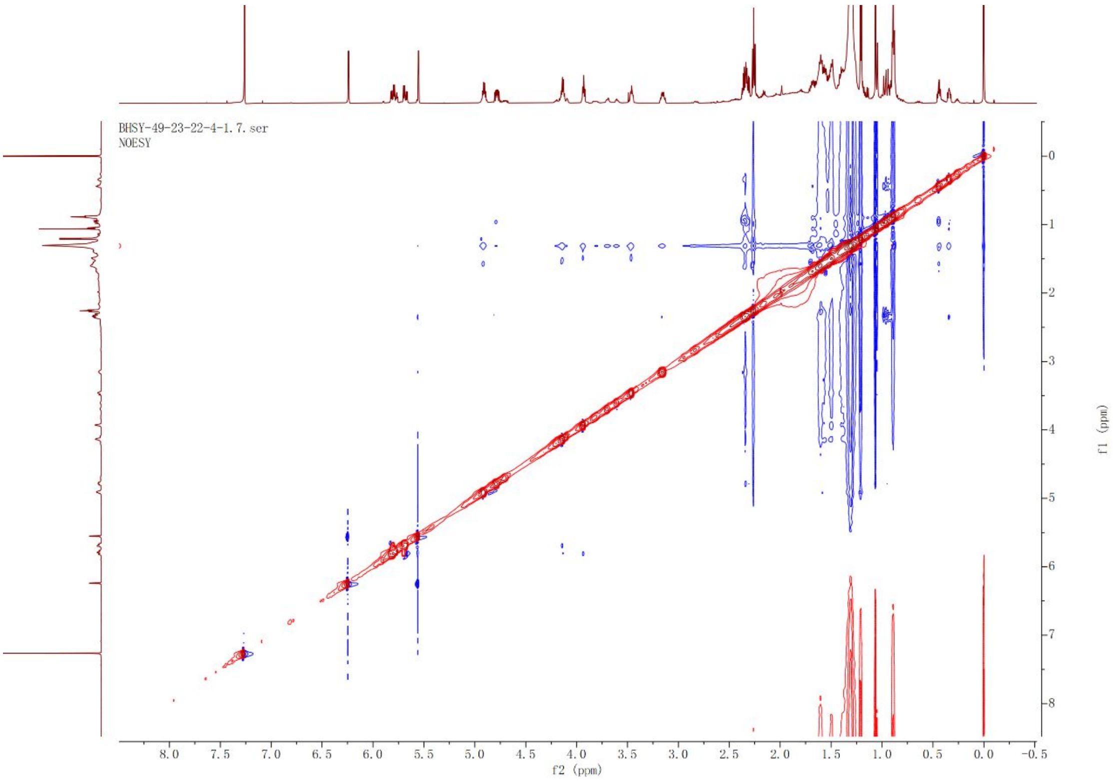


Figure S48. NOESY spectrum (600 MHz) of carabrolate E (**5**) in $CDCl_3$

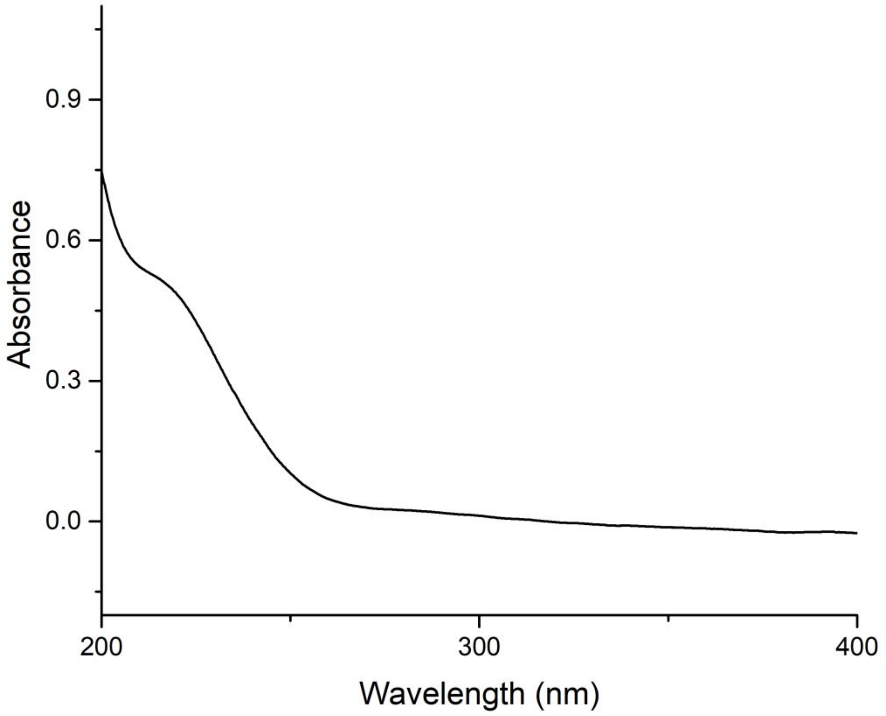


Figure S49. UV spectrum of carabrolate E (**5**) in MeOH

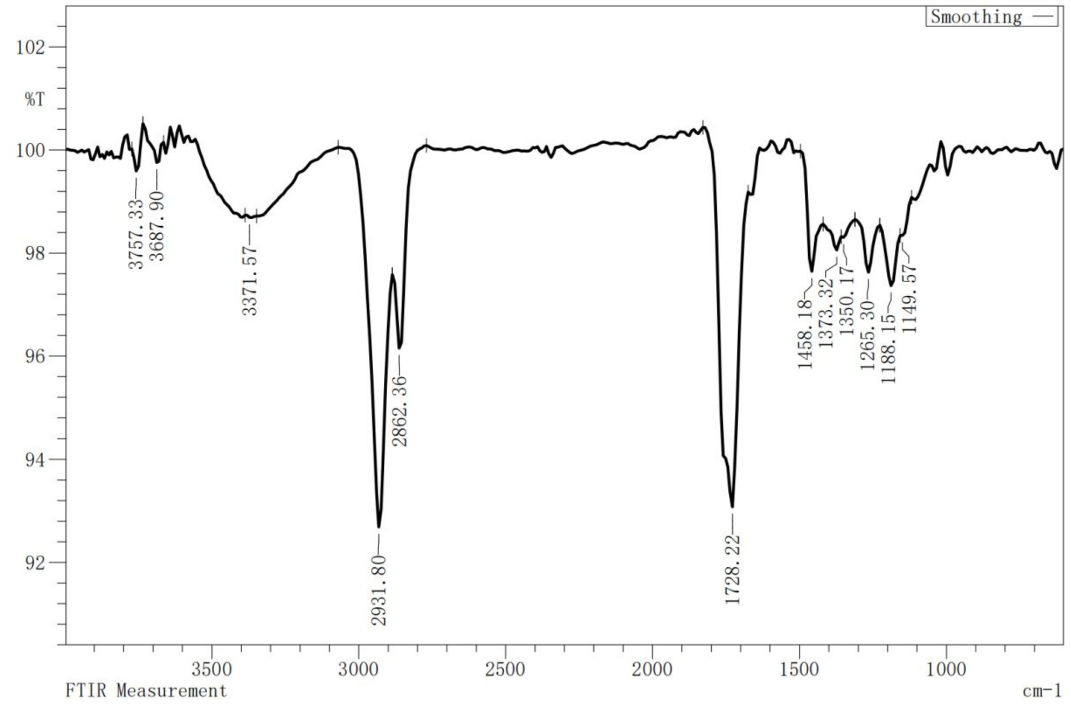


Figure S50. IR spectrum (film on KBr plate) of carabrolate E (**5**)

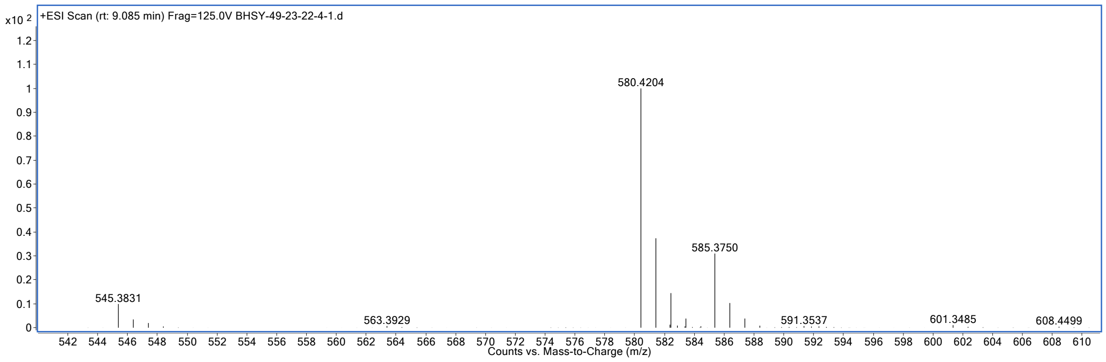


| Formula (M) | Score (MFG) | Mass | Mass (MFG) | m/z (Calc) | Diff (ppm) | Ion Formula | m/z |
|---|---|---|---|---|---|---|---|
| $C_{33}H_{54}O_7$ | 99.77 | 562.3866 | 562.387 | 580.4208 | 0.68 | $C_{30}H_{58}NO_7$ | 580.4204 |
| $C_{33}H_{54}O_7$ | 97.86 | 562.3858 | 562.387 | 585.3762 | 2.09 | $C_{30}H_{54}NaO_7$ | 585.375 |

Figure S51. HRESIMS spectrum of carabrolate E (**5**)

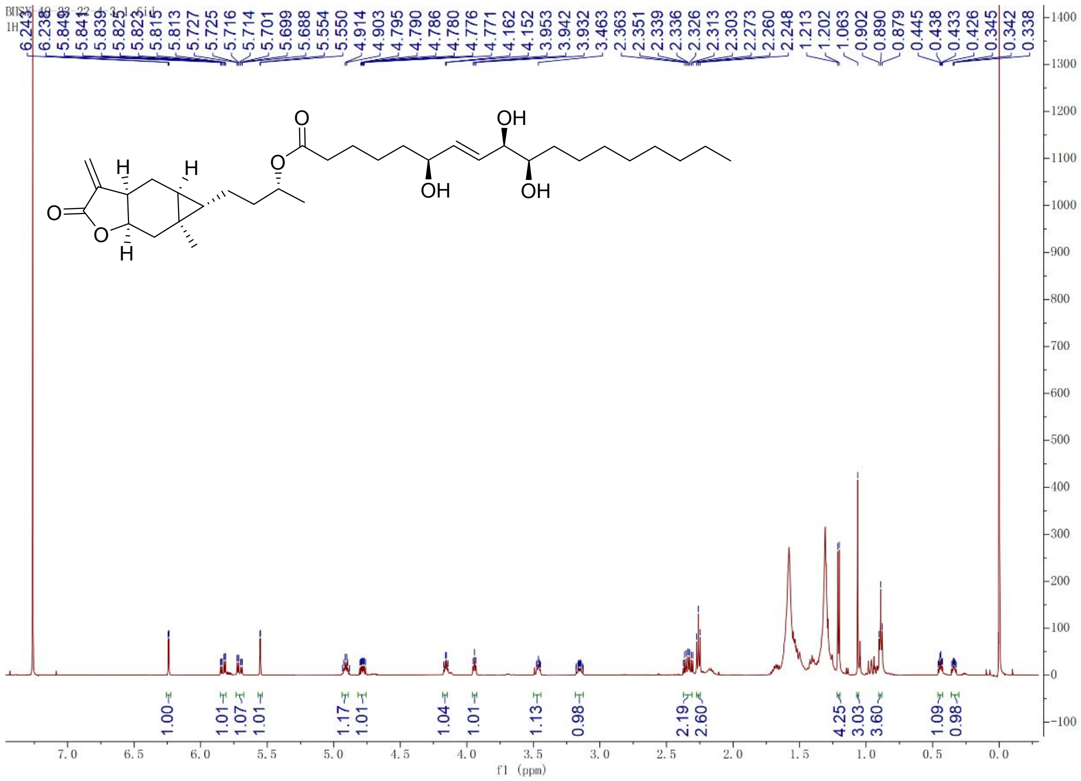


Figure S52. $^{1}$H NMR spectrum (600 MHz) of carabrolate F (**6**) in $CDCl_3$

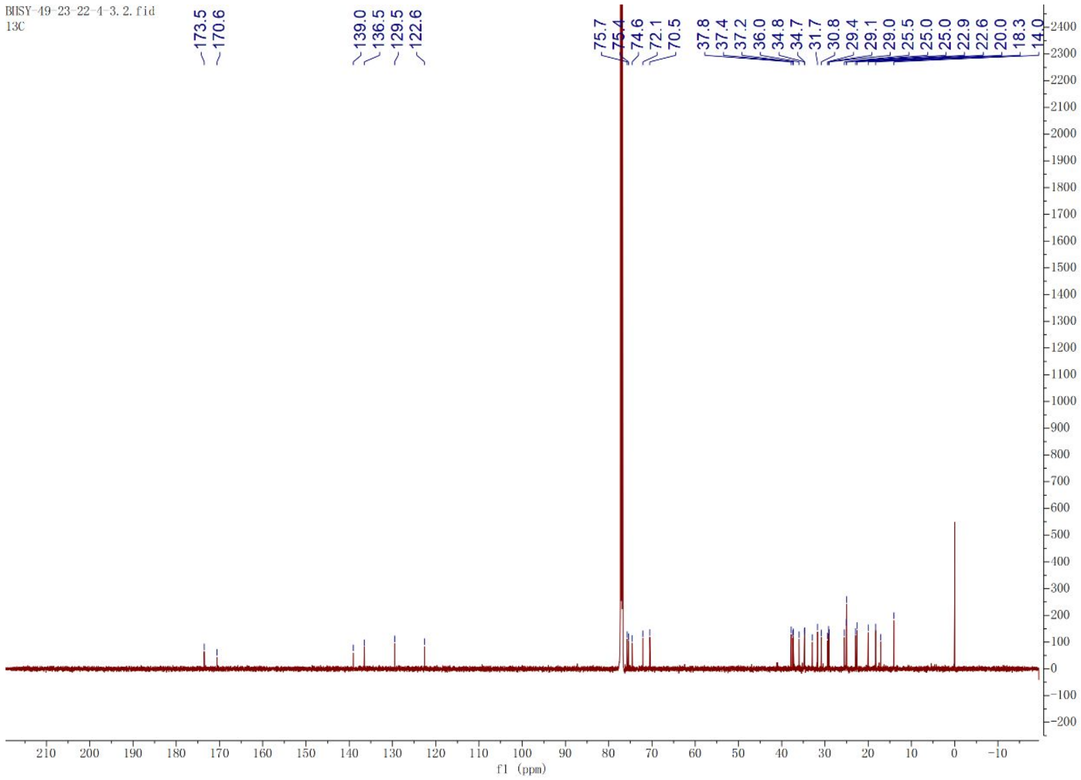


Figure S53. $^{13}$C NMR spectrum (150 MHz) of carabrolate F (**6**) in $CDCl_3$

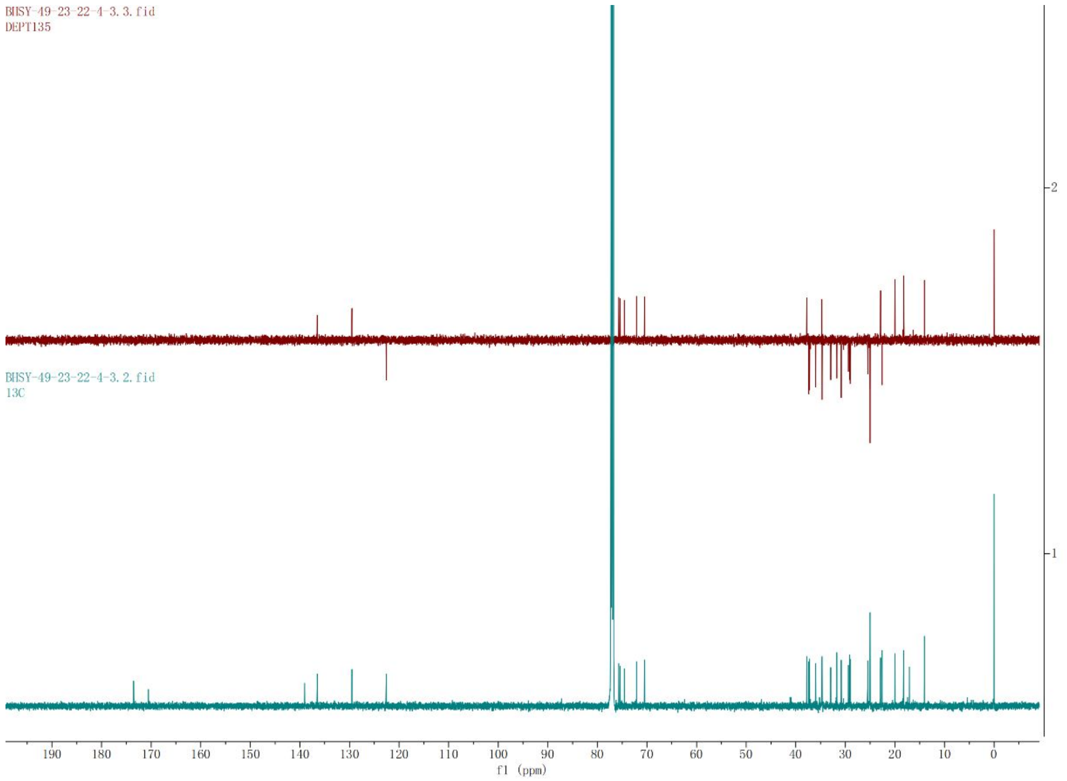


Figure S54. DEPT 135 spectrum (150 MHz) of carabrolate F (**6**) in $CDCl_3$

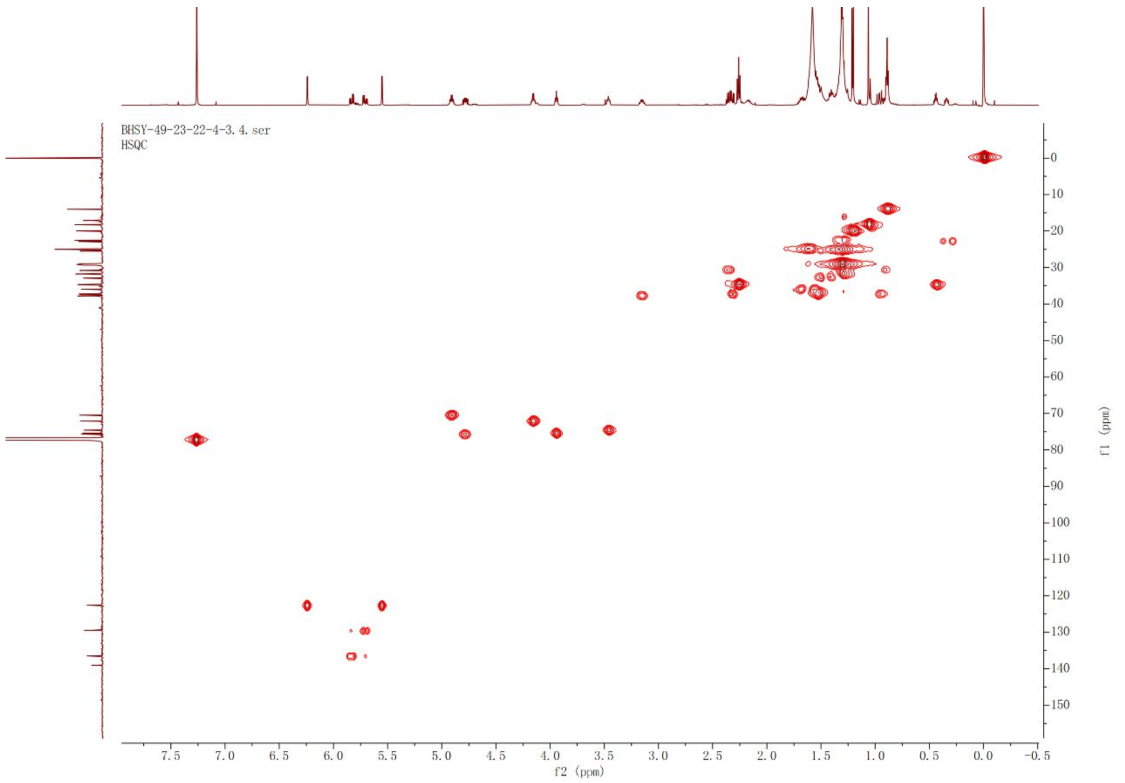


Figure S55. HSQC spectrum (600 MHz) of carabrolate F (**6**) in $CDCl_3$

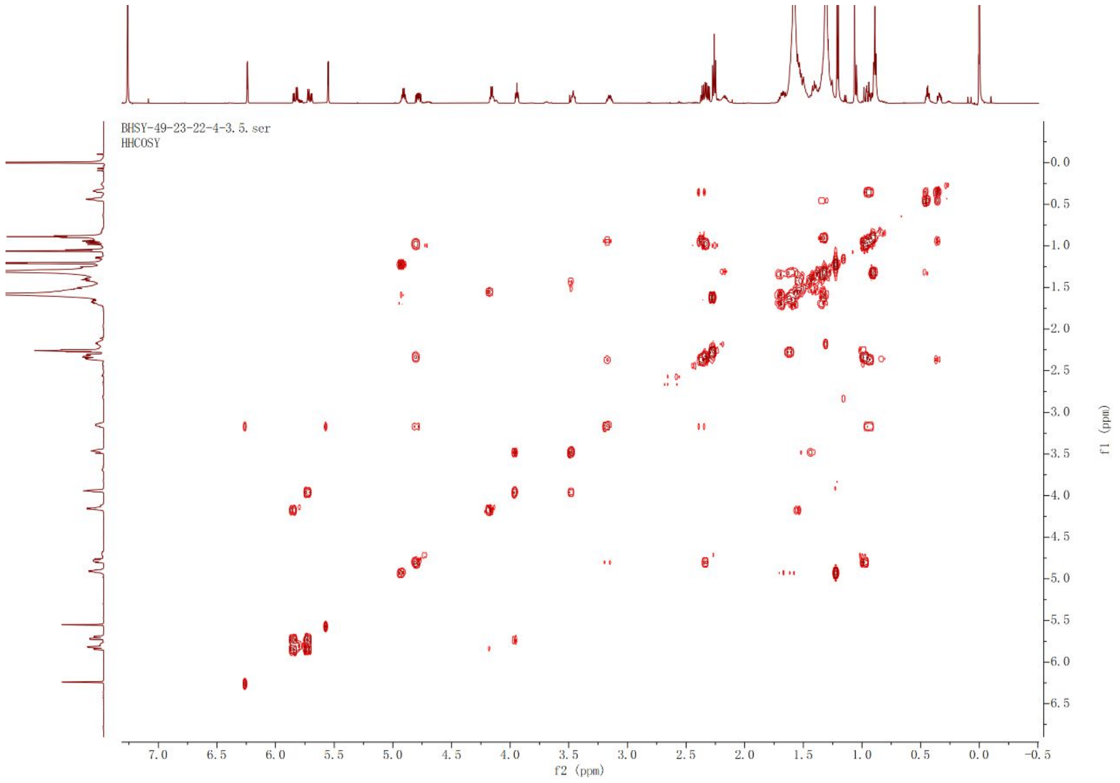


Figure S56. $^{1}H$-$^{1}H$ COSY spectrum (600 MHz) of carabrolate F (**6**) in $CDCl_3$

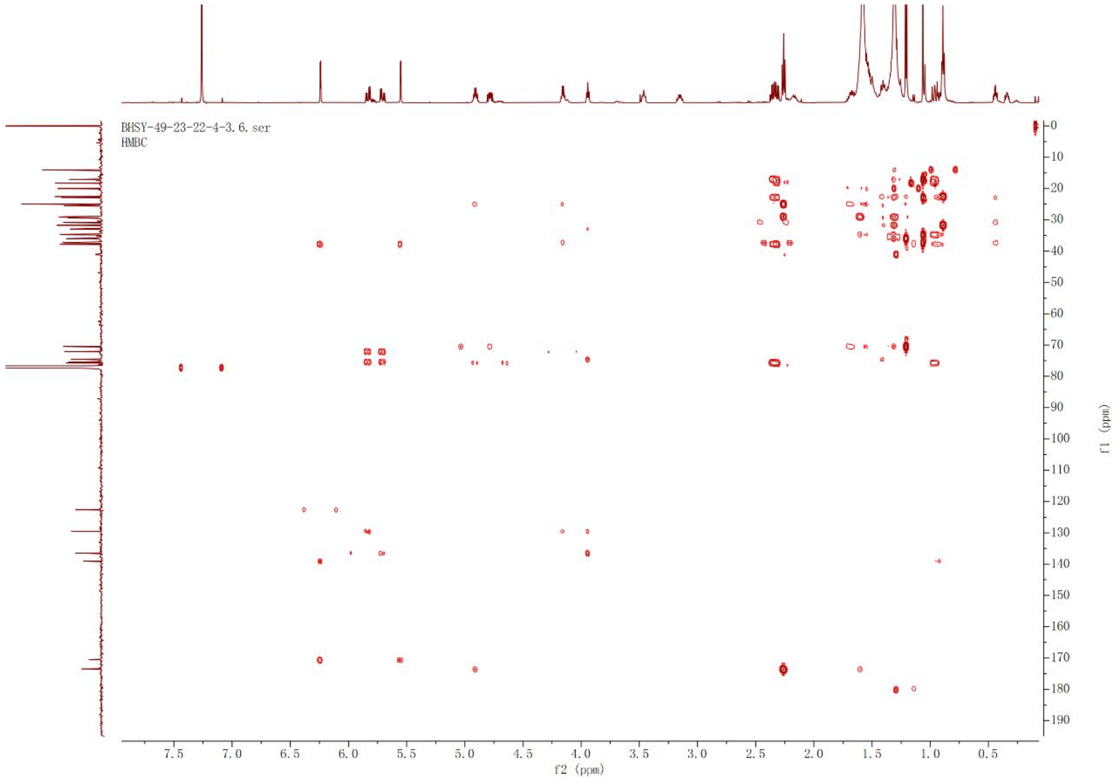


Figure S57. HMBC spectrum (600 MHz) of carabrolate F (**6**) in $CDCl_3$

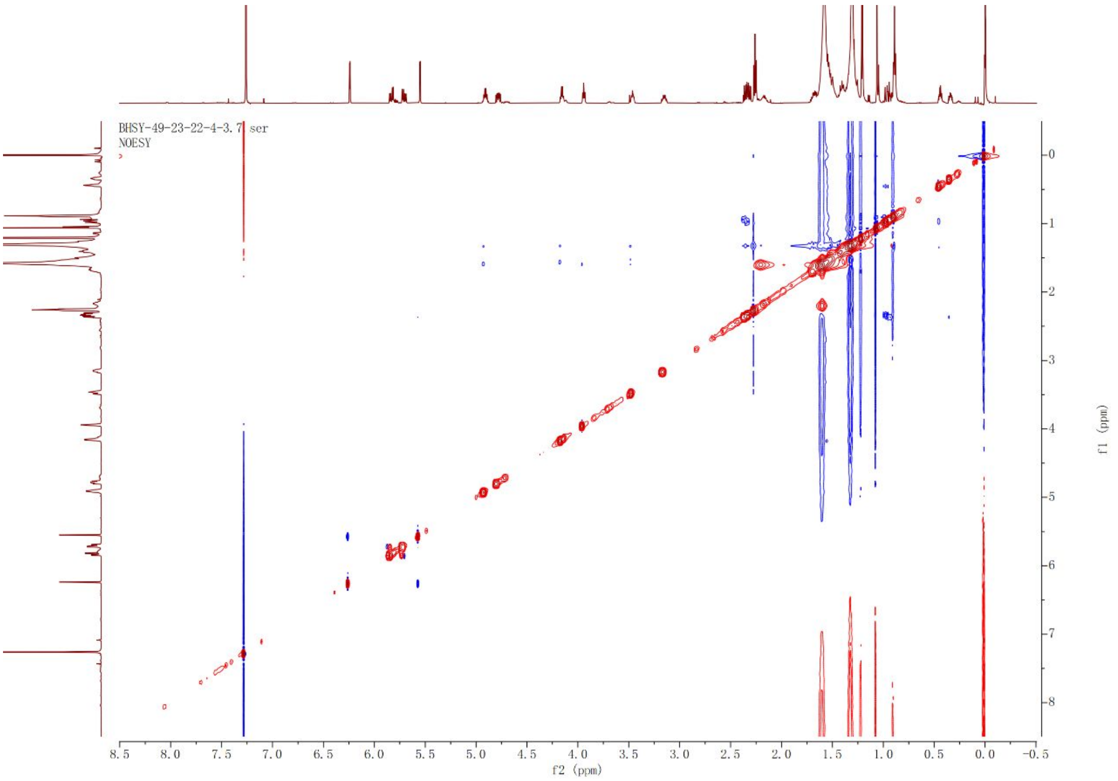


Figure S58. NOESY spectrum (600 MHz) of carabrolate F (**6**) in $CDCl_3$

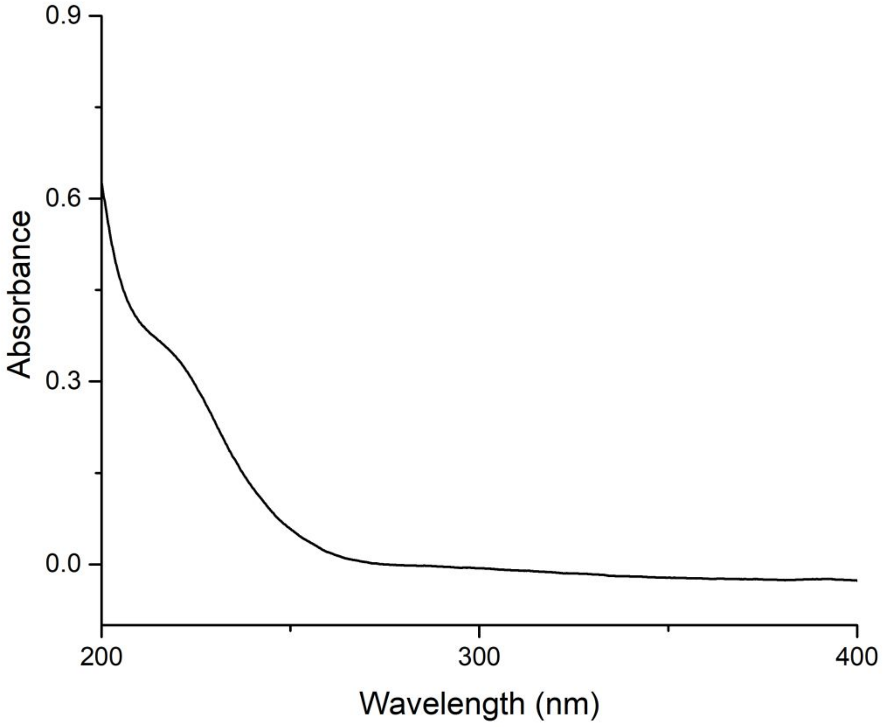


Figure S59. UV spectrum of carabrolate F (**6**) in MeOH

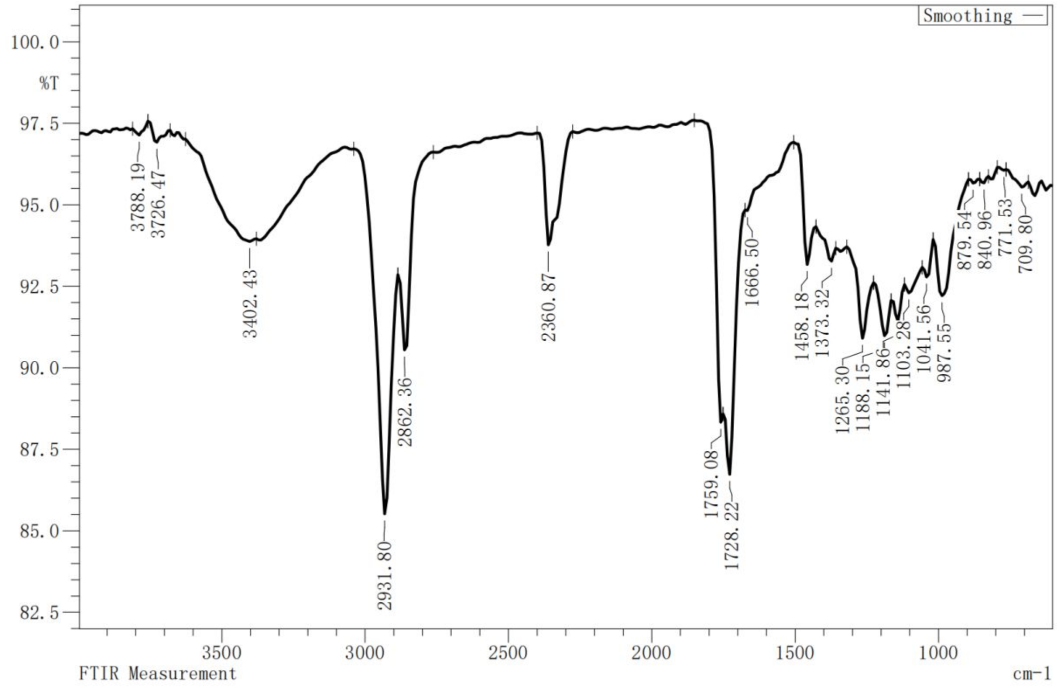

Figure S60. IR spectrum (film on KBr plate) of carabrolate F (**6**)

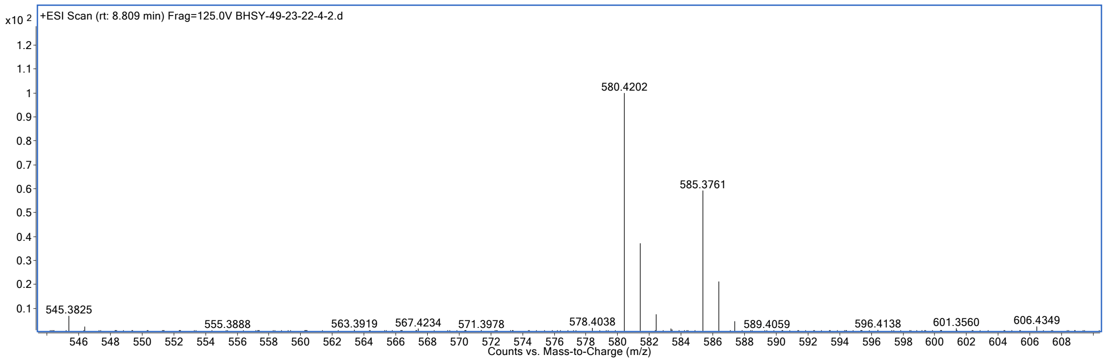

| Formula (M) | Score (MFG) | Mass | Mass (MFG) | m/z (Calc) | Diff (ppm) | Ion Formula | m/z |
|---|---|---|---|---|---|---|---|
| $C_{33}H_{54}O_7$ | 99.47 | 562.3864 | 562.387 | 580.4208 | 1.03 | $C_{30}H_{58}NO_7$ | 580.4202 |
| $C_{33}H_{54}O_7$ | 99.99 | 562.3869 | 562.387 | 585.3762 | 0.13 | $C_{30}H_{54}NaO_7$ | 585.3761 |

Figure S61. HRESIMS spectrum of carabrolate F (**6**)

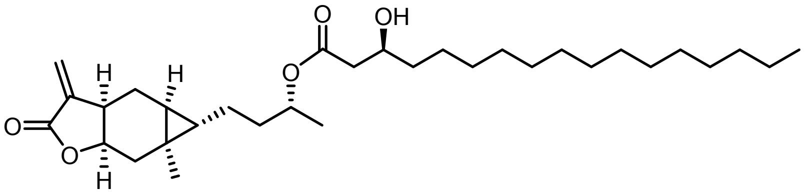

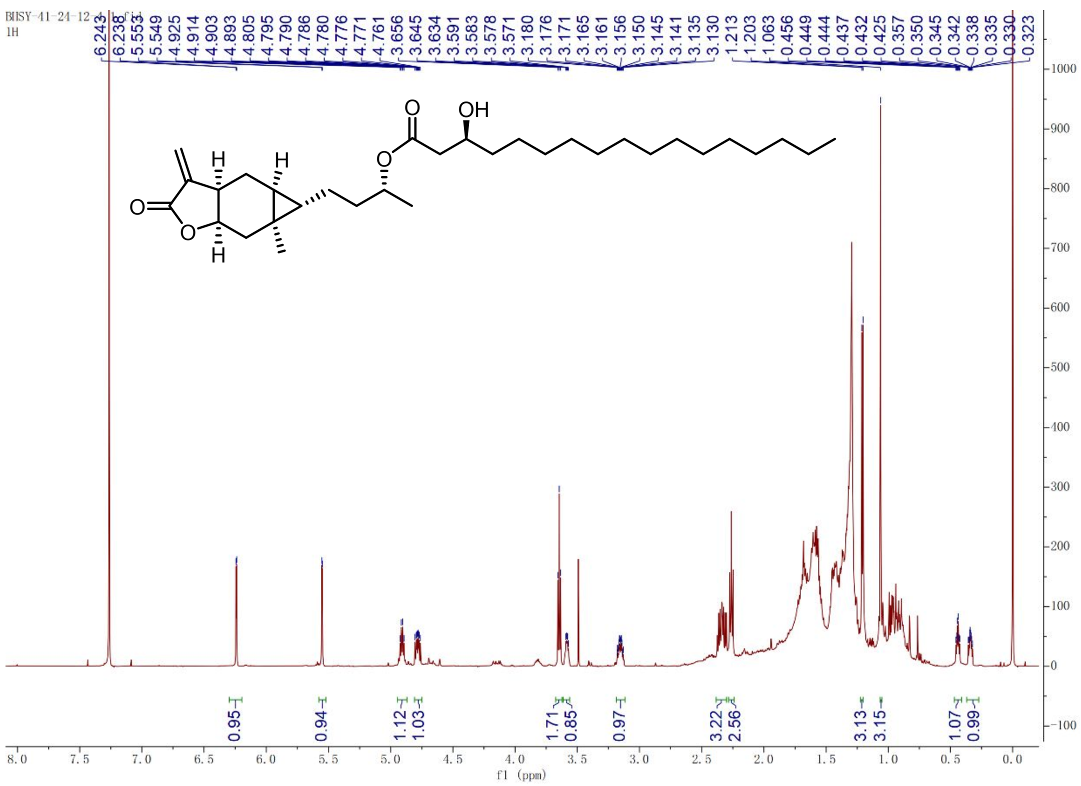


Figure S62. $^{1}$H NMR spectrum (600 MHz) of carabrolate G (**7**) in $CDCl_3$

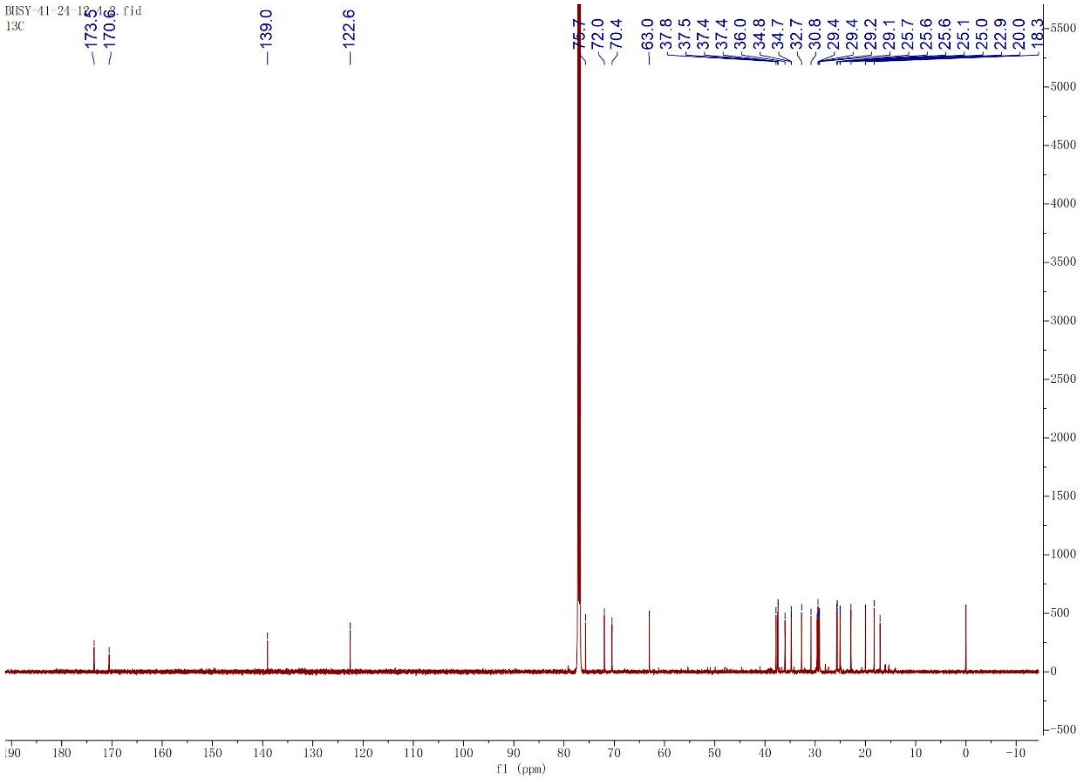


Figure S63. $^{13}$C NMR spectrum (150 MHz) of carabrolate G (**7**) in $CDCl_3$

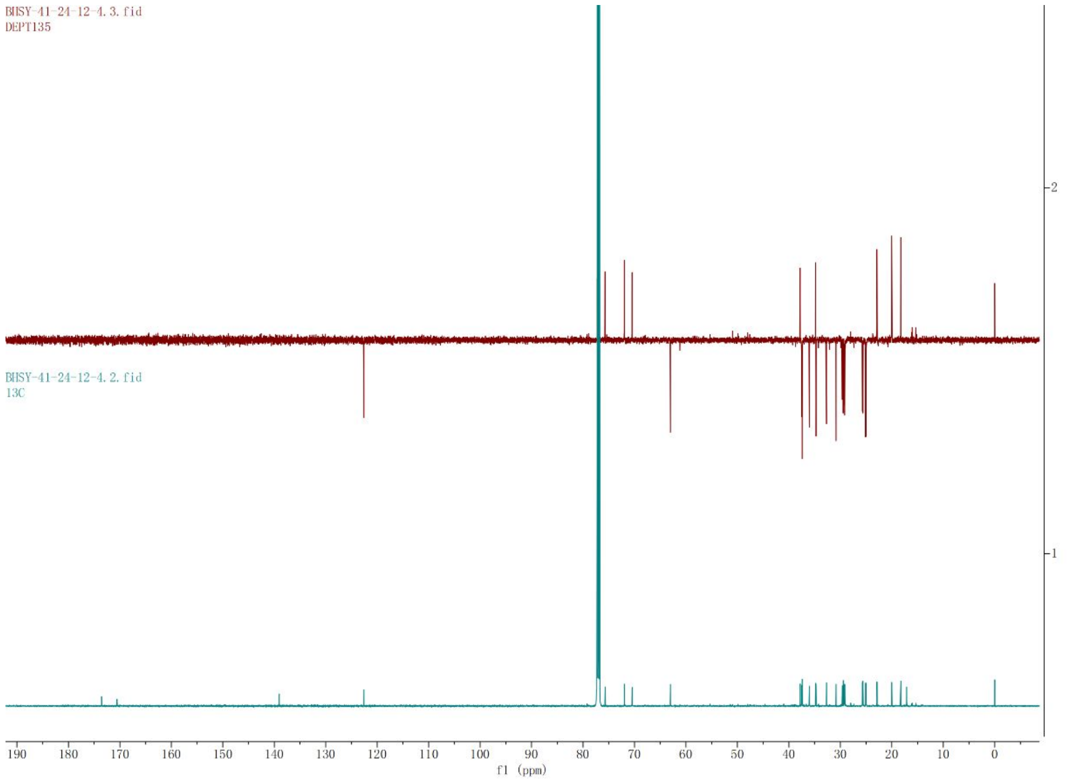


Figure S64. DEPT 135 spectrum (150 MHz) of carabrolate G (**7**) in $CDCl_3$

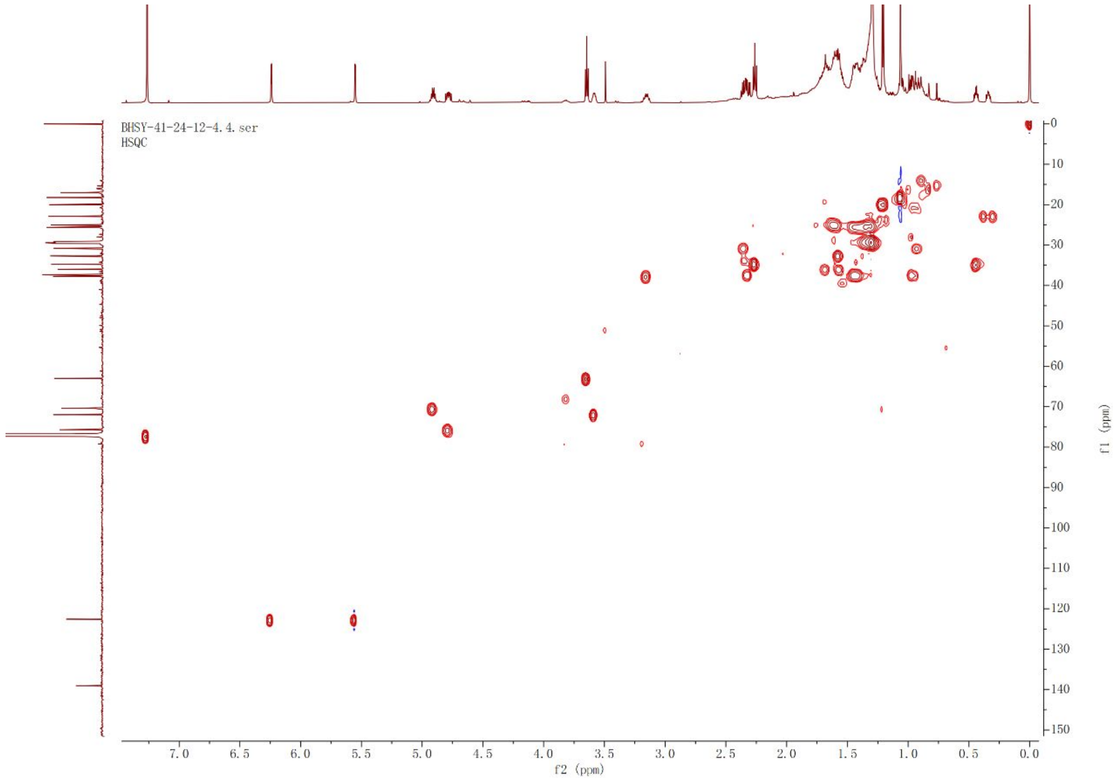


Figure S65. HSQC spectrum (600 MHz) of carabrolate G (**7**) in $CDCl_3$

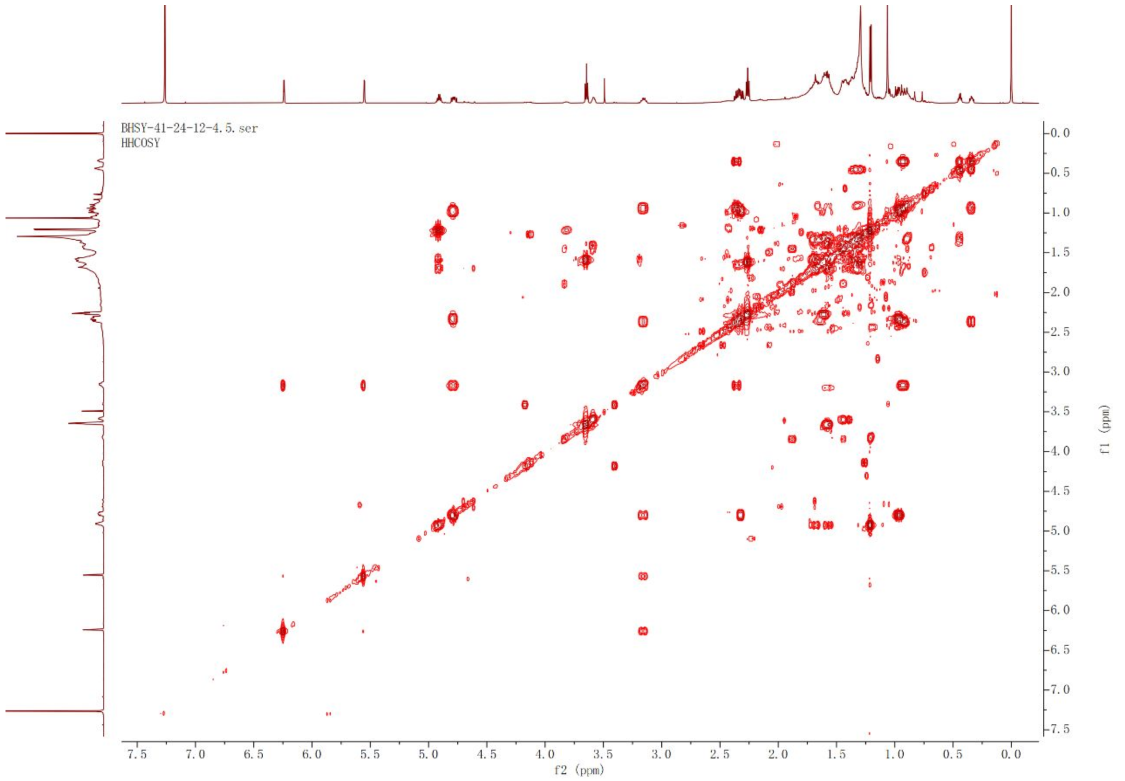


Figure S66. $^1H$-$^1H$ COSY spectrum (600 MHz) of carabrolate G (**7**) in $CDCl_3$

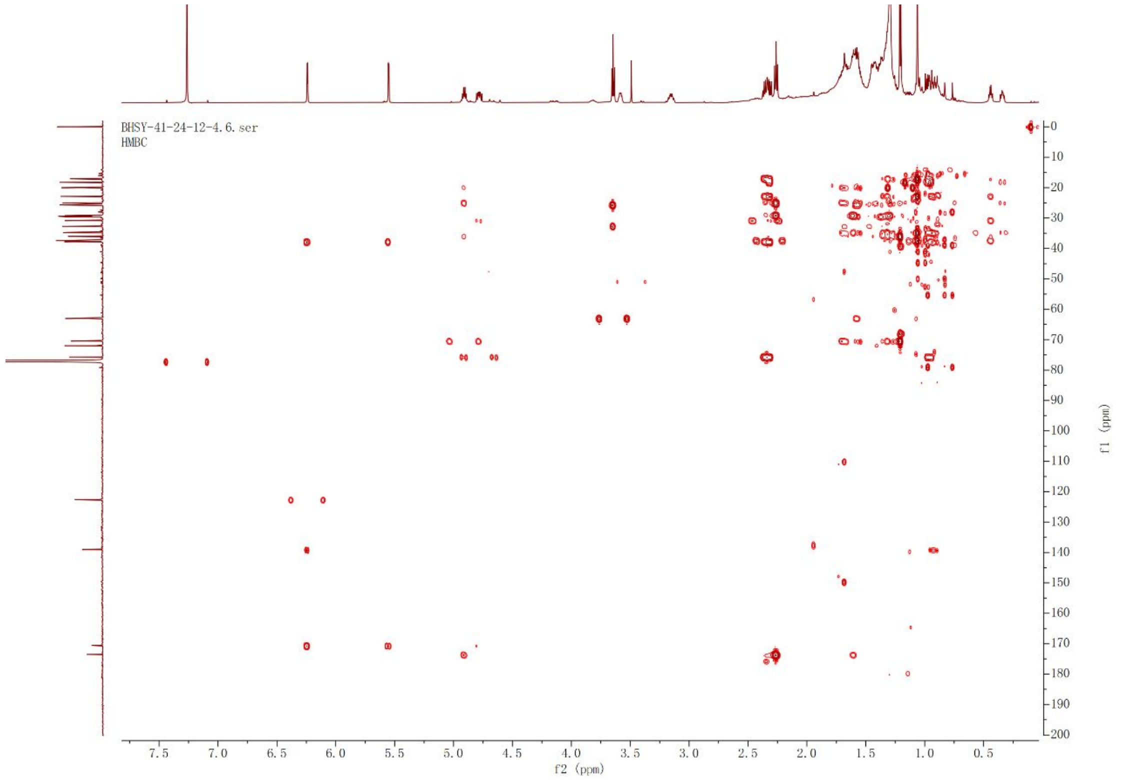


Figure S67. HMBC spectrum (600 MHz) of carabrolate G (**7**) in $CDCl_3$

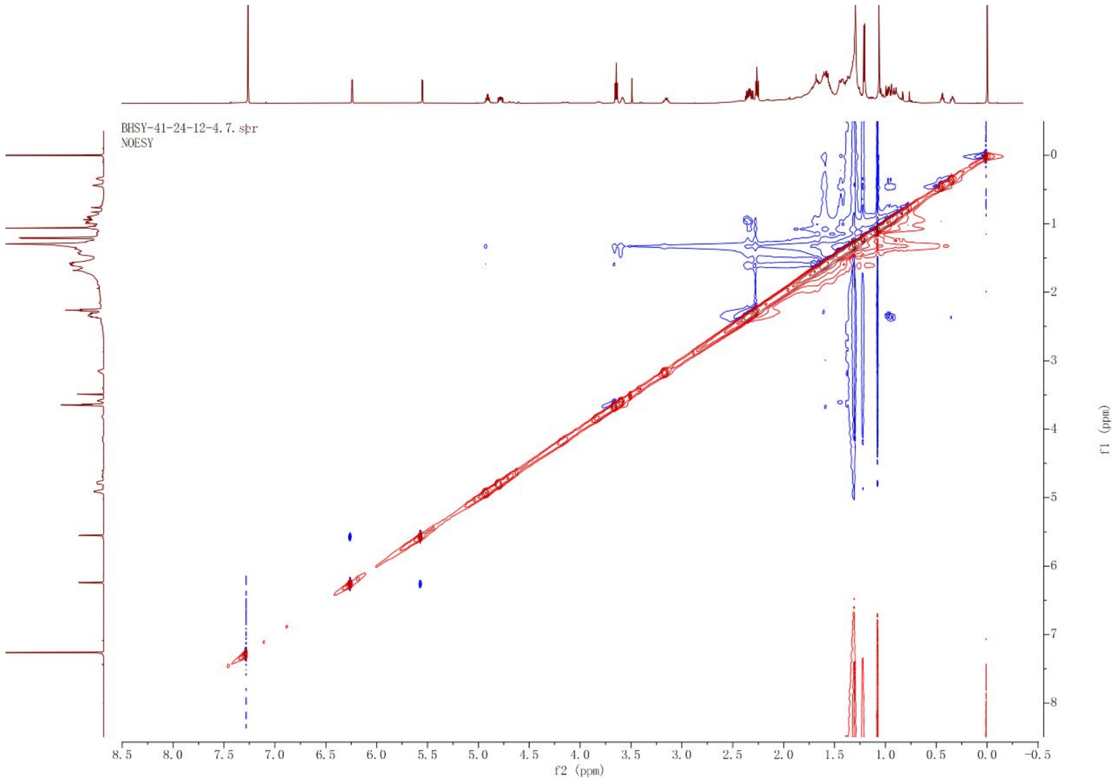


Figure S68. NOESY spectrum (600 MHz) of carabrolate G (**7**) in $CDCl_3$

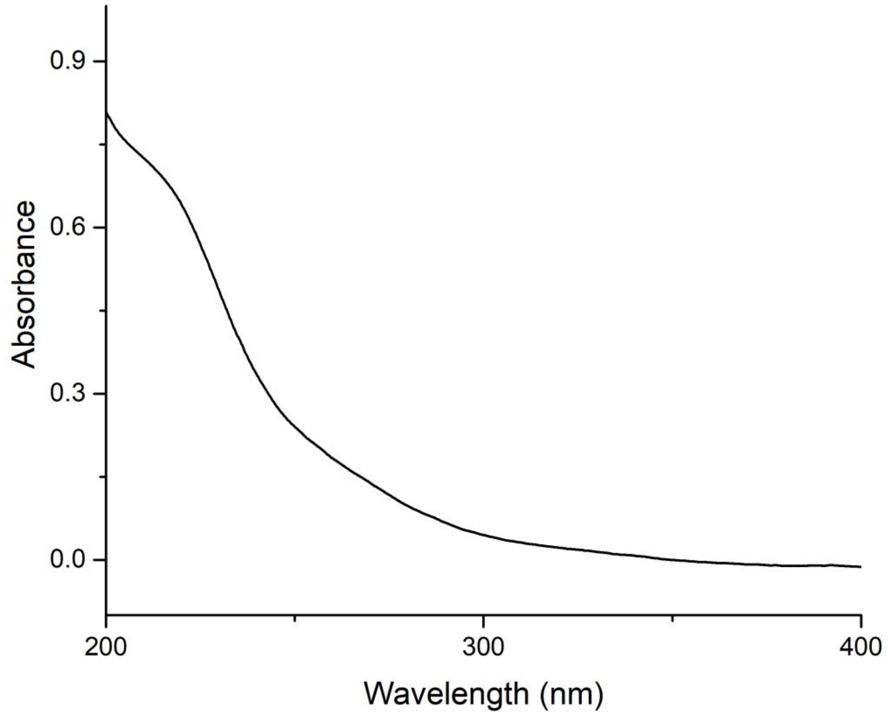


Figure S69. UV spectrum of carabrolate G (**7**) in MeOH

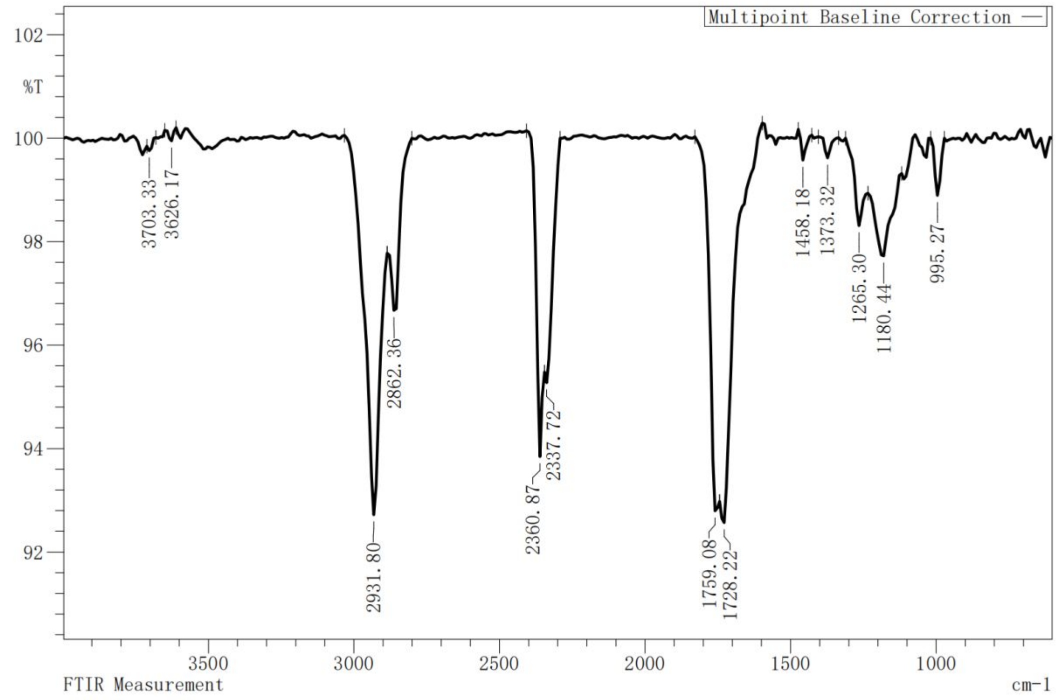


Figure S70. IR spectrum (film on KBr plate) of carabrolate G (**7**)

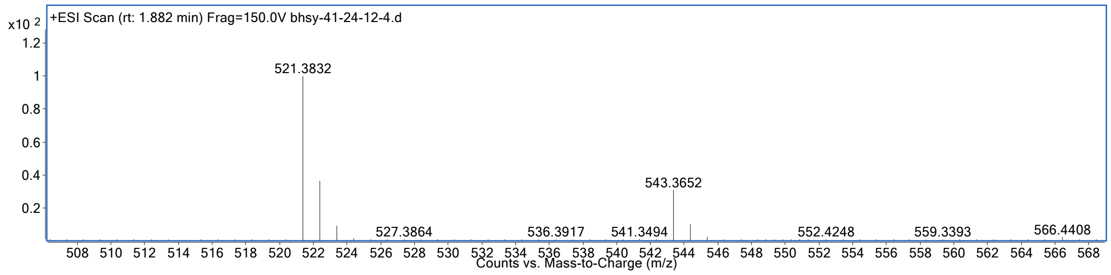


| Formula (M) | Score (MFG) | Mass | Mass (MFG) | m/z (Calc) | Diff (ppm) | Ion Formula | m/z |
|---|---|---|---|---|---|---|---|
| $C_{31}H_{52}O_6$ | 99.59 | 520.3759 | 520.3764 | 521.3837 | 0.9 | $C_{31}H_{53}O_6$ | 521.383 |
| $C_{31}H_{52}O_6$ | 99.68 | 520.376 | 520.3764 | 543.3656 | 0.79 | $C_{31}H_{52}NaO_6$ | 543.3652 |

Figure S71. HRESIMS spectrum of carabrolate G (**7**)

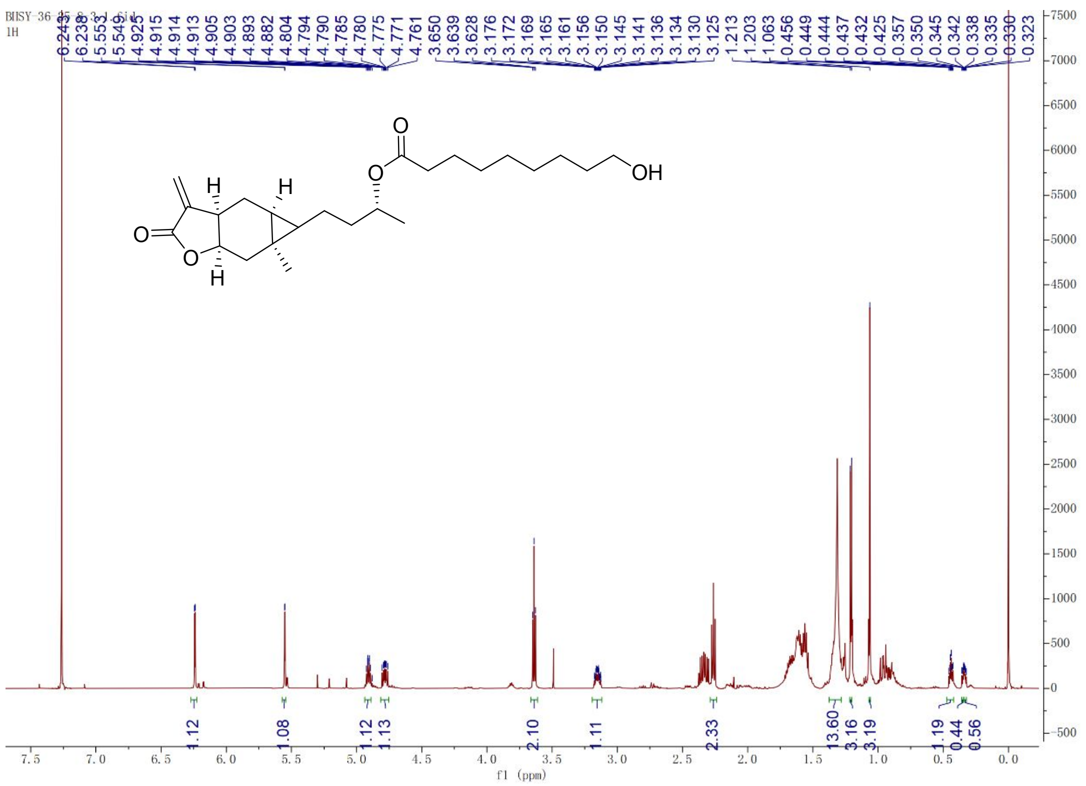


Figure S72. $^{1}H$ NMR spectrum (600 MHz) of carabrolate H (**8**) in $CDCl_3$

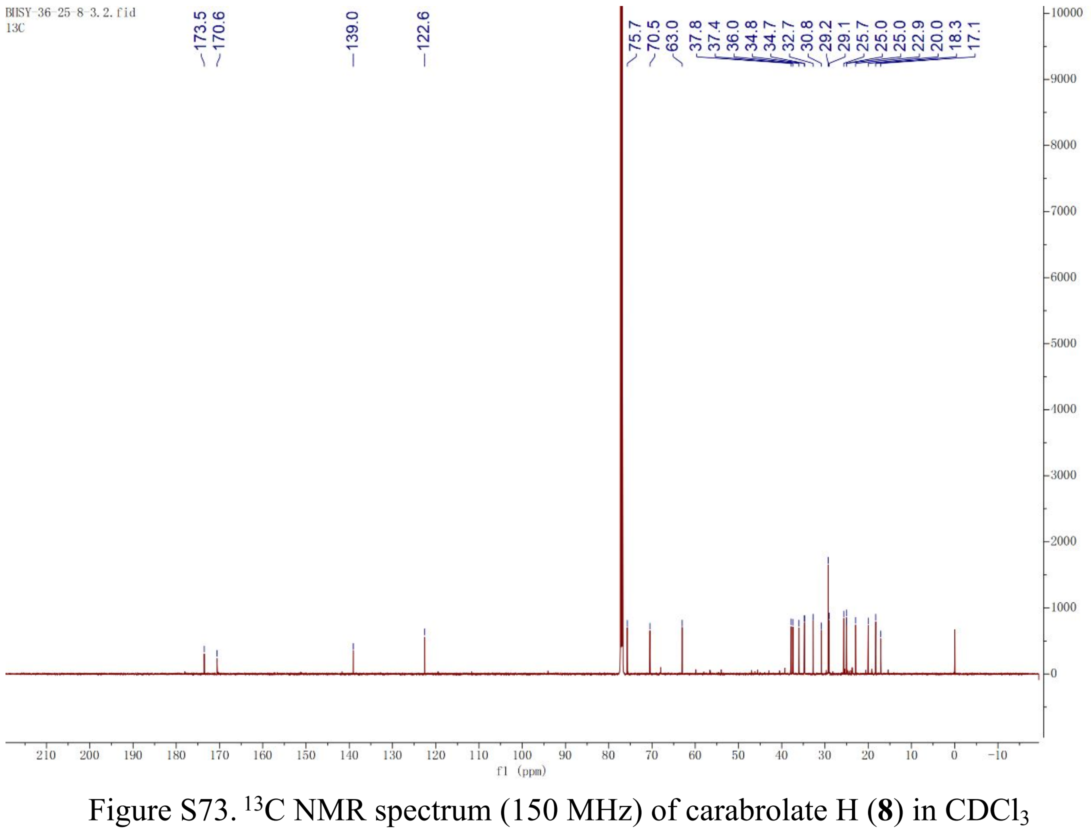


Figure S73. $^{13}C$ NMR spectrum (150 MHz) of carabrolate H (**8**) in $CDCl_3$

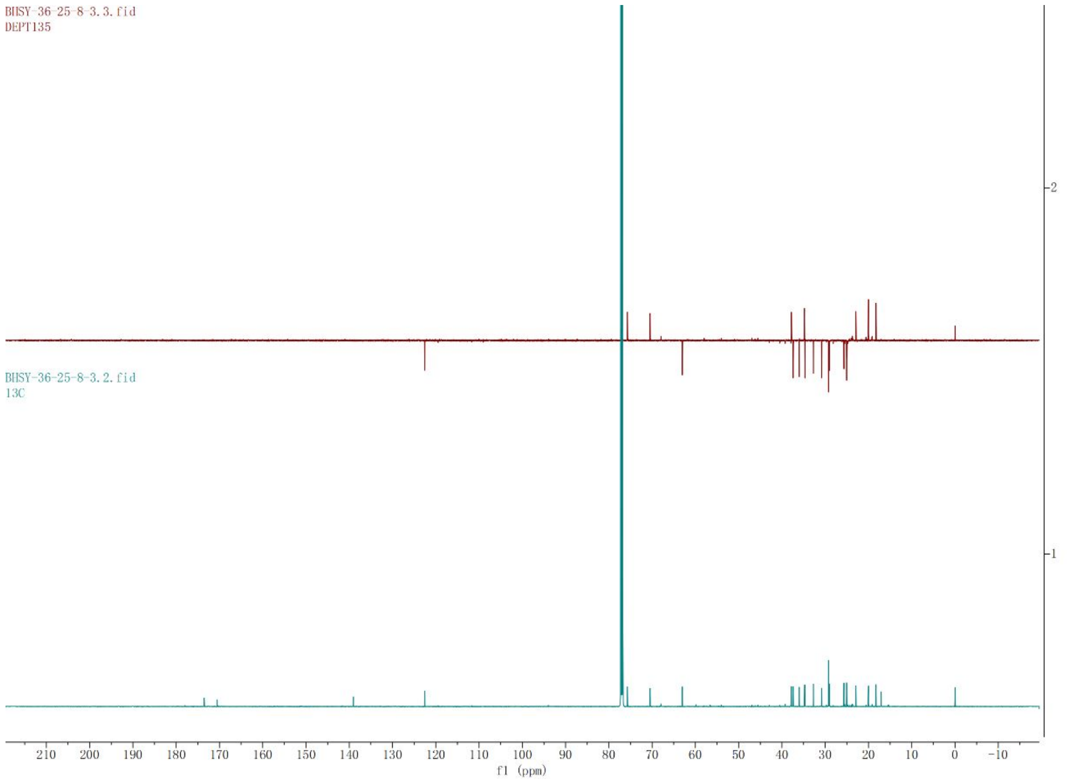


Figure S74. DEPT 135 spectrum (150 MHz) of carabrolate H (**8**) in $CDCl_3$

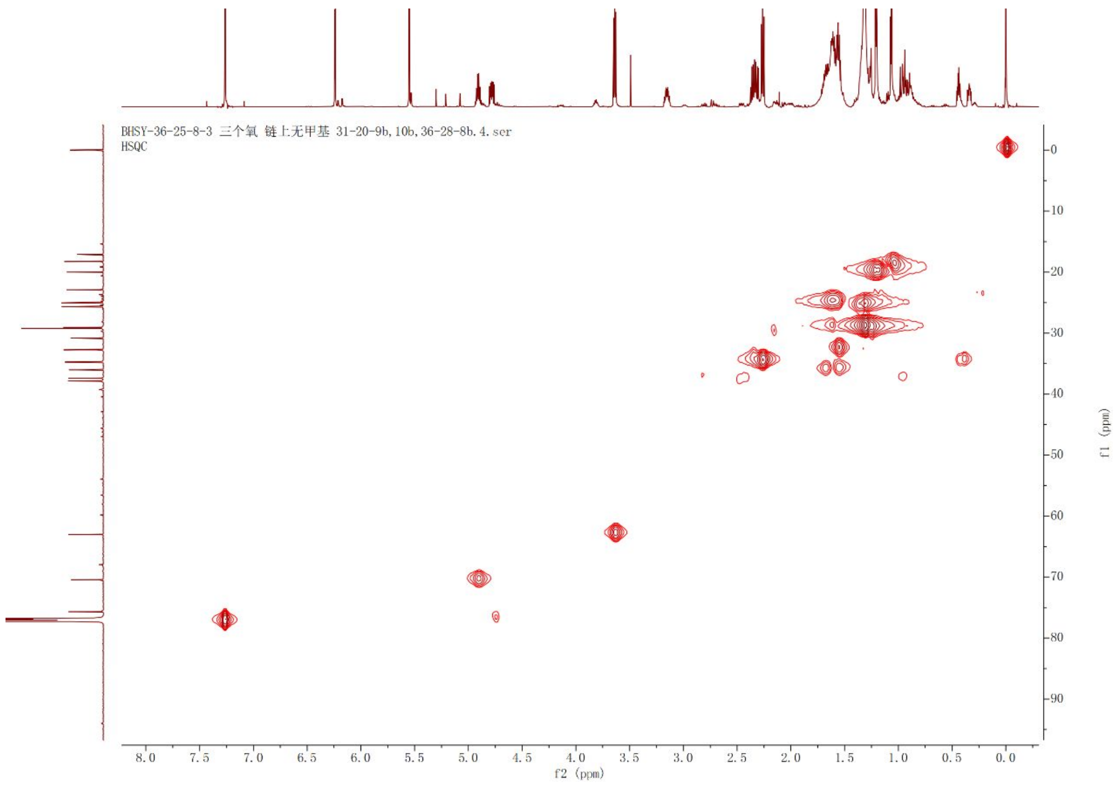


Figure S75. HSQC spectrum (600 MHz) of carabrolate H (**8**) in $CDCl_3$

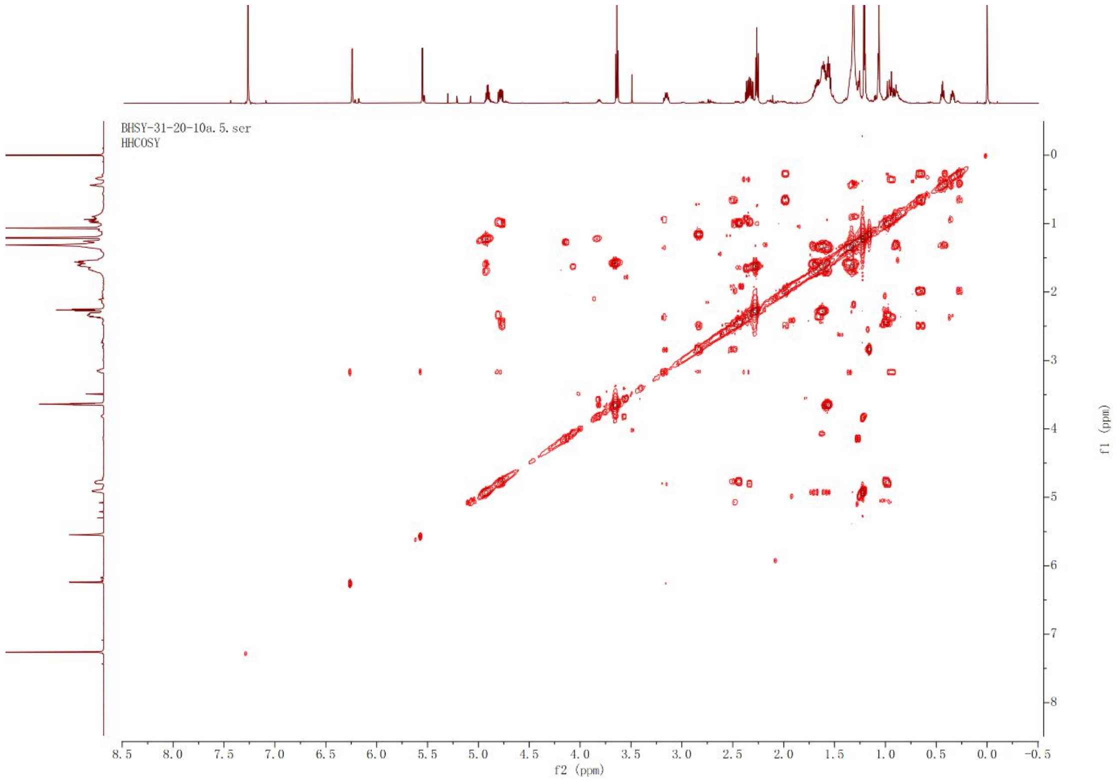


Figure S76. $^{1}H$-$^{1}H$ COSY spectrum (600 MHz) of carabrolate H (**8**) in $CDCl_3$

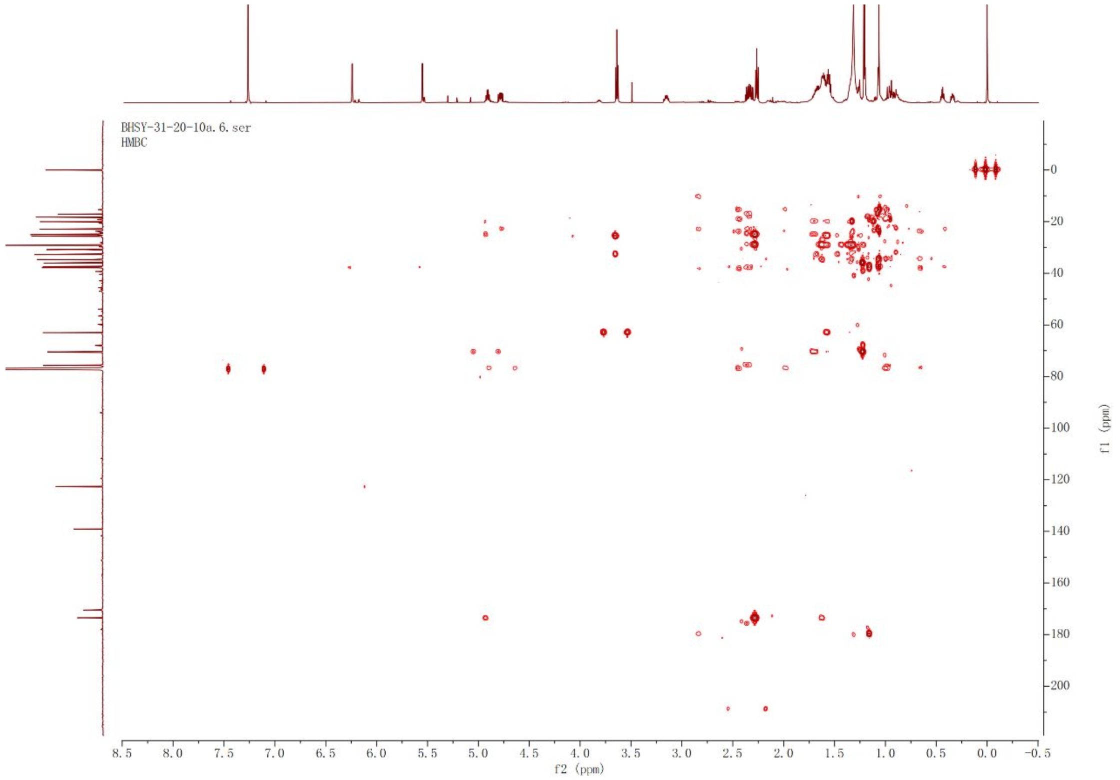


Figure S77. HMBC spectrum (600 MHz) of carabrolate H (**8**) in $CDCl_3$

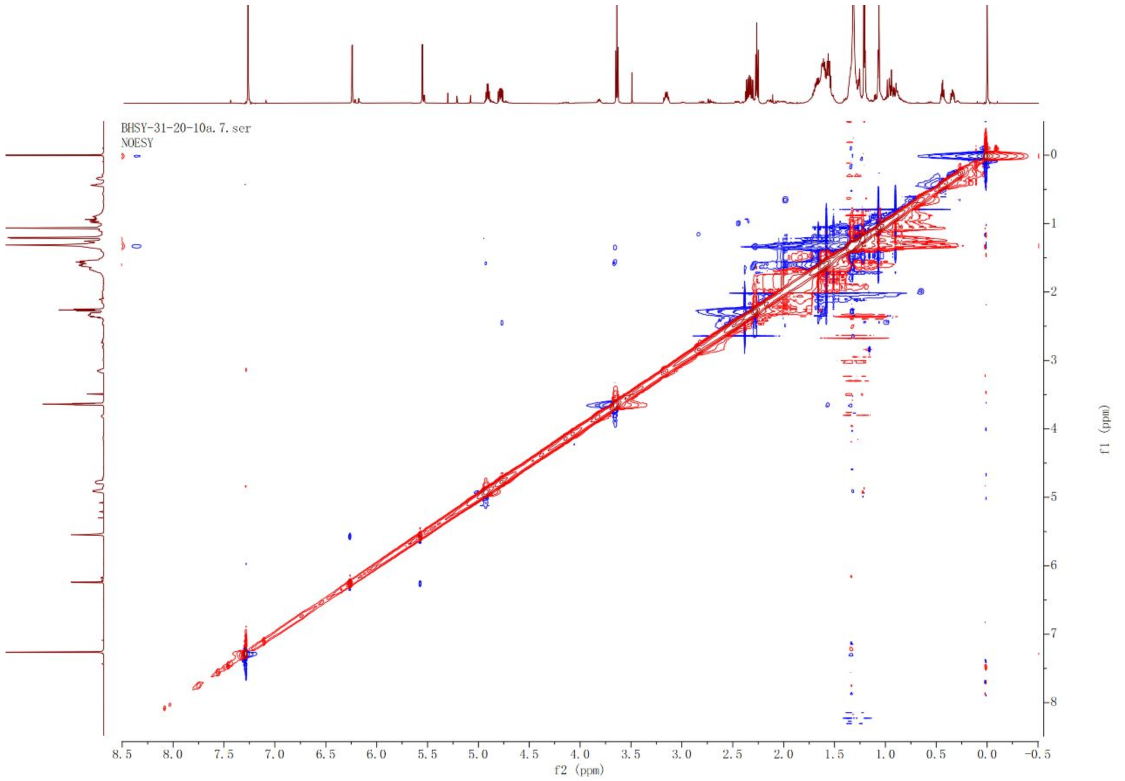


Figure S78. NOESY spectrum (600 MHz) of carabrolate H (**8**) in $CDCl_3$

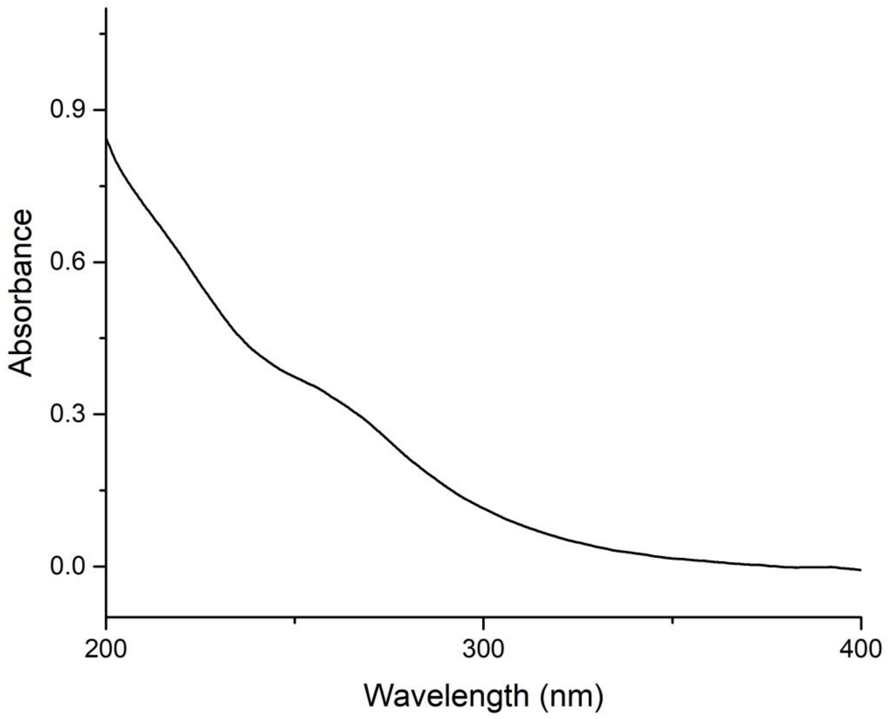


Figure S79. UV spectrum of carabrolate H (**8**) in MeOH

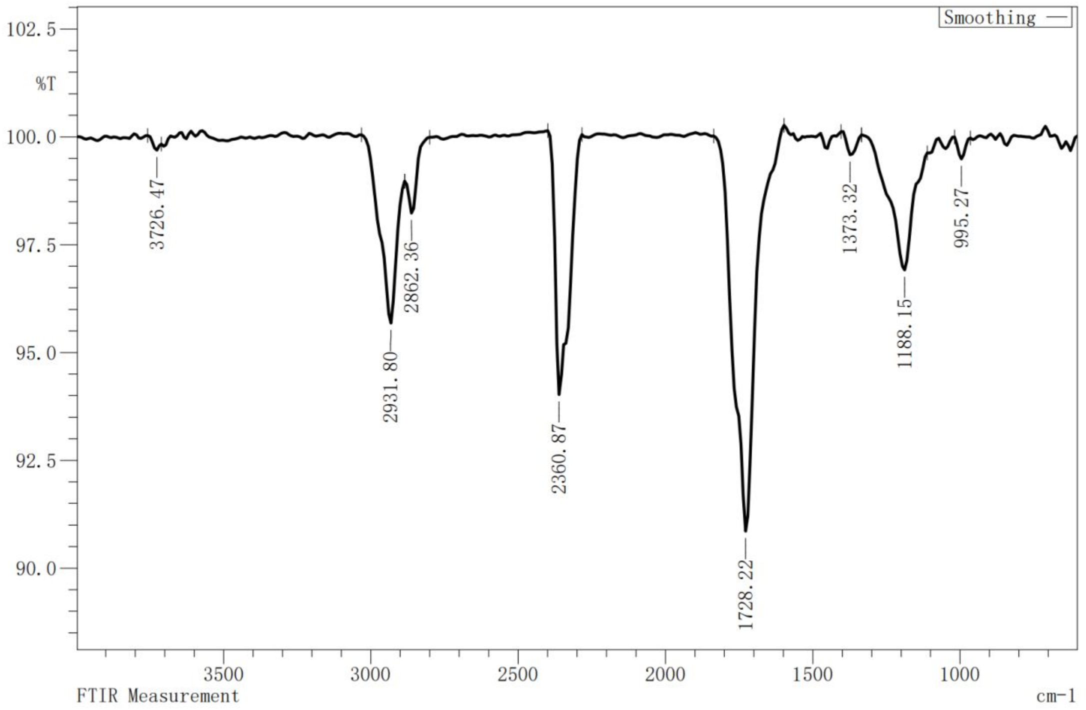


Figure S80. IR spectrum (film on KBr plate) of carabrolate H (**8**)

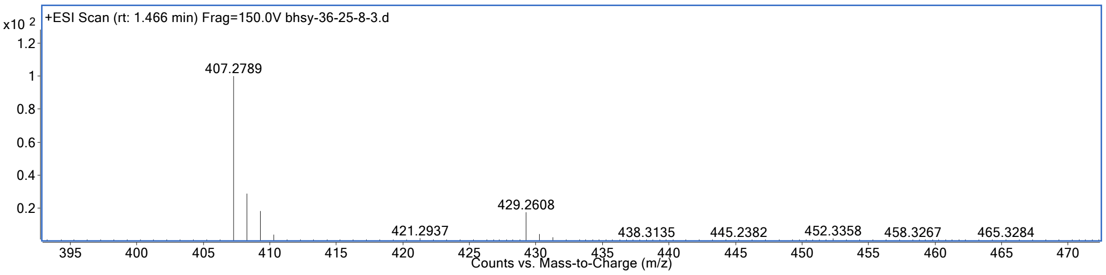


| Formula (M) | Score (MFG) | Mass | Mass (MFG) | m/z (Calc) | Diff (ppm) | Ion Formula | m/z |
|---|---|---|---|---|---|---|---|
| $C_{24}H_{38}O_5$ | 99.78 | 406.2716 | 406.2719 | 407.2792 | 0.74 | $C_{24}H_{39}O_5$ | 407.2789 |
| $C_{24}H_{38}O_5$ | 99.7 | 406.2716 | 406.2719 | 429.2611 | 0.85 | $C_{24}H_{38}NaO_5$ | 429.2608 |

Figure S81. HRESIMS spectrum of carabrolate H (**8**)

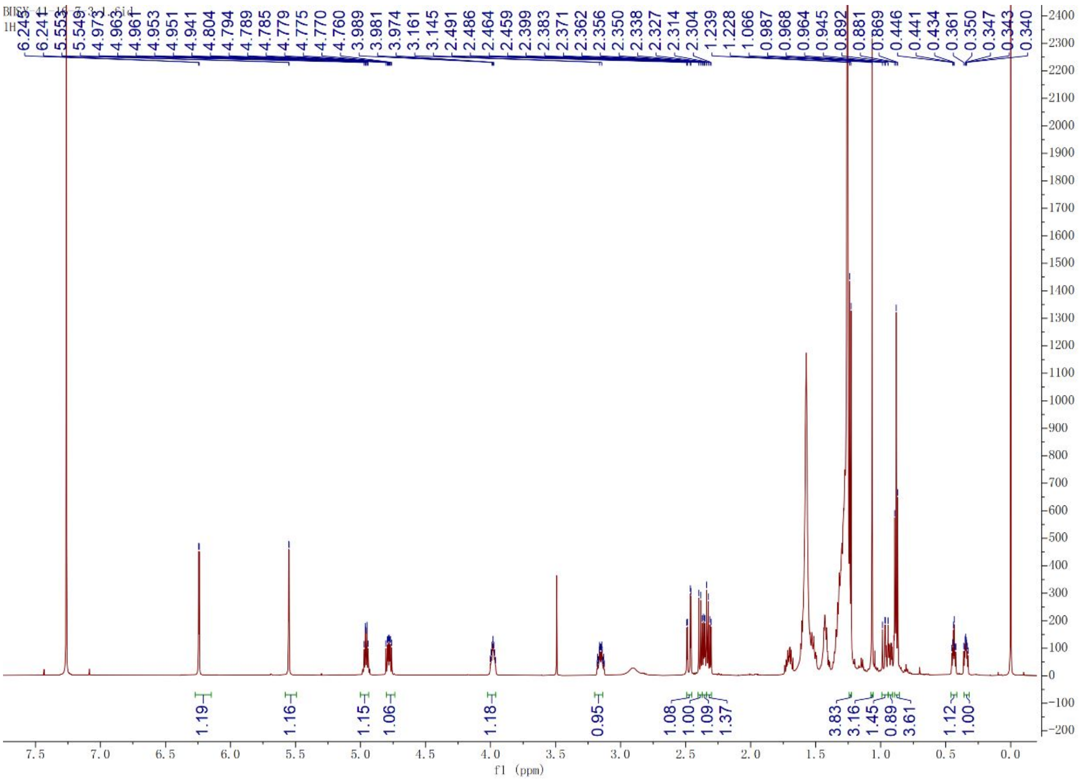


Figure S82. $^{1}$H NMR spectrum (600 MHz) of carabrolate I (**9**) in $CDCl_3$

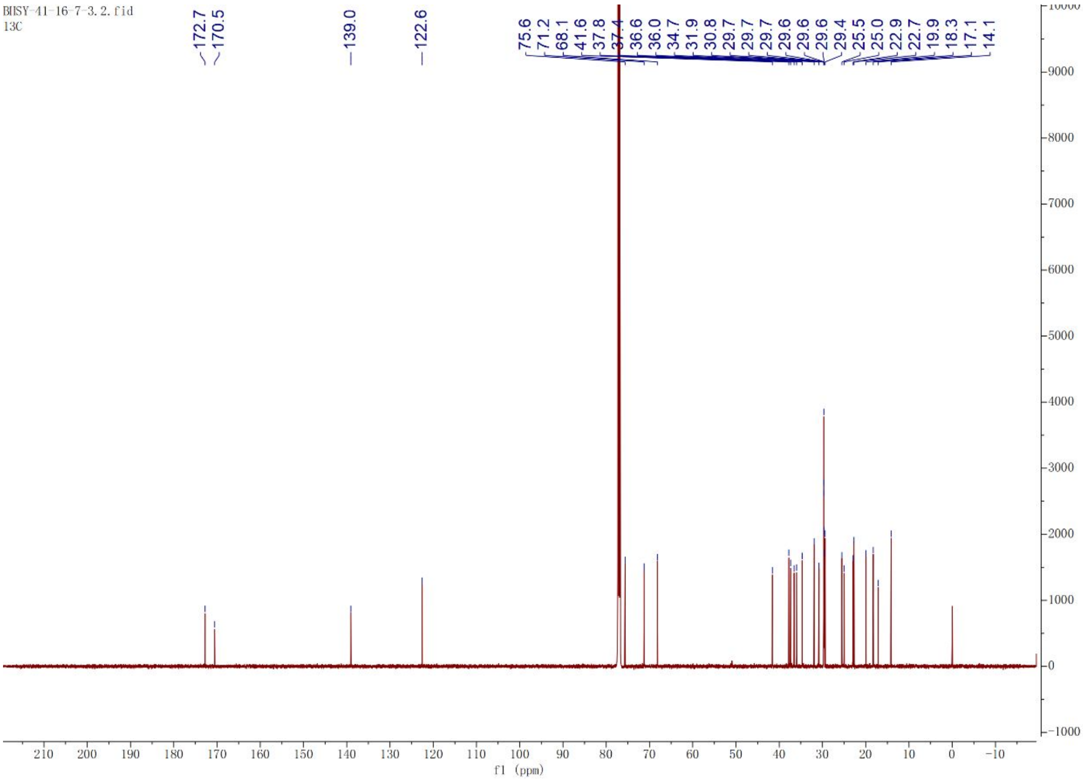


Figure S83. $^{13}$C NMR spectrum (150 MHz) of carabrolate I (**9**) in $CDCl_3$

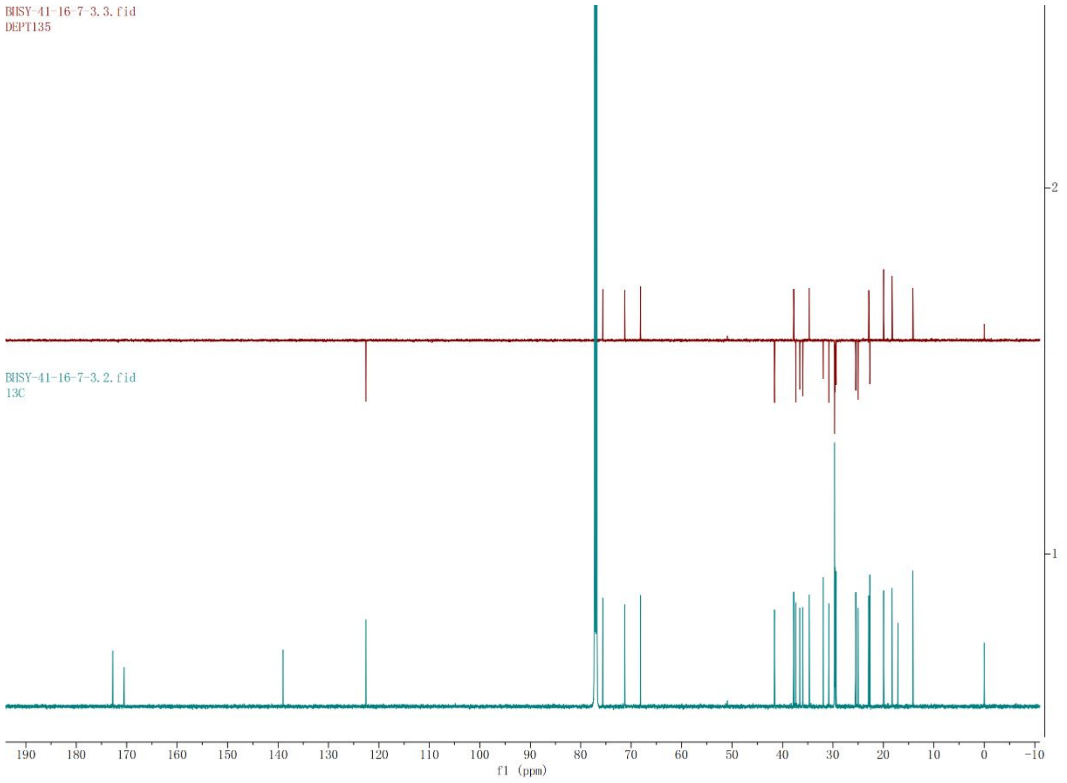


Figure S84. DEPT 135 spectrum (150 MHz) of carabrolate I (**9**) in $CDCl_3$

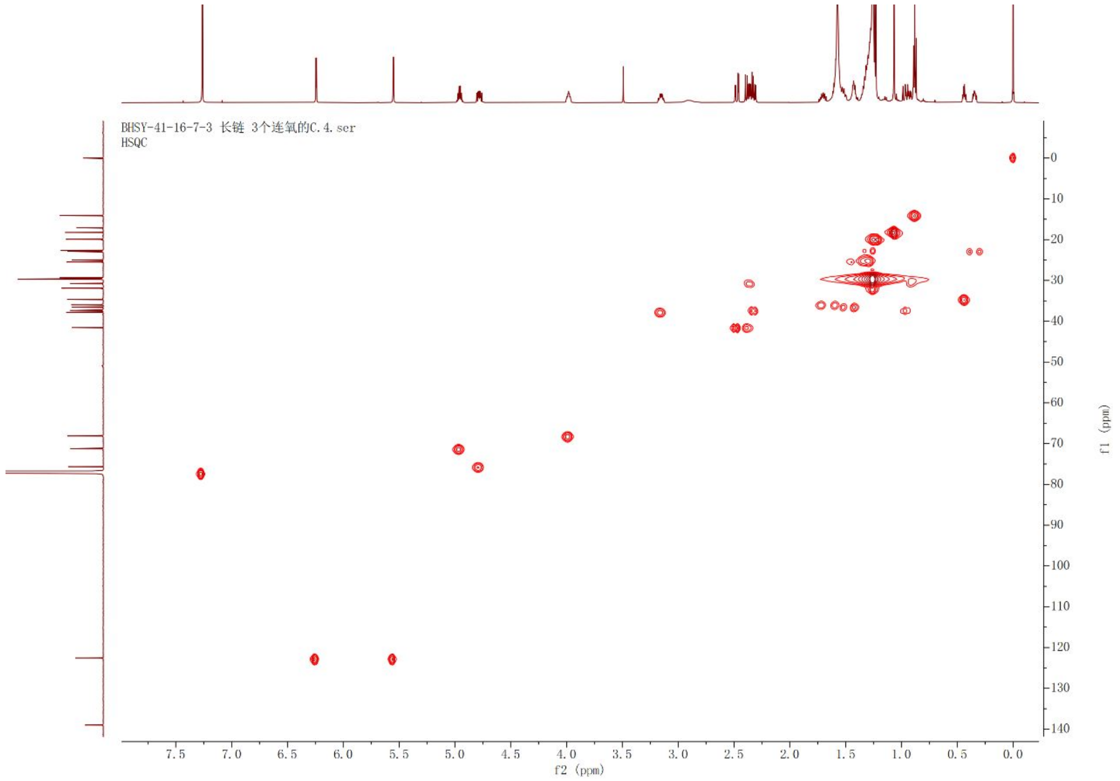


Figure S85. HSQC spectrum (600 MHz) of carabrolate I (**9**) in $CDCl_3$

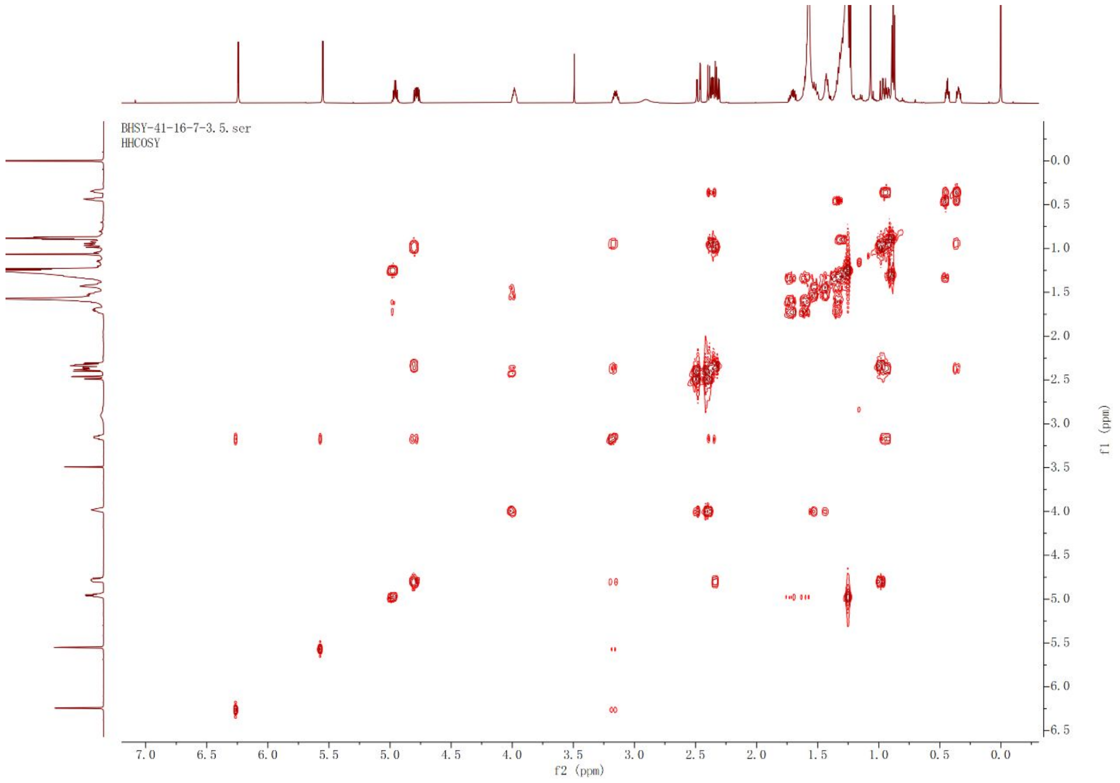


Figure S86. $^1$H-$^1$H COSY spectrum (600 MHz) of carabrolate I (**9**) in $CDCl_3$

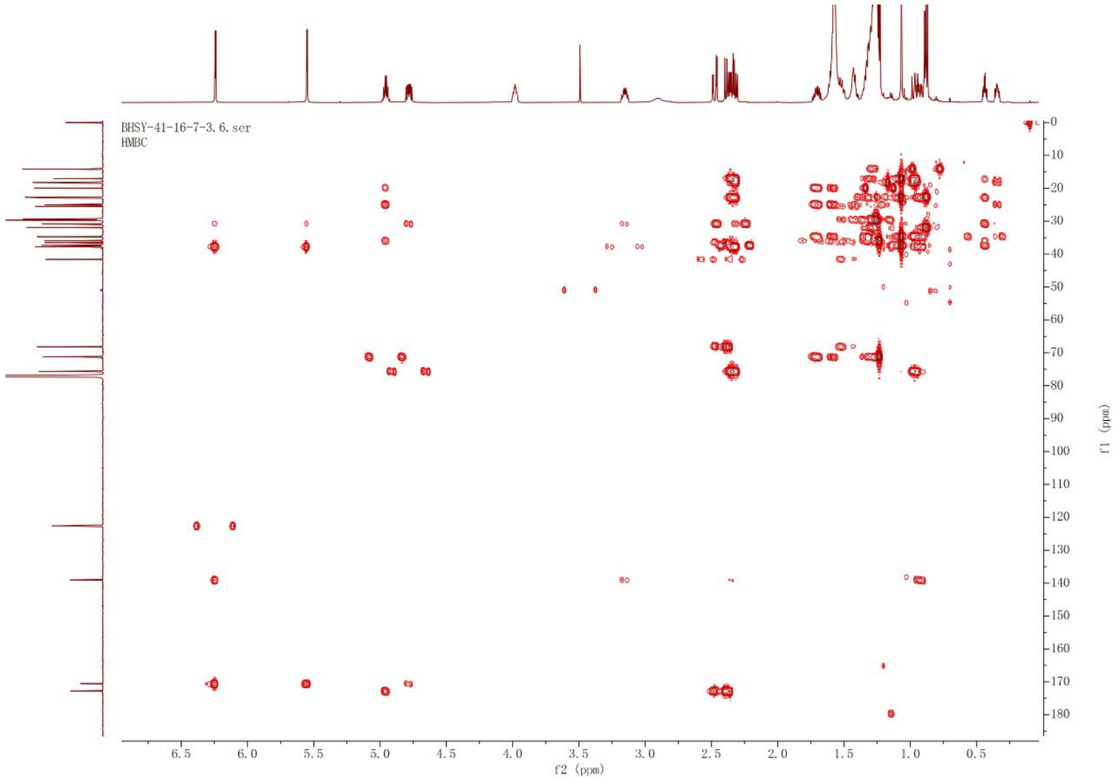


Figure S87. HMBC spectrum (600 MHz) ofcarabrolate I (**9**) in $CDCl_3$

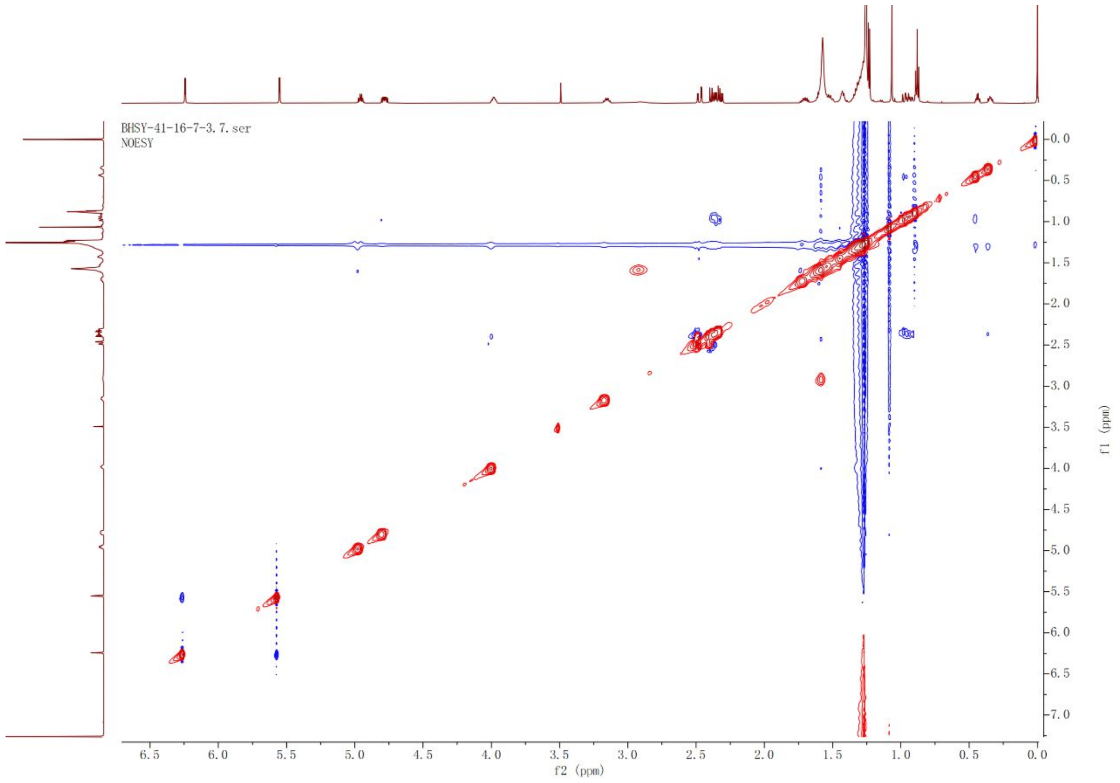


Figure S88. NOESY spectrum (600 MHz) of carabrolate I (**9**) in $CDCl_3$

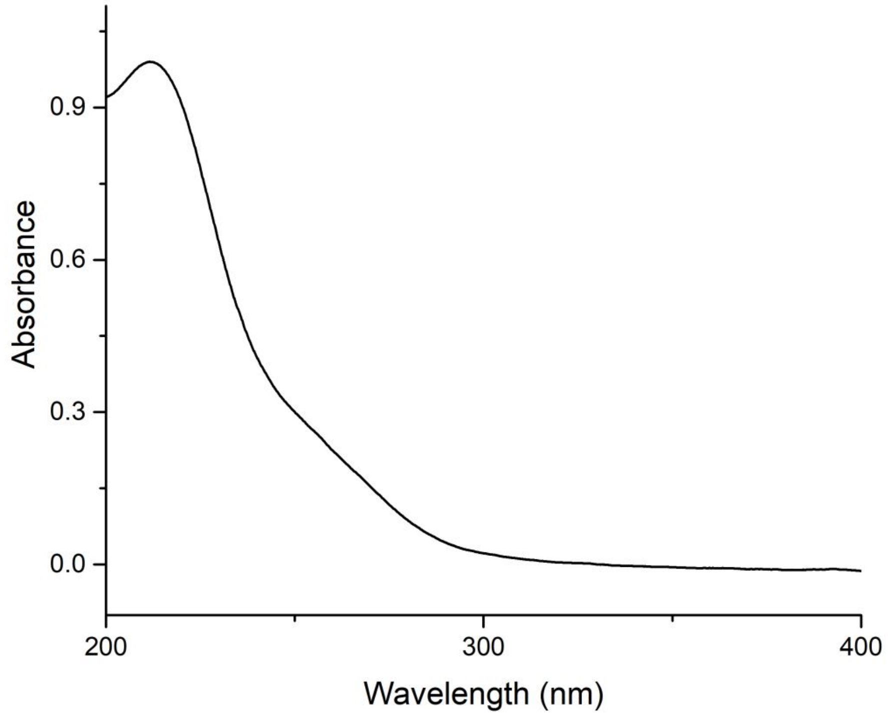


Figure S89. UV spectrum of carabrolate I (**9**) in MeOH

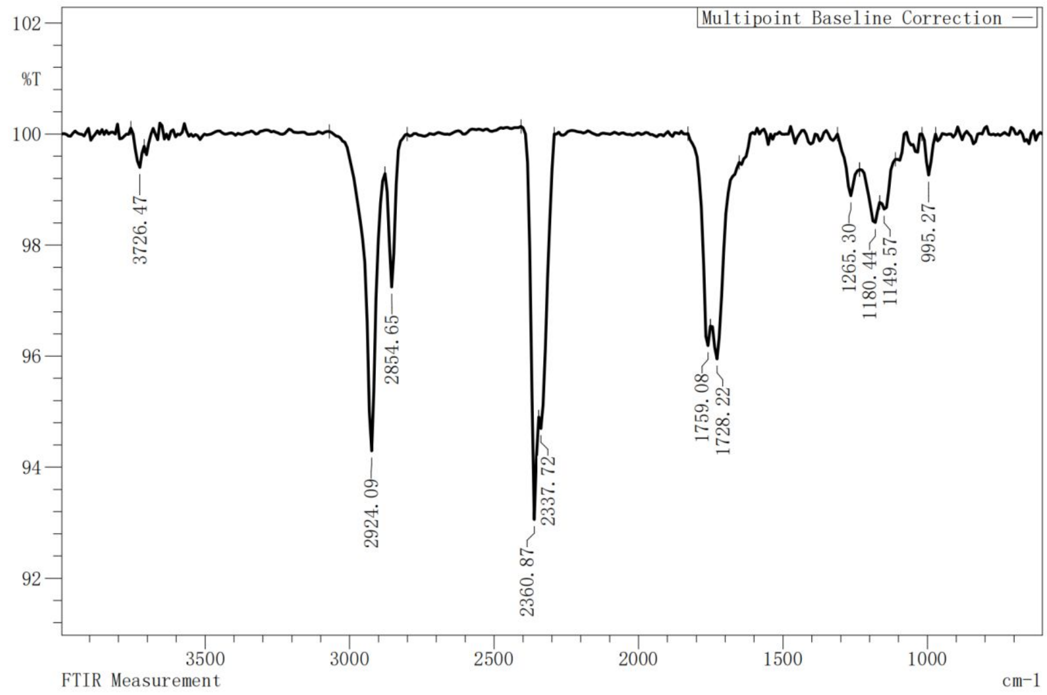


Figure S90. IR spectrum (film on KBr plate) of carabrolate I (**9**)

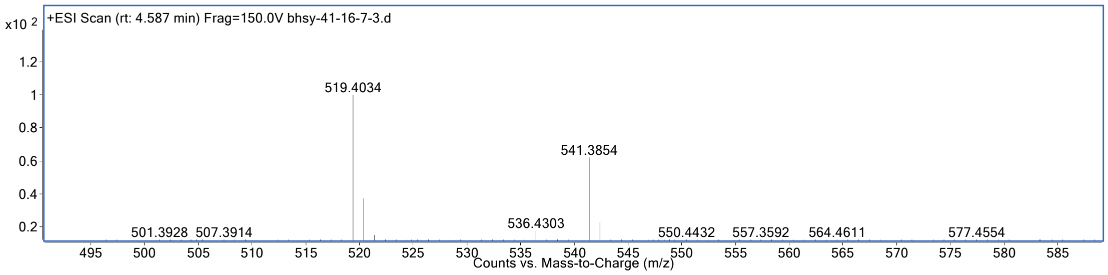


| Formula (M) | Score (MFG) | Mass | Mass (MFG) | m/z (Calc) | Diff (ppm) | Ion Formula | m/z |
|---|---|---|---|---|---|---|---|
| $C_{32}H_{54}O_5$ | 98.14 | 518.3961 | 518.3971 | 519.4044 | 1.93 | $C_{32}H_{55}O_5$ | 519.4034 |
| $C_{32}H_{54}O_5$ | 98.34 | 518.3962 | 518.3971 | 541.3863 | 1.82 | $C_{32}H_{54}NaO_5$ | 541.3854 |

Figure S91. HRESIMS spectrum of carabrolate I (**9**)

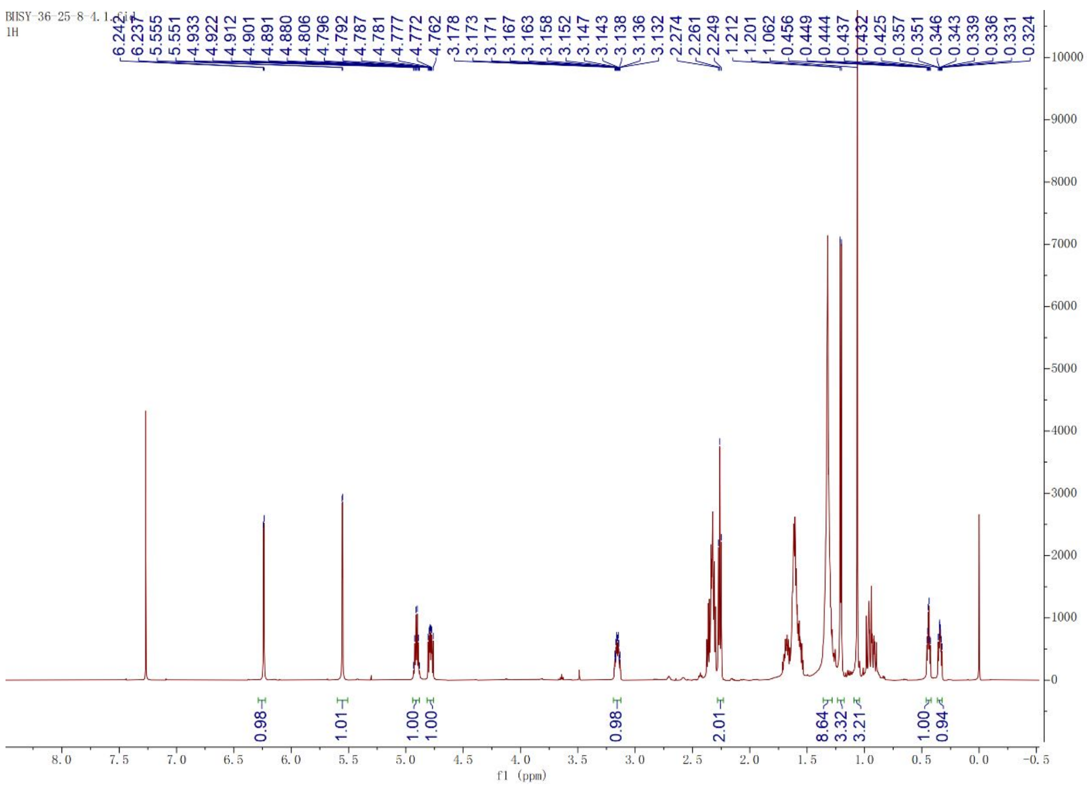


Figure S92. $^{1}$H NMR spectrum (600 MHz) of carabrolate J (**10**) in $CDCl_3$

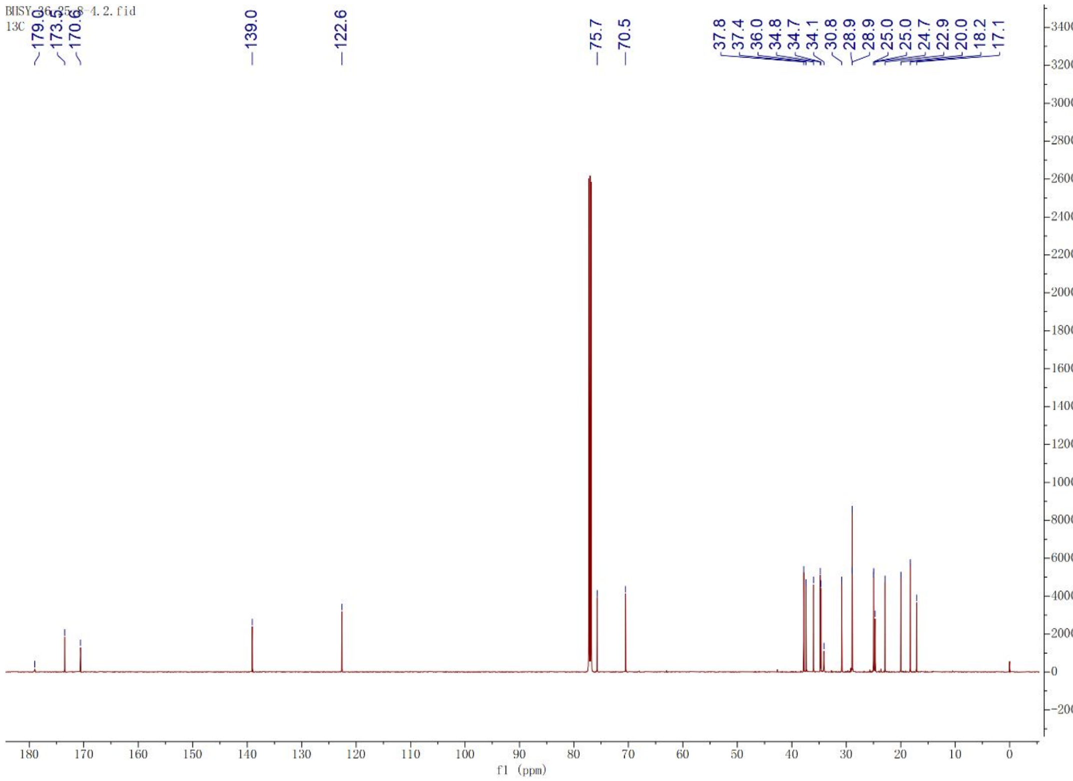


Figure S93. $^{13}$C NMR spectrum (150 MHz) of carabrolate J (**10**) in $CDCl_3$

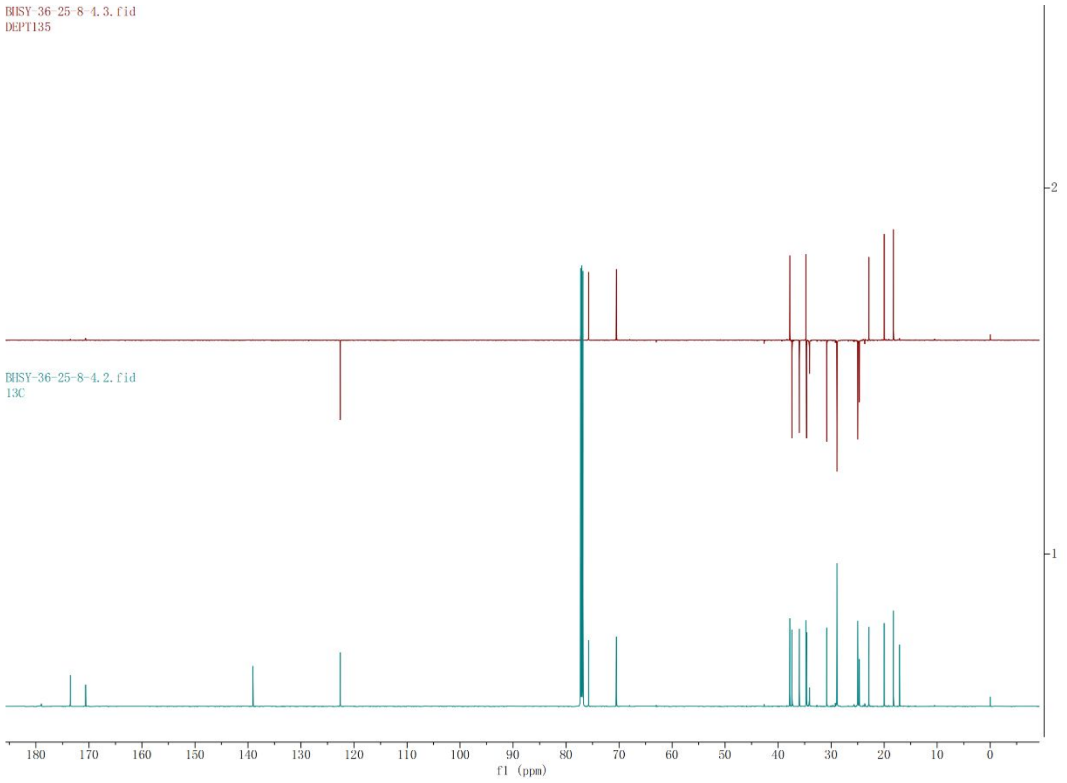


Figure S94. DEPT 135 spectrum (150 MHz) of carabrolate J (**10**) in $CDCl_3$

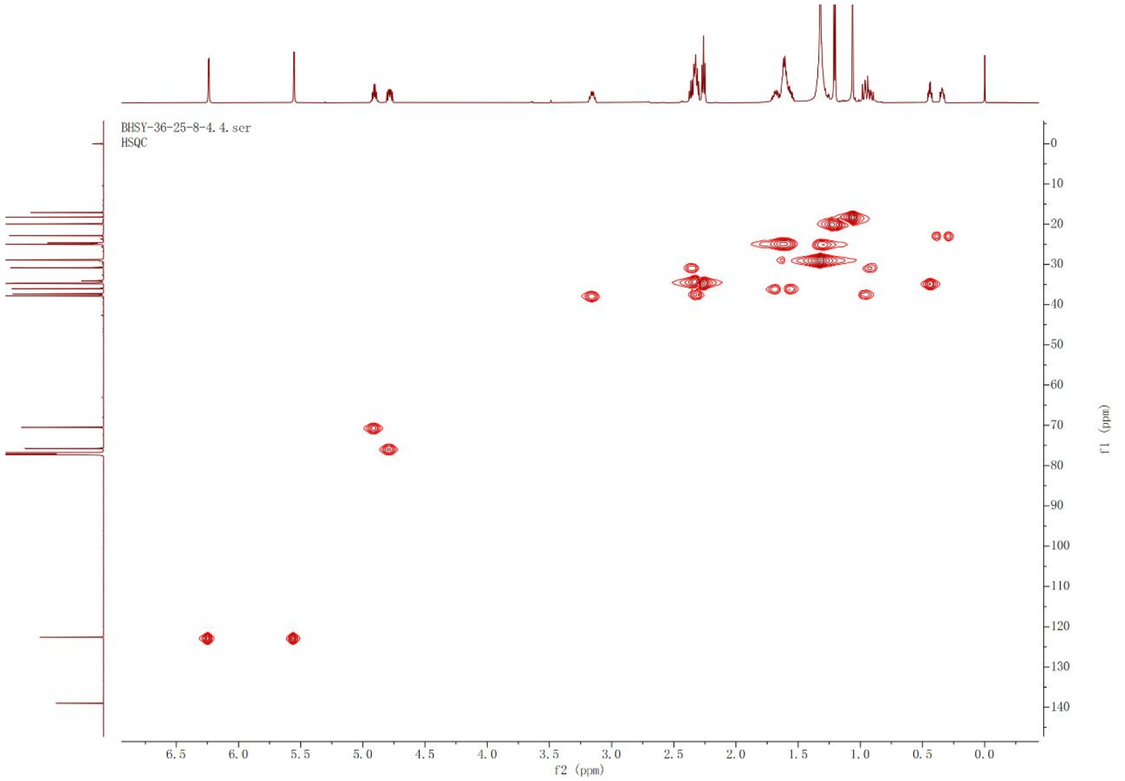


Figure S95. HSQC spectrum (600 MHz) of carabrolate J (**10**) in $CDCl_3$

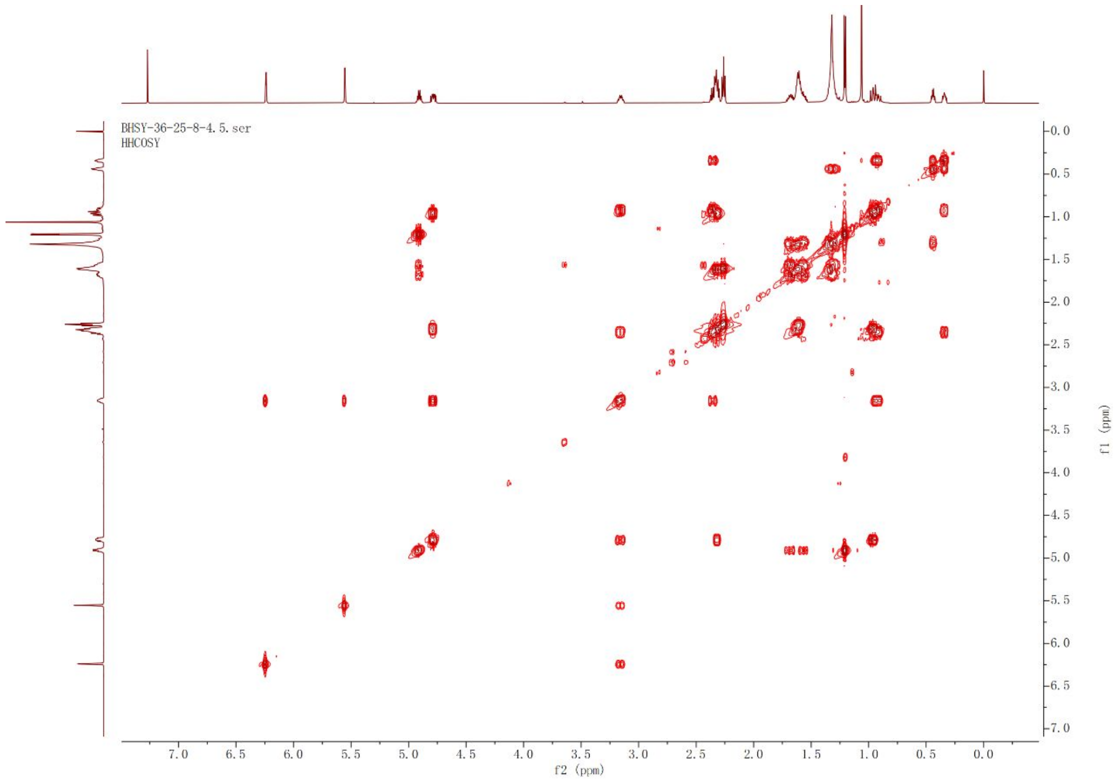


Figure S96. $^{1}H$-$^{1}H$ COSY spectrum (600 MHz) of carabrolate J (**10**) in $CDCl_3$

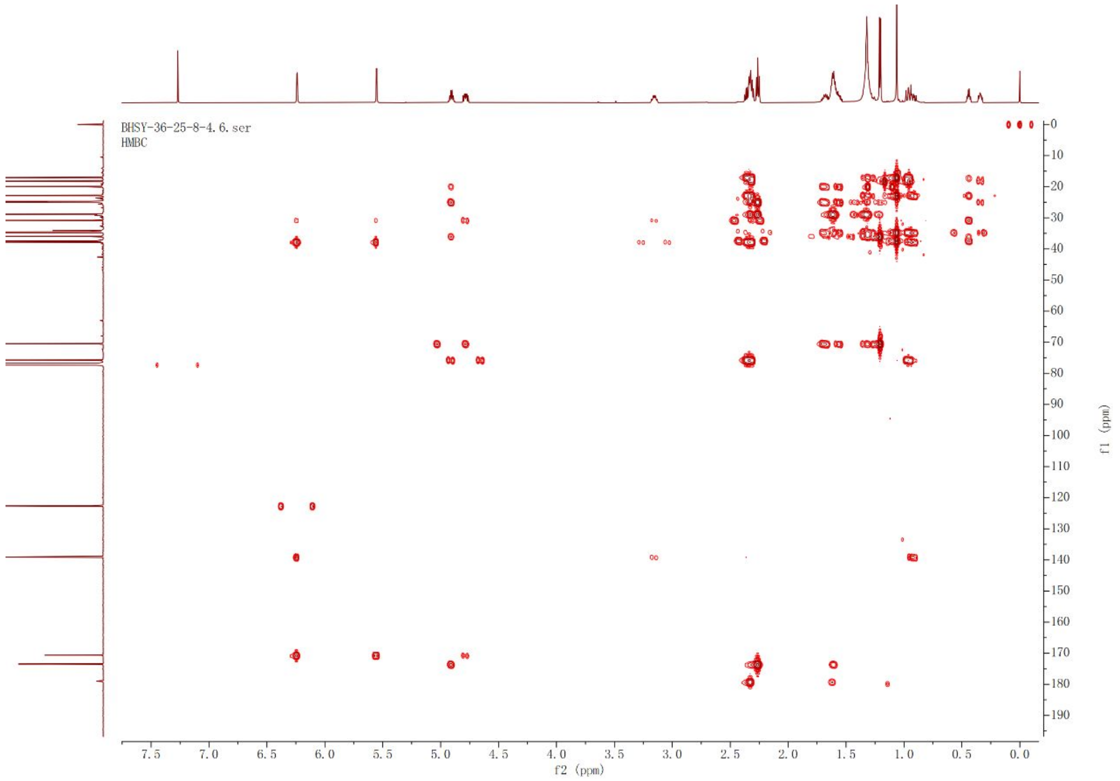


Figure S97. HMBC spectrum (600 MHz) of carabrolate J (**10**) in $CDCl_3$

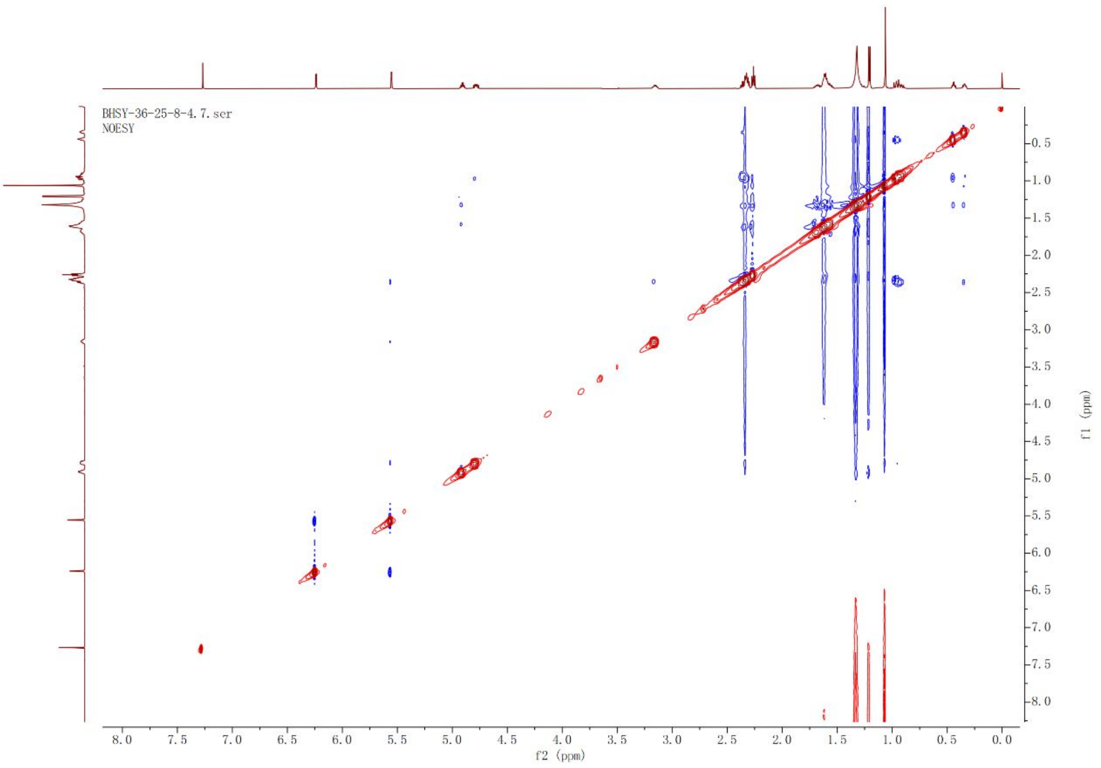


Figure S98. NOESY spectrum (600 MHz) of carabrolate J (**10**) in $CDCl_3$

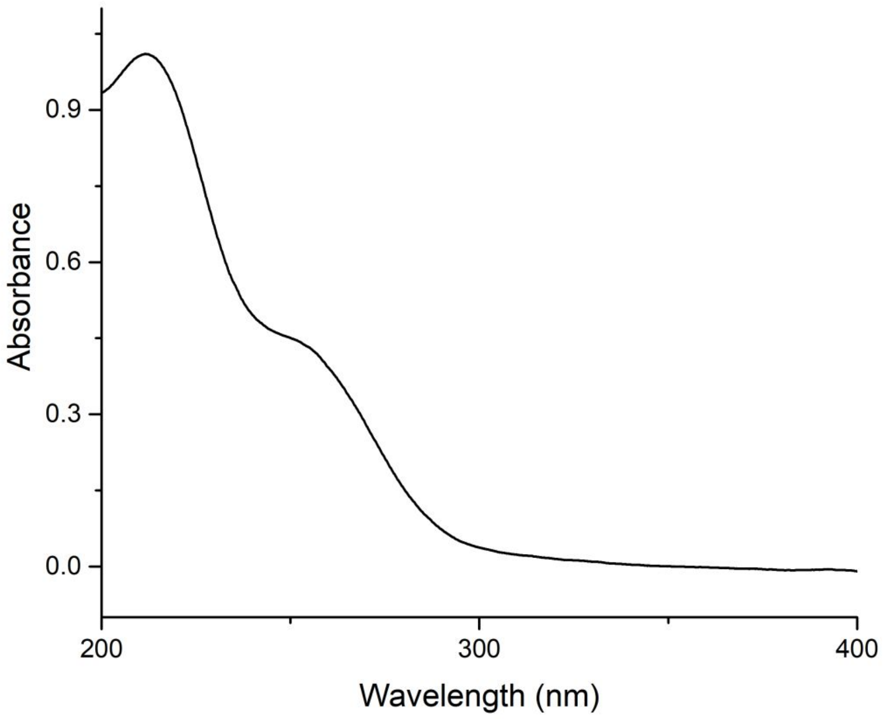


Figure S99. UV spectrum of carabrolate J (**10**) in MeOH

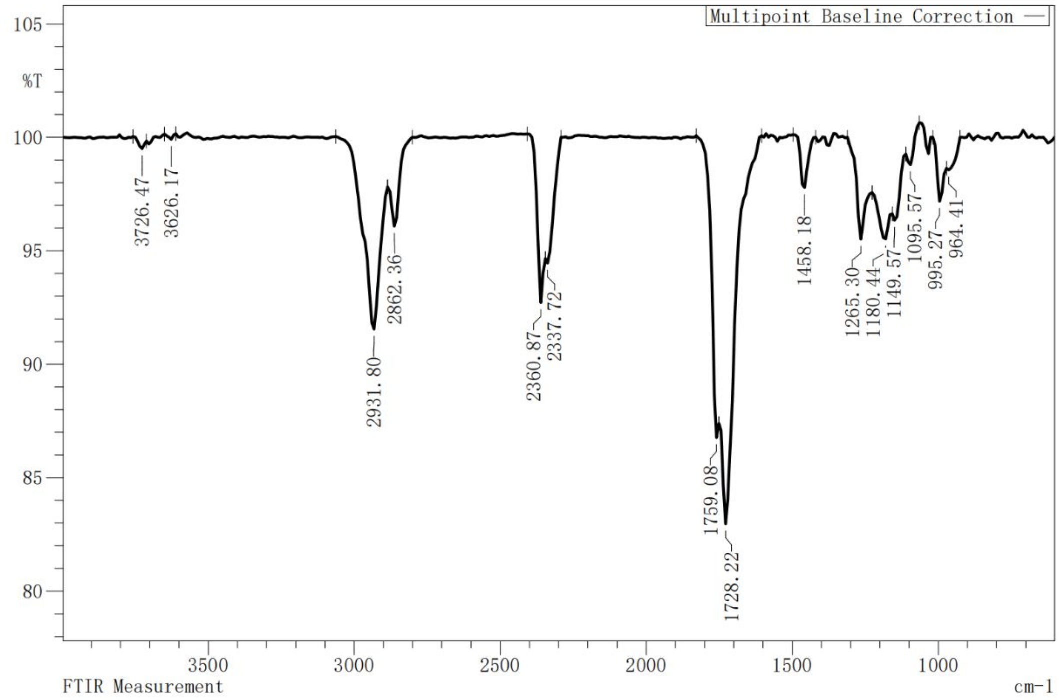


Figure S100. IR spectrum (film on KBr plate) of carabrolate J (**10**)

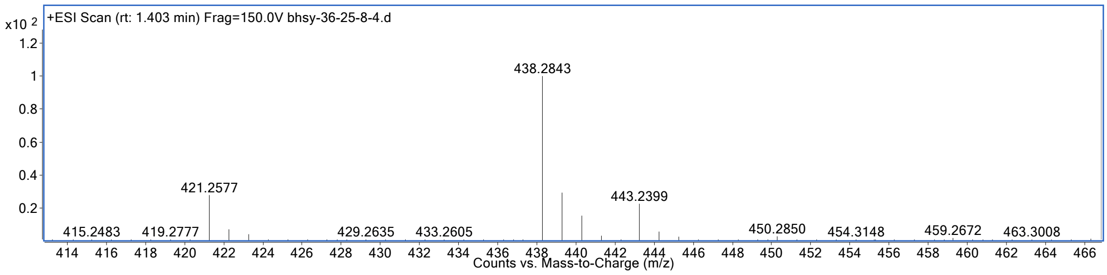


| Formula (M) | Score (MFG) | Mass | Mass (MFG) | m/z (Calc) | Diff (ppm) | Ion Formula | m/z |
|---|---|---|---|---|---|---|---|
| $C_{24}H_{36}O_6$ | 98.62 | 420.2504 | 420.2512 | 421.2585 | 1.82 | $C_{24}H_{37}O_6$ | 421.2577 |
| $C_{24}H_{36}O_6$ | 98.79 | 420.2505 | 420.2512 | 438.285 | 1.7 | $C_{24}H_{40}NO_6$ | 438.2843 |

Figure S101. HRESIMS spectrum of carabrolate J (**10**)

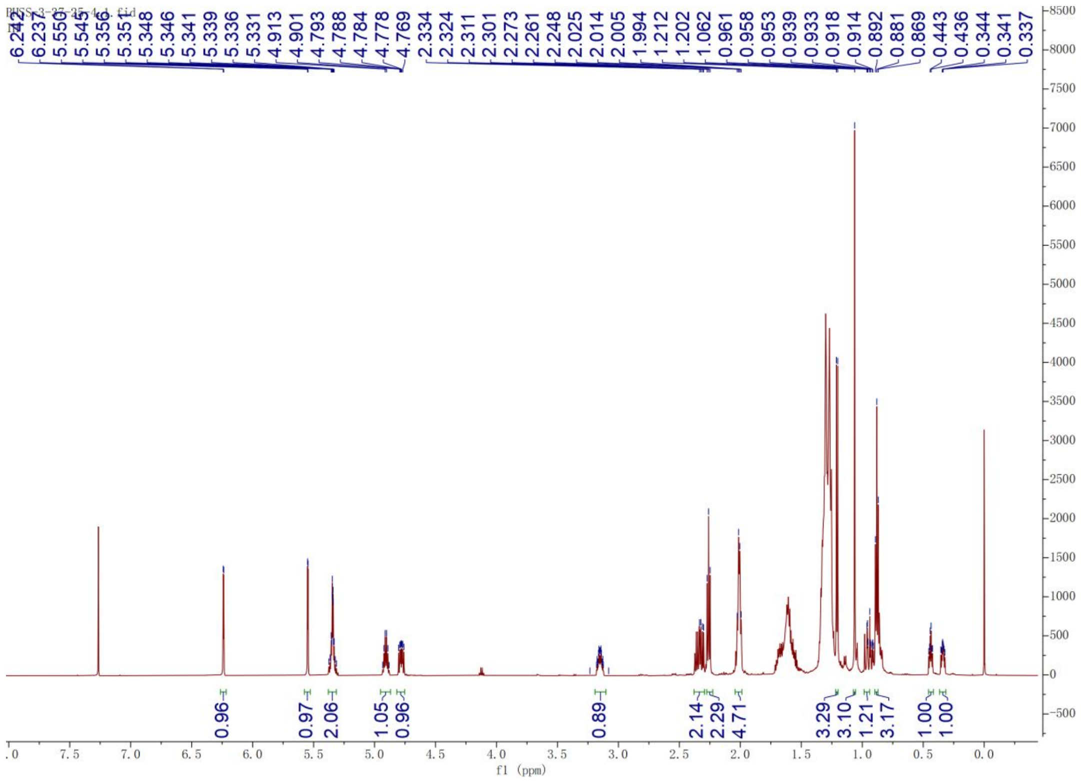


Figure S102. $^{1}$H NMR spectrum (600 MHz) of carabrolate K (**11**) in $CDCl_3$

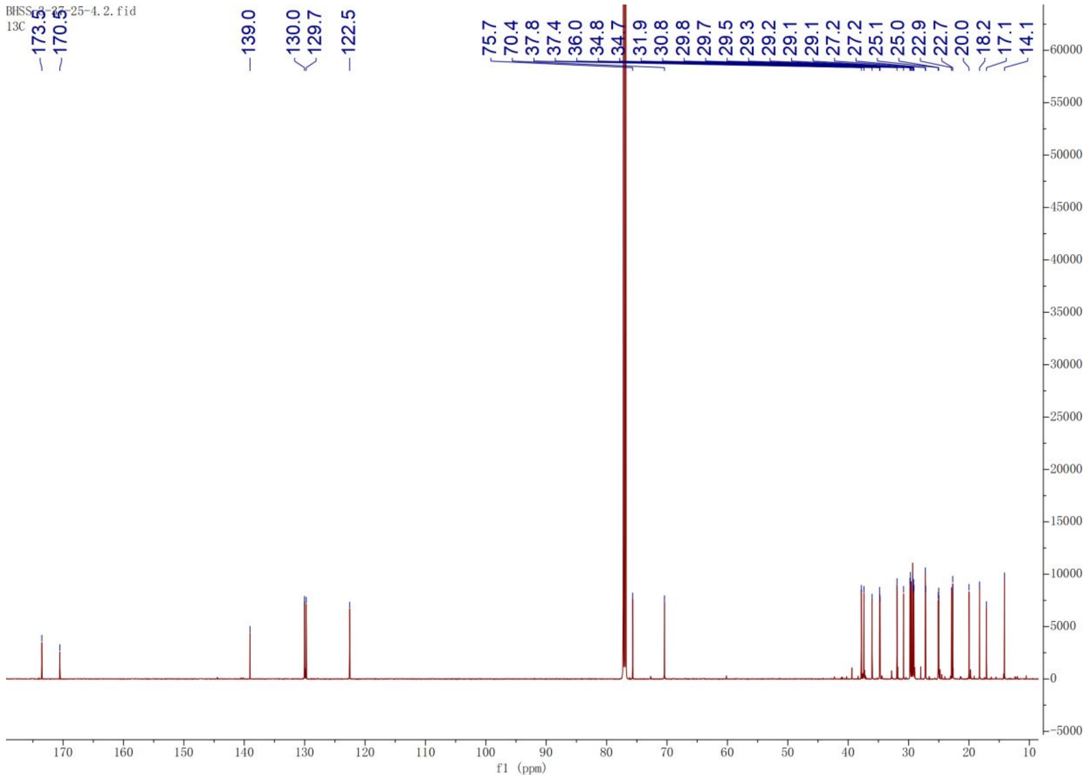


Figure S103. $^{13}$C NMR spectrum (150 MHz) of carabrolate K (**11**) in $CDCl_3$

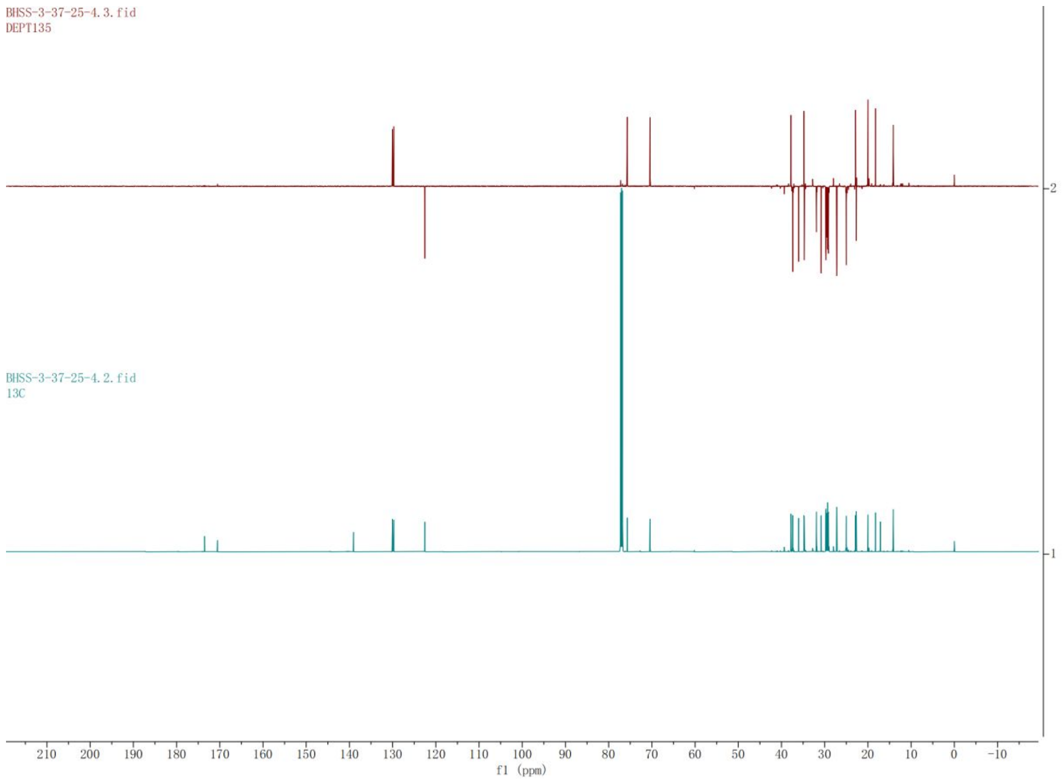


Figure S104. DEPT 135 spectrum (150 MHz) of carabrolate K (**11**) in $CDCl_3$

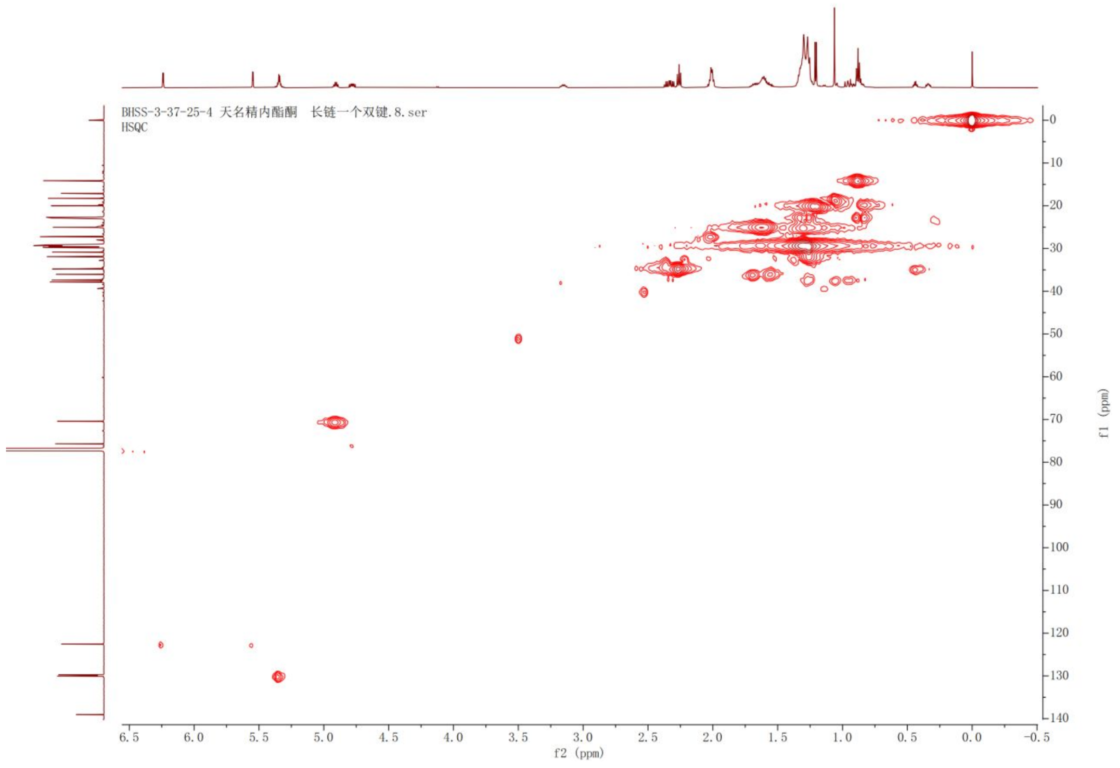


Figure S105. HSQC spectrum (600 MHz) of carabrolate K (**11**) in $CDCl_3$

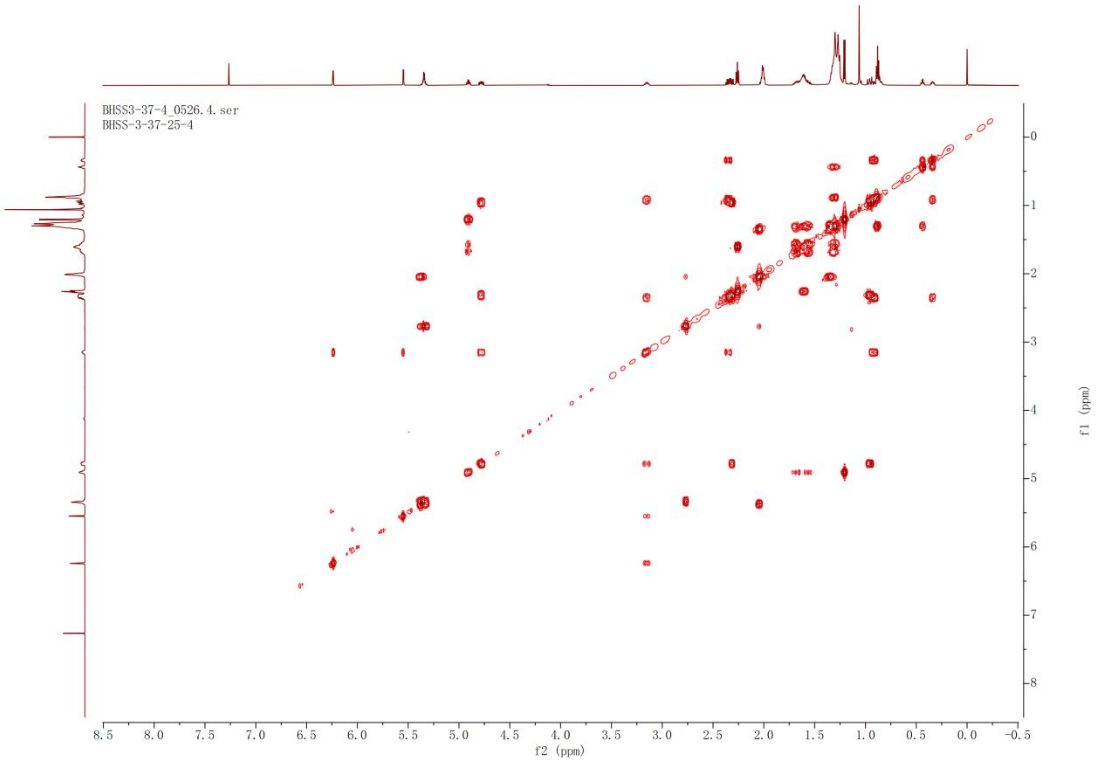


Figure S106. $^{1}H$-$^{1}H$ COSY spectrum (600 MHz) of carabrolate K (**11**) in $CDCl_3$

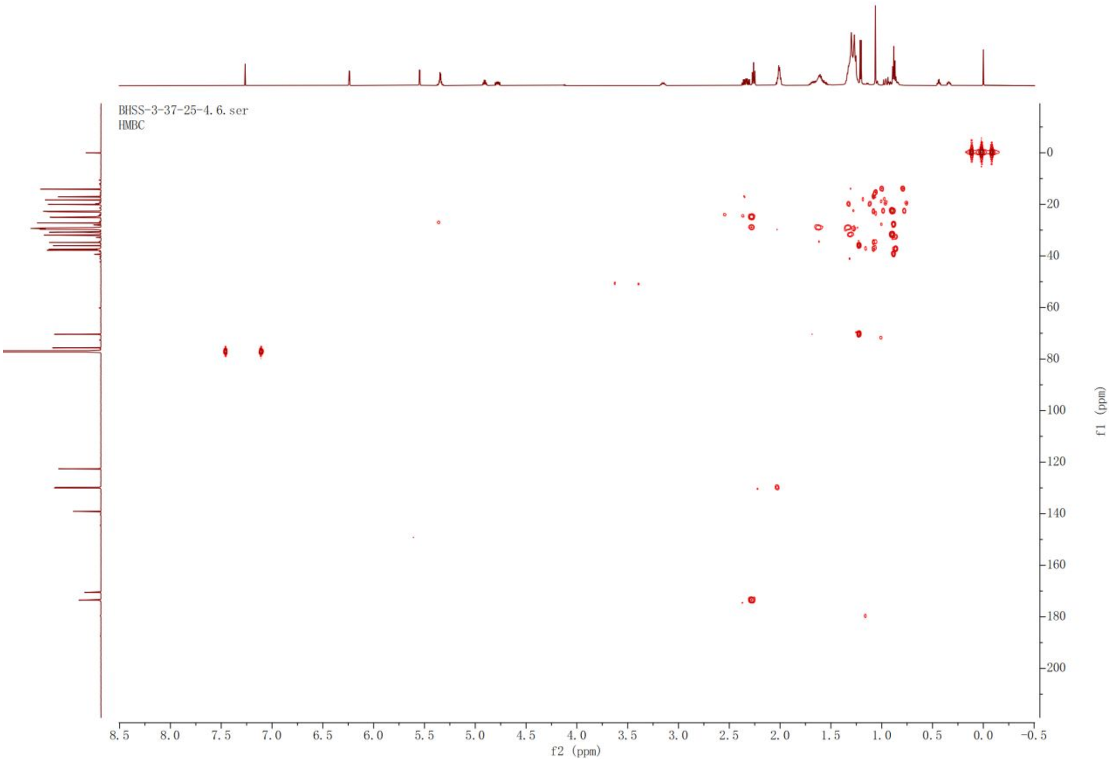


Figure S107. HMBC spectrum (600 MHz) of carabrolate K (**11**) in $CDCl_3$

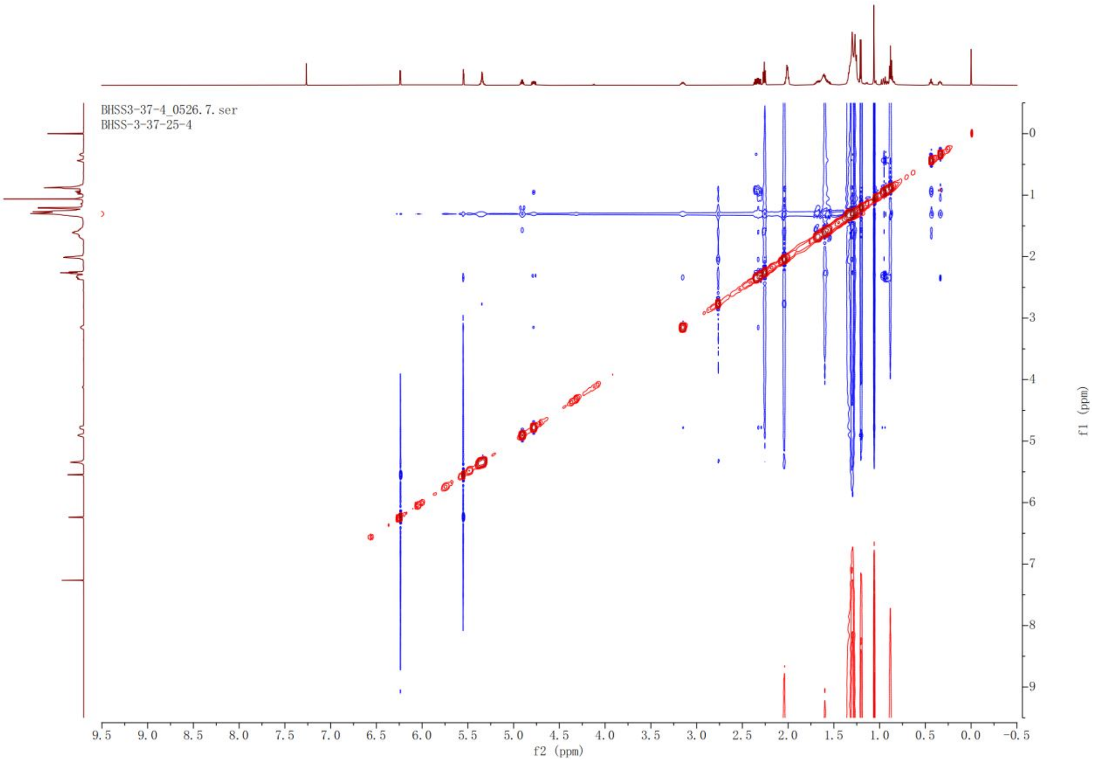


Figure S108. NOESY spectrum (600 MHz) of carabrolate K (**11**) in $CDCl_3$

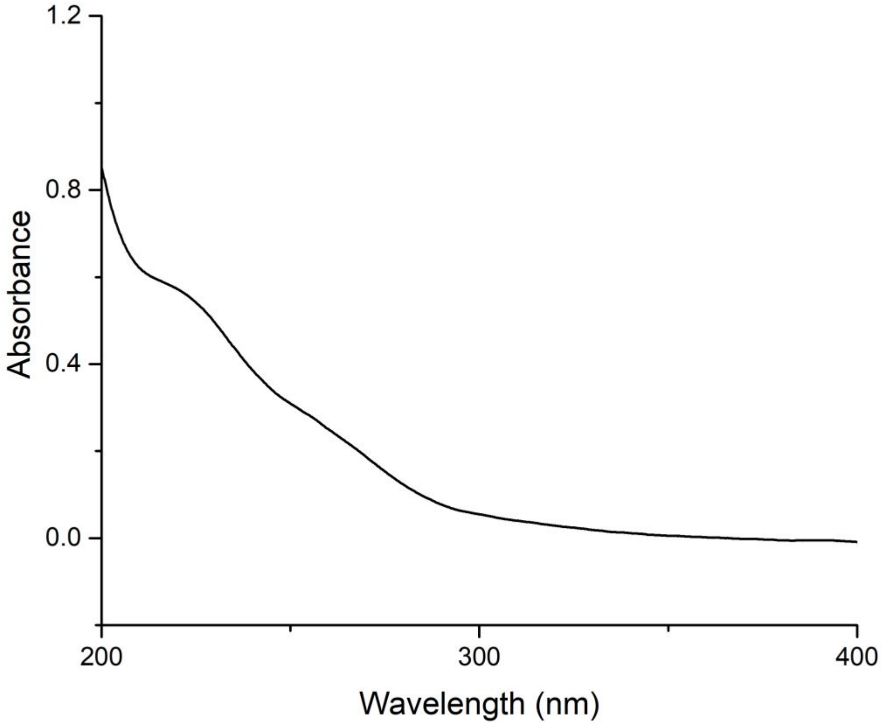


Figure S109. UV spectrum of carabrolate J (**10**) in MeOH

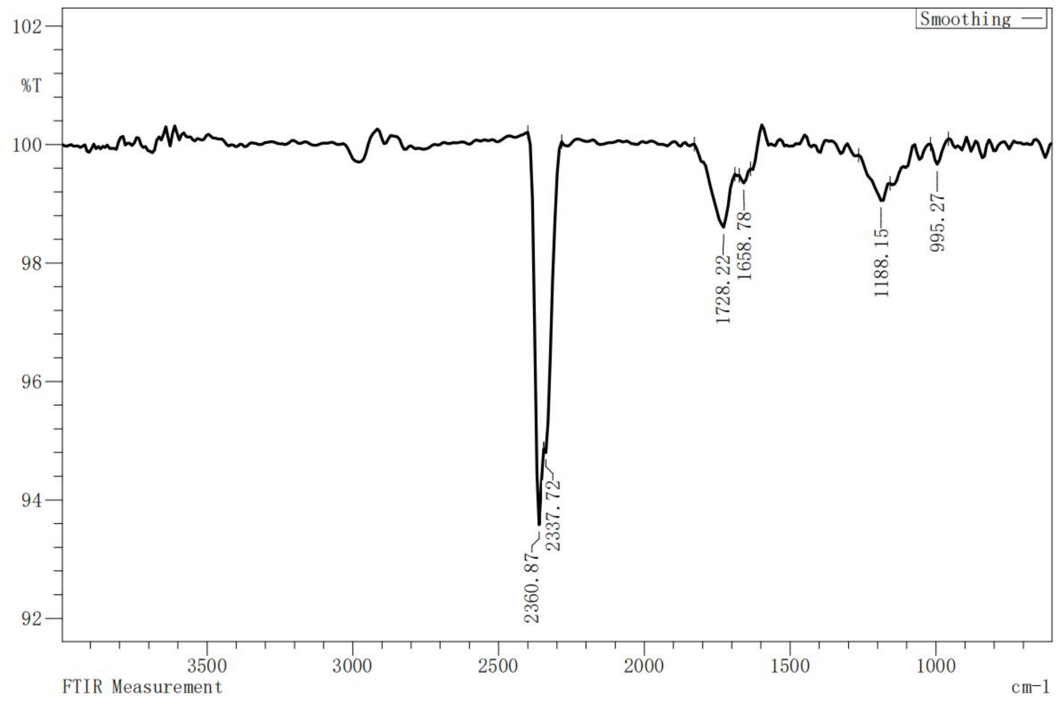


Figure S110. IR spectrum (film on KBr plate) of carabrolate K (**11**)

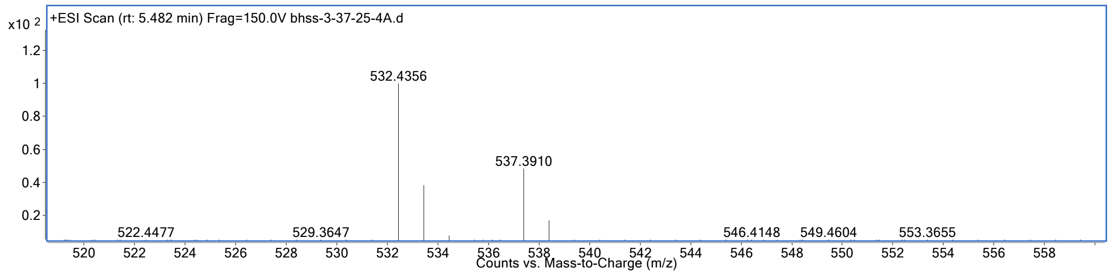


| Formula (M) | Score (MFG) | Mass | Mass (MFG) | m/z (Calc) | Diff (ppm) | Ion Formula | m/z |
|---|---|---|---|---|---|---|---|
| $C_{33}H_{54}O_4$ | 99.64 | 514.4018 | 514.4022 | 532.436 | 0.85 | $C_{33}H_{58}NO_4$ | 532.4356 |
| $C_{33}H_{54}O_4$ | 99.65 | 514.4018 | 514.4022 | 537.3914 | 0.84 | $C_{33}H_{54}NaO_4$ | 537.391 |

Figure S111. HRESIMS spectrum of carabrolate K (**11**)

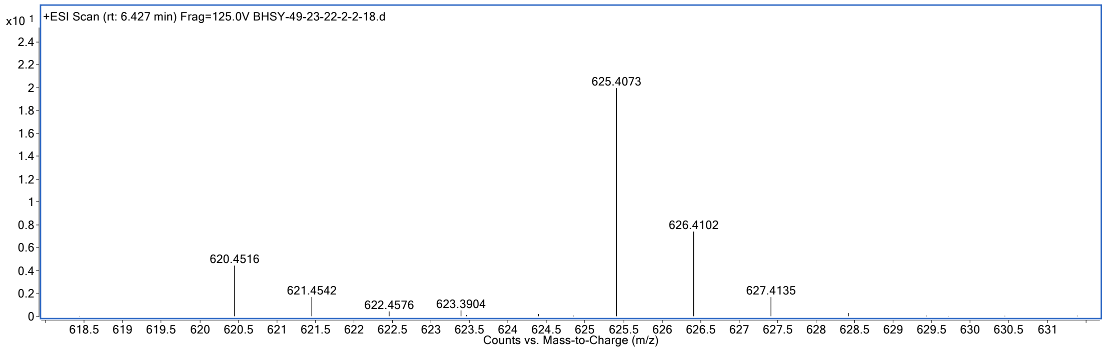


| Formula (M) | Score (MFG) | Mass | Mass (MFG) | m/z (Calc) | Diff (ppm) | Ion Formula | m/z |
|---|---|---|---|---|---|---|---|
| $C_{36}H_{58}O_7$ | 99.87 | 602.4178 | 602.4183 | 620.4521 | 0.8 | $C_{36}H_{62}NO_7$ | 620.4516 |
| $C_{36}H_{58}O_7$ | 99.98 | 602.4181 | 602.4183 | 625.4075 | 0.29 | $C_{36}H_{58}NaO_7$ | 625.4073 |

Figure S112. HRESIMS spectrum of the ketal of carabrolate E (**5**)

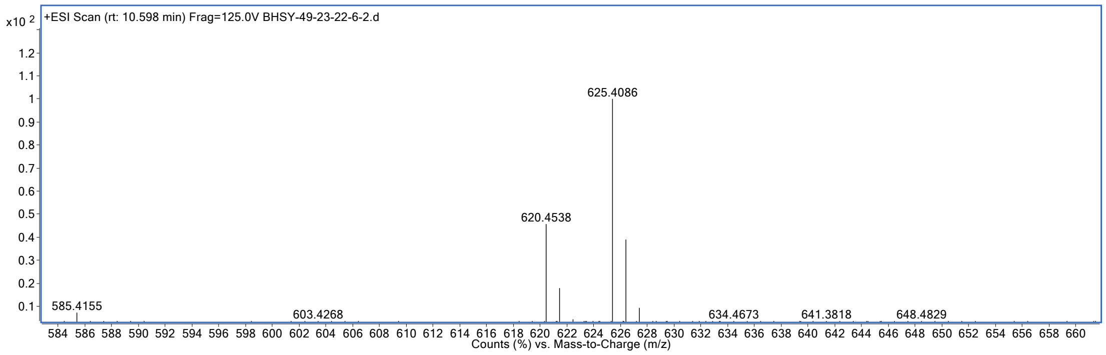


| Formula (M) | Score (MFG) | Mass | Mass (MFG) | m/z (Calc) | Diff (ppm) | Ion Formula | m/z |
|---|---|---|---|---|---|---|---|
| $C_{36}H_{58}O_7$ | 97.7 | 602.4195 | 602.4183 | 603.4255 | -2.11 | $C_{36}H_{59}O_7$ | 603.4268 |
| $C_{36}H_{58}O_7$ | 98.18 | 602.4194 | 602.4183 | 625.4075 | -1.87 | $C_{36}H_{58}NaO_7$ | 625.4086 |

Figure S113. HRESIMS spectrum of the ketal of carabrolate F (**6**)

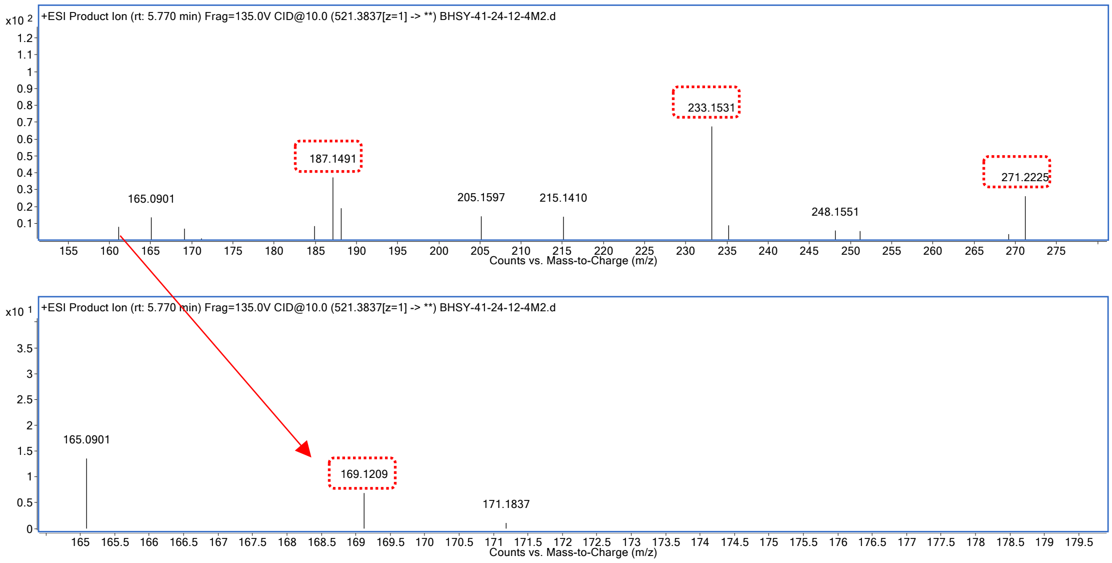


Figure S114. MS/MS spectrum (positive mode) of carabrolate G (**7**).

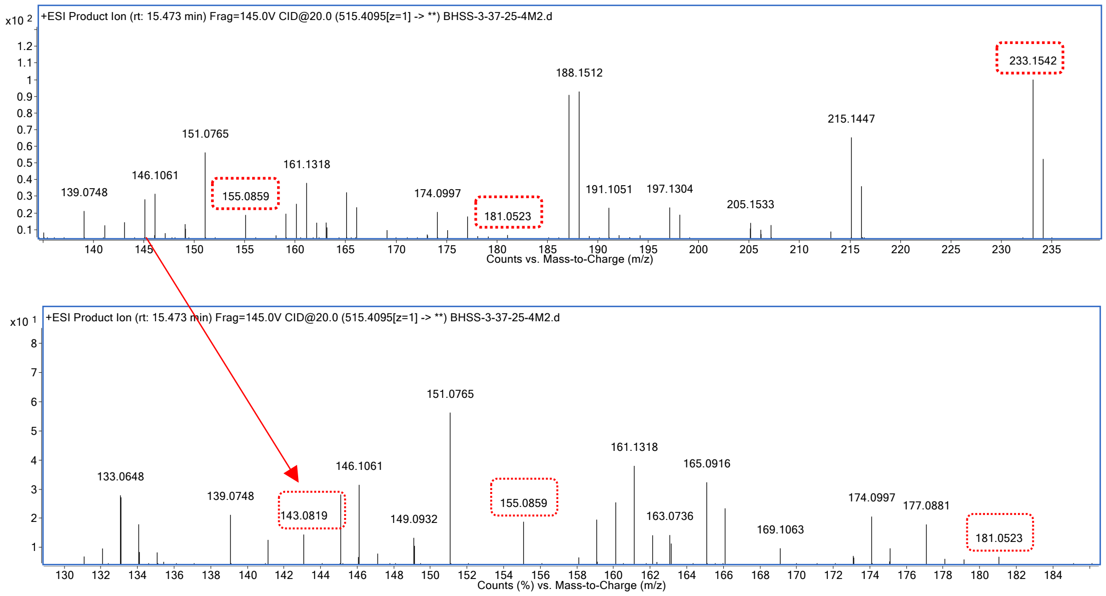


Figure S115. MS/MS spectrum (positive mode) of carabrolate K (**11**).

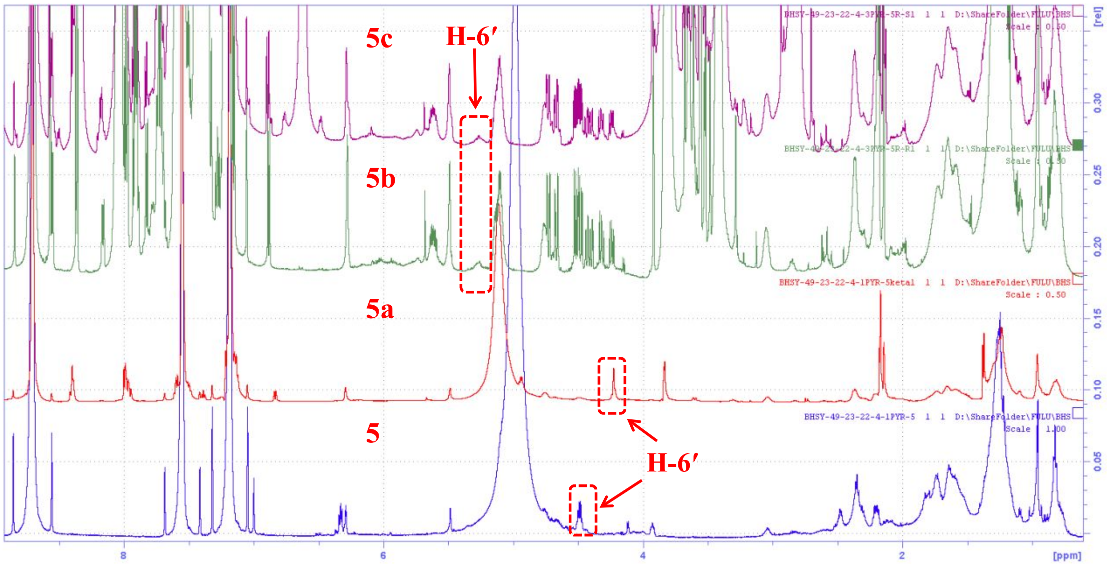


Figure S116. $^{1}$H NMR spectra (600 MHz) of **5**, **5a**, **5b**, and **5c** in $C_5D_5N$. **5a:** the ketal of **5**; **5b**: (*S*)-MTPA esters of **5a**; **5b**: (*R*)-MTPA esters of **5a**.

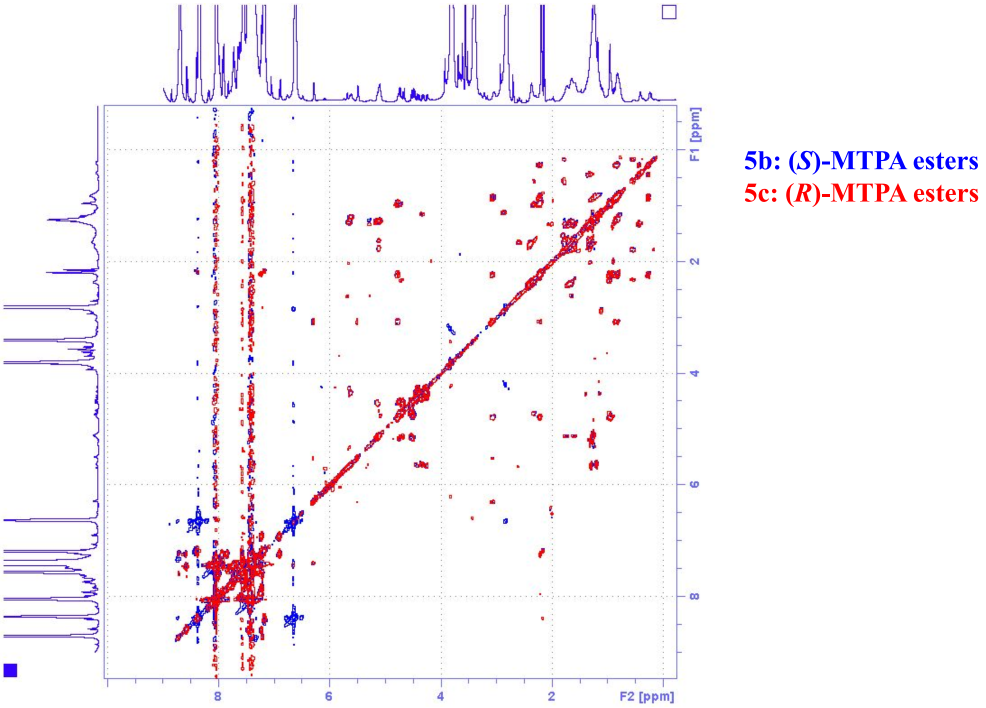


Figure S117. $^{1}$H-$^{1}$H COSY spectra (600 MHz) of **5b** and **5c** in $C_5D_5N$.

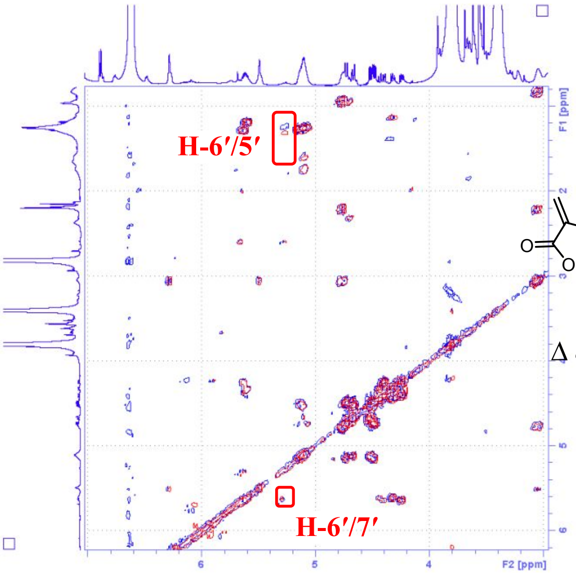


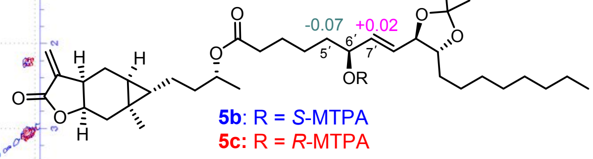


Δ $\delta_{S\text{-}R}$ values for the Mosher ester (**5b** and **5c**).

Figure S118. Enlarged $^1$H-$^1$H COSY spectra (600 MHz) of **5b** and **5c** in $C_5D_5N$.

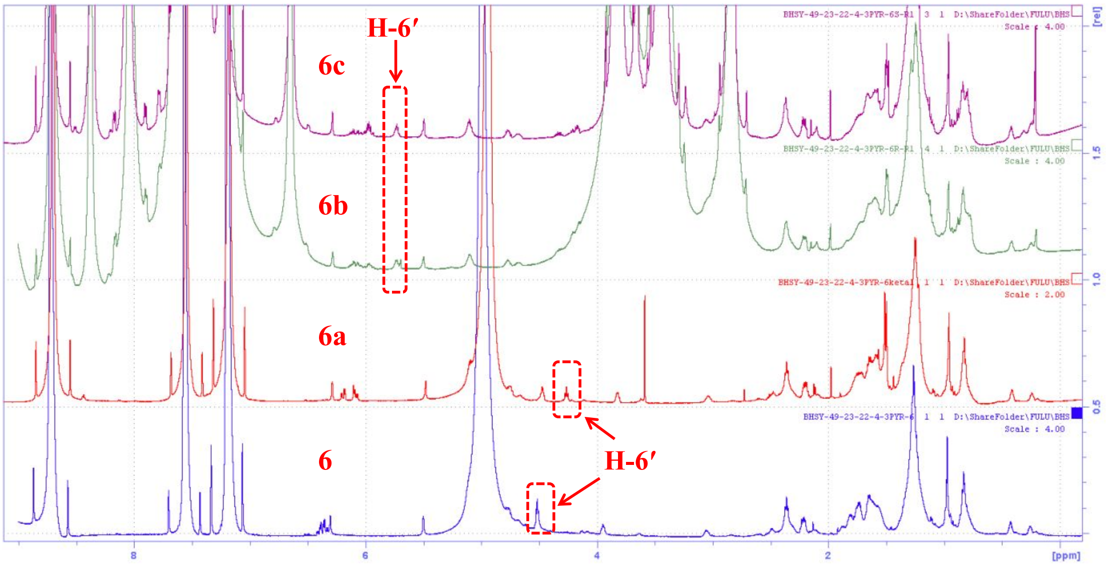


Figure S119. $^1$H NMR spectra (600 MHz) of **6**, **6a**, **6b**, and **6c** in $C_5D_5N$. **6a**: the ketal of **6**; **6b**: (*S*)-MTPA esters of **6a**; **6b**: (*R*)-MTPA esters of **6a**.

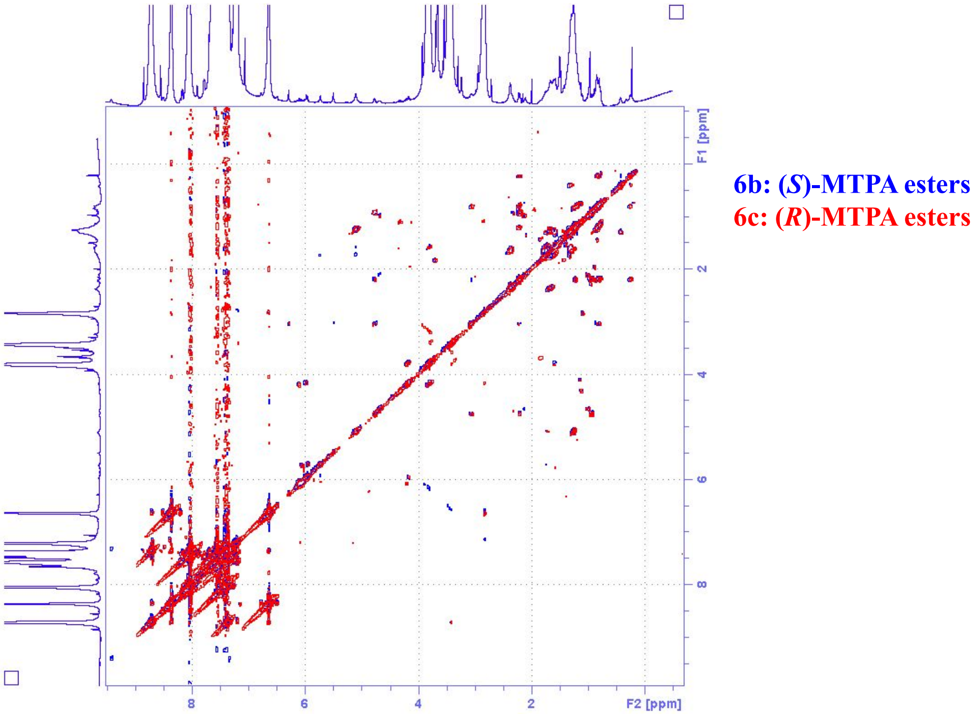


Figure S120. $^1$H-$^1$H COSY spectra (600 MHz) of **6b** and **6c** in $C_5D_5N$.

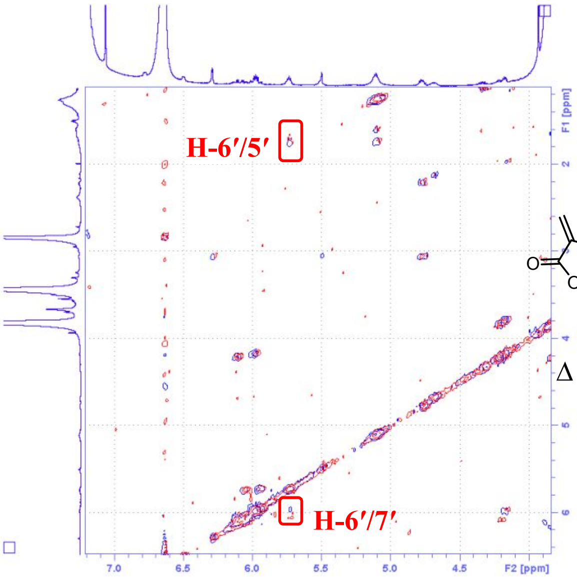


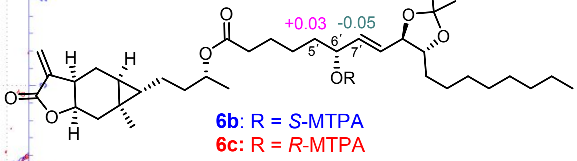


$\Delta\ \delta_{S\text{-}R}$ values for the Mosher ester (**6b** and **6c**).

Figure S121. Enlarged $^1$H-$^1$H COSY spectra (600 MHz) of **6b** and **6c** in $C_5D_5N$.

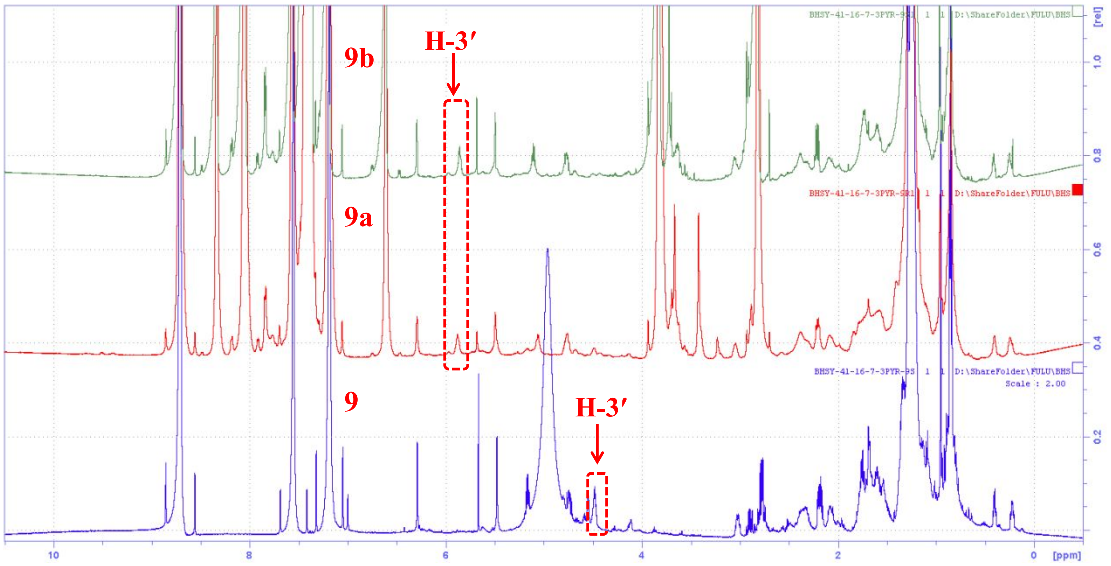


Figure S122. $^1$H NMR spectra (600 MHz) of **9**, **9a**, and **9b** in $C_5D_5N$. **9a**: (*S*)-MTPA esters of **9**; **9b**: (*R*)-MTPA esters of **9**.

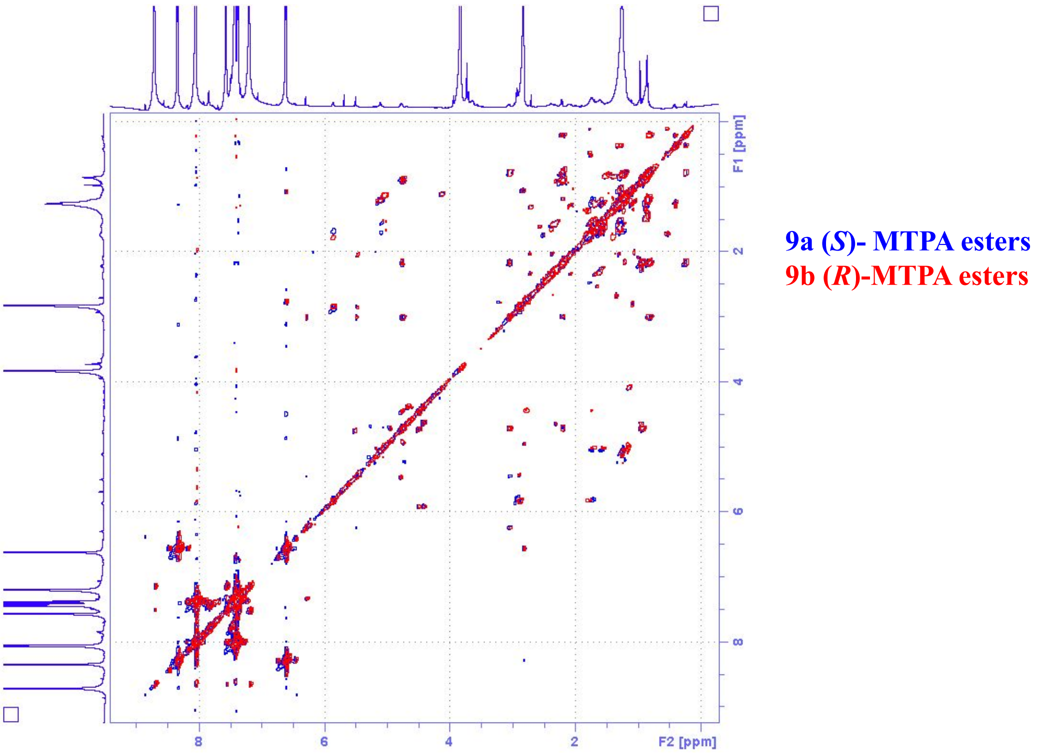


Figure S123. $^1$H-$^1$H COSY spectra (600 MHz) of **9a** and **9b** in $C_5D_5N$.

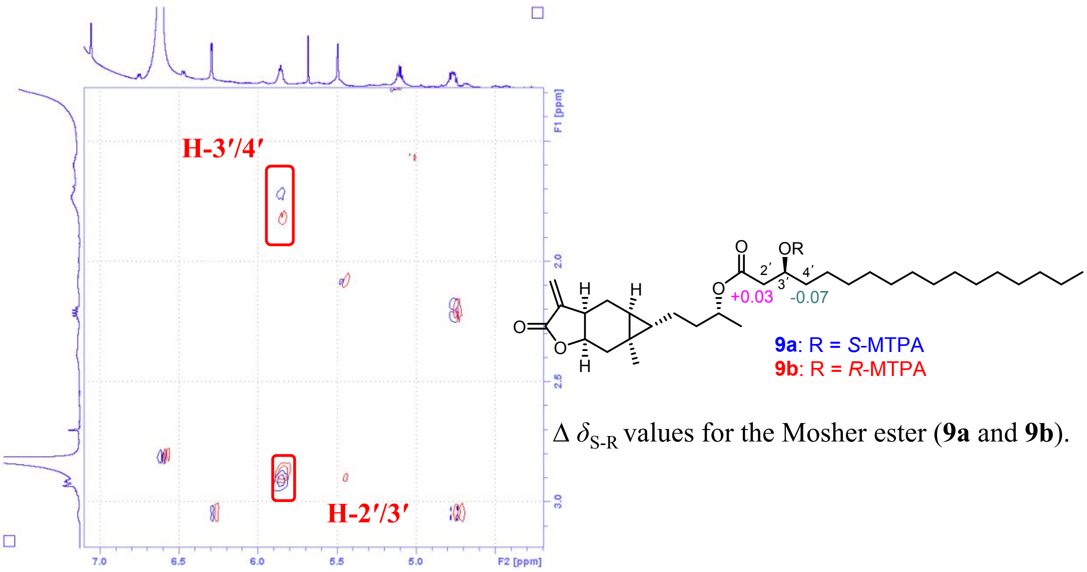


Figure S124. Enlarged $^{1}$H-$^{1}$H COSY spectra (600 MHz) of **9a** and **9b** in $C_5D_5N$.
